# Evaluating the Effects of Clinician Prescribing and Implementation Materials on Adoption of Virtual Reality Therapeutics: Randomized Feasibility Pilot Study

**DOI:** 10.2196/90626

**Published:** 2026-06-30

**Authors:** Ashlyn Zebrowski, R Jackson Fernandez, Cameron Pinkelton, Rebecca Kitzmiller, Adam W Kiefer, Laura Stanley, Gita Mody, Carlton Moore, Lukasz Mazur

**Affiliations:** 1 Carolina Health Informatics Program, School of Information and Library Science, University of North Carolina at Chapel Hill, 335 South Columbia Street, Chapel Hill, NC, 27514, United States, 1 9843282900; 2 Division of Healthcare Engineering, Department of Radiation Oncology, School of Medicine, University of North Carolina at Chapel Hill, Chapel Hill, NC, United States; 3 Division of Cardiothoracic Surgery, School of Medicine, University of North Carolina at Chapel Hill, Chapel Hill, NC, United States; 4 School of Nursing, University of North Carolina at Chapel Hill, Chapel Hill, NC, United States; 5 Department of Exercise and Sport Science, University of North Carolina at Chapel Hill, Chapel Hill, NC, United States; 6 Department of Computer Science, Gianforte School of Computing, Montana State University, Bozeman, MT, United States; 7 UNC Lineberger Comprehensive Cancer CenterChapel Hill, NC, United States

**Keywords:** virtual reality, digital therapeutics, human factors, usability, implementation science

## Abstract

**Background:**

Virtual reality therapeutics (VRx) are emerging as a form of digital therapeutics (DTx) with growing evidence for improving symptoms such as anxiety, pain, and distress. However, real-world adoption remains limited because successful use depends not only on the therapeutic benefits but also on how the intervention is introduced, supported, and integrated into care, in addition to the therapeutic benefits. Existing literature has identified barriers related to clinician familiarity, prescribing pathways, onboarding, and independent use outside the clinic, but few studies have experimentally evaluated how implementation strategies influence early adoption outcomes.

**Objective:**

This feasibility pilot study evaluated how different implementation strategies influence early adoption of a VRx intervention in a nonclinical setting by comparing unguided VRx use, self-directed VRx use with implementation materials, and VRx use with prescriber-led consultation and support.

**Methods:**

An institutional review board–approved, 3-arm randomized feasibility pilot study was conducted following the Virtual Reality Clinical Outcomes Research Experts framework and reported using the CONSORT (Consolidated Standards of Reporting Trials) 2010 extension guidelines for pilot and feasibility studies. Participants were randomly allocated to 1 of 3 experimental conditions. Outcomes included technology acceptance, usability, fidelity, and tolerability. Quantitative measures were complemented by qualitative exit interviews. Analyses were exploratory, and the study was not powered for hypothesis testing.

**Results:**

Across conditions, brief VRx exposure was associated with significant improvements in overall technology acceptance (*P*<.001), though postintervention scores remained in the neutral range, indicating early shifts rather than strong endorsement. Participants receiving provider-supported implementation demonstrated the most consistent pattern of favorable outcomes, including higher fidelity of use and significantly greater time-based adherence (*P*=.04). Usability scores were generally favorable across conditions. Tolerability was acceptable, with low levels of reported cybersickness and no discontinuations due to adverse effects. Qualitative findings highlighted the importance of guidance, expectation-setting, and perceived clinical legitimacy in shaping engagement.

**Conclusions:**

This study demonstrates that implementation strategy is a key determinant of adoption for virtual reality–based DTx. By experimentally isolating onboarding and provider support components, this work extends prior efficacy-focused research to address implementation design. Findings suggest that brief provider involvement and structured onboarding may enhance engagement and adherence, supporting future evaluation of scalable DTx implementation models in clinical and community settings.

## Introduction

### Background and Rationale

Anxiety, depression, and chronic pain are among the most prevalent and disabling health conditions globally, affecting hundreds of millions of people and contributing significantly to personal, economic, and societal burden [1-5]. Digital therapeutics (DTx), particularly virtual reality (VR)–based interventions (virtual reality therapeutics [VRx]), are emerging as clinically validated, nonpharmacological treatment options for a range of behavioral and neurological conditions [6-14]. Evidence indicates that VRx can reduce symptoms of anxiety, depression, and pain across diverse populations, including patients with cancer, older adults with chronic disease, and individuals with pain syndromes, in some cases, approaching the effectiveness of standard care [9-12,14-22]. By leveraging mechanisms such as cognitive distraction, immersive meditation, and behavioral engagement, VRx offers a scalable, patient-centered approach to symptom management that may improve access, adherence, and patient experience [9,14,23-29].

Despite promising evidence and reported acceptability, widespread adoption of DTx and VRx remains limited [21,30-35]. Barriers include insufficient provider training, unclear prescribing pathways, cost concerns, and inadequate support for patient onboarding and sustained use [12,25,27,28,36-38]. Additional challenges include workflow integration, technology limitations, and lack of structured implementation strategies [10,17,21,27,33,35,39,40]. While user-related factors such as engagement and personalization have shown positive influence on the adoption of digital interventions [32,39,41], provider behavior, organizational context, and implementation strategy are increasingly recognized as critical but underdeveloped drivers [28,34,42-44].

Many existing VRx studies do not incorporate implementation frameworks or assess perceptual and behavioral outcomes, focusing primarily on symptom efficacy [37,44-50]. As a result, there is limited insight into how patients and providers interact with VRx in real-world contexts, particularly during independent at-home use [7,11,51-54]. While prior studies suggest that patients can use VRx at home with initial training and remote support, most evidence is derived from structured or guided programs, leaving gaps in understanding unsupervised use, adherence, and integration into routine care [11,43,51,54-57]. Addressing these gaps requires experimental evaluation of implementation strategies that more closely reflect real-world use, particularly with respect to provider involvement and support structures.

Research in related areas underscores the importance of implementation structure for digital interventions. Factors such as guidance, integration into daily life, and social connectedness are key influences on engagement with digital health interventions [39], while provider training, resource allocation, and reimbursement remain barriers to VR adoption in clinical settings [58]. Organizational buy-in and workflow integration are also critical for facilitating successful implementation [59]. However, these studies provide limited insight into provider involvement or delivery models for at-home VRx use. Collectively, while evidence supporting digital and VR-based therapeutic interventions is growing, practical strategies for enabling scalable, real-world adoption remain insufficiently defined.

Implementation science provides a useful framework to address these challenges. The Behavior and Acceptance Framework (BEAR) supports systematic identification of barriers and facilitators influencing digital health implementation [60]. It integrates behavioral change theory and technology acceptance concepts to evaluate feasibility, acceptability, usability, and fidelity as key constructs for translating efficacy into real-world adoption [60]. Despite rapid growth in efficacy-focused VRx research, there remains limited experimental evidence evaluating how specific implementation strategies such as the BEAR affect real-world adoption.

Despite demonstrated clinical efficacy, many digital health technologies fail to scale due to barriers in implementation rather than therapeutic limitations. A key gap remains in understanding how provider engagement and structured implementation strategies impact DTx adoption. This study addresses this gap by evaluating how different implementation approaches affect acceptability, usability, and fidelity of VRx among healthy individuals in nonclinical settings. Participants were randomly allocated to one of three conditions: (1) unguided self-use, (2) self-directed use with asynchronous support materials, and (3) provider-supported onboarding with simulated prescription and guided setup. Acceptability and usability were assessed using the technology acceptance model (TAM) [61-63] and System Usability Scale (SUS) [64], and qualitative findings from exit interviews were mapped to BEAR [60] domains to identify implementation-related barriers and facilitators.

By systematically comparing these conditions, this study is, to our knowledge, one of the first to experimentally evaluate the impact of provider-led support versus self-directed implementation strategies on VRx adoption. These findings provide early evidence to inform scalable models for integrating DTx into routine care, particularly by clarifying the role of clinician involvement and structured support in enabling real-world use.

### Objectives

The objective of this feasibility pilot study was to evaluate how different implementation strategies influence early adoption of a VRx intervention in a nonclinical setting. Specifically, the study compared unguided VRx use, self-directed VRx use with implementation materials, and provider-supported VRx use with implementation materials to assess differences in acceptability, usability, fidelity, and tolerability. We hypothesized that participants receiving clinician-supported implementation would demonstrate more favorable adoption-related outcomes than those receiving self-directed support alone or no additional support.

## Methods

### Study Design

This was a 3-arm pilot study evaluating the acceptability, usability, and fidelity of a VR-based intervention (VRx) and accompanying implementation package across 3 experimental conditions. The study design was guided by the Virtual Reality Clinical Outcomes Research Experts framework [65], and the implementation package design was informed by the BEAR [60] and formative qualitative research with clinicians and end users. This study is reported in accordance with the CONSORT (Consolidated Standards of Reporting Trials) 2010 extension for pilot and feasibility studies [66]. The completed checklist is provided in Checklist 1. The CONSORT-eHEALTH (Consolidated Standards of Reporting Trials of Electronic and Mobile Health Applications and Online Telehealth) checklist [67] was also completed and is provided in Checklist 2.

Participants were allocated to 1 of 3 experimental conditions. As a pilot study, a formal power calculation for hypothesis testing was not performed. Consistent with guidance for feasibility research, approximately 10‐15 participants per study arm (total N=30‐45) were targeted for enrollment, which was considered sufficient to assess study procedures, characterize variability in key outcomes, and explore patterns across implementation conditions, while remaining appropriate for an early-stage study [68,69]. Accordingly, the study was not powered to detect statistically significant differences between groups, and findings should be interpreted as exploratory.

A mixed methods approach was used to provide a comprehensive assessment of how implementation strategies and provider engagement influence early adoption. Quantitative measures, such as task completion rates and structured questionnaires, assessed acceptability, usability, and fidelity, and enabled comparison across groups. Qualitative insights from exit interviews contextualized findings by capturing user perspectives, identifying usability challenges, and highlighting perceived benefits and areas for improvement.

### Participants

#### Overview

Study participants were recruited through university listservs, community announcements, and institutional study recruitment platforms. Recruitment and data collection took place between December 2024 and February 2025. Study data were collected and managed using REDCap (Research Electronic Data Capture; Vanderbilt University), hosted at the University of North Carolina at Chapel Hill [70,71].

Of the 39 participants enrolled, 31 (79.5%) completed the study and were included in the final analysis.

#### Eligibility Criteria

Participants were eligible for inclusion if they were adults aged 18‐65 years, were in good general health with no safety concerns for VR use, and had no history of psychiatric or neurological conditions that could be exacerbated by VR exposure (eg, epilepsy or seizure disorders, severe motion sensitivity, vestibular disorders, or conditions associated with sensory overstimulation). Additional inclusion criteria included limited prior VR experience (defined as fewer than 2 lifetime uses), the ability to provide informed consent, and a motion sickness score below 21.6 on the prescreening assessment. Full screening instruments, including VR safety and prior experience questions used for eligibility screening, are provided in Multimedia Appendix 1.

#### Study Setting

This study was conducted outside of a clinical setting, in either (1) a human factors laboratory designed to simulate a home-like environment or (2) a community-based setting (eg, private library study spaces and private community center spaces). Measures were taken to ensure privacy, confidentiality, and consistent study procedures across all settings.

### Adverse Events and Safety Monitoring

Screening procedures were used to minimize the risk of adverse events. Individuals who did not meet the inclusion criteria were not enrolled. No adverse events were reported during the study. Cybersickness susceptibility was assessed during screening using the Motion Sickness Susceptibility Questionnaire—Short Form [72]. Participants with severe susceptibility to motion sickness were excluded. Prior to VRx use and immediately following, participants were screened using the Cybersickness in Virtual Reality Questionnaire (CSQ-VR) [73] to minimize cybersickness risk and assess symptoms. Participants were instructed to discontinue use if symptoms occurred, with study personnel present to ensure safety.

### Randomization

Participants were randomly allocated to 1 of 3 experimental conditions with a target 1:1:1 allocation ratio. The allocation sequence was generated in Microsoft Excel using the RAND() function prior to study initiation. Simple randomization was used; no formal blocking or stratification was applied. Assignments were made by study team members at the time of scheduling, with each enrolled participant assigned to the next available condition in the allocation sequence. Because recruitment, scheduling, and data collection occurred in parallel over a defined recruitment window (December 2024-February 2025), target group sizes were monitored during enrollment; when a scheduled participant did not attend their session, a subsequent enrolled participant was assigned to the affected condition to maintain approximate group sizes across arms. Self-reported technology comfort level and prior VR experience were monitored as baseline characteristics but were not used as formal allocation variables.

Allocation concealment from study personnel was not implemented, as schedulers required visibility of assignments to prepare condition-specific materials in advance of each session. Full blinding of participants and study personnel was not feasible, given the unavoidably visible differences between study arms, for example, the presence of a provider in condition 3 and the presence of physical implementation materials in conditions 2 and 3. To minimize expectancy effects, participants were not informed of their assigned condition until arrival for their study appointment. The absence of allocation concealment and blinding is acknowledged as a limitation.

### Intervention

The intervention consisted of art therapy delivered using the application OpenBrush on an Oculus Quest 2 headset. Participants were instructed to complete a series of drawing tasks over a 20-minute session. Further information about OpenBrush and selection of the application is provided in Multimedia Appendix 2.

**Figure 1.**
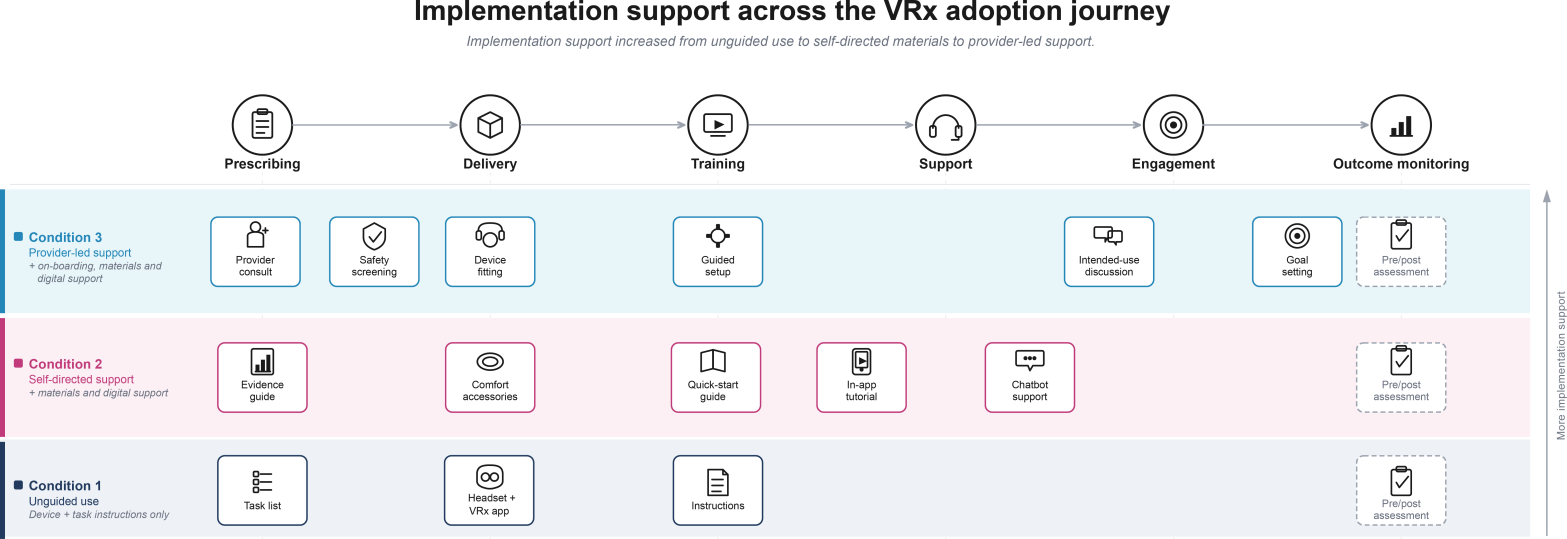
Conceptual overview of the implementation support solution components used across the VRx adoption journey in a 3-arm feasibility pilot study. All participants moved through the same 6 implementation stages, while support increased from unguided use to self-directed support materials and provider-led onboarding. Detailed condition-specific components are provided in Table 1 and Multimedia Appendix 3. VRx: virtual reality therapeutics.

**Figure 2.**
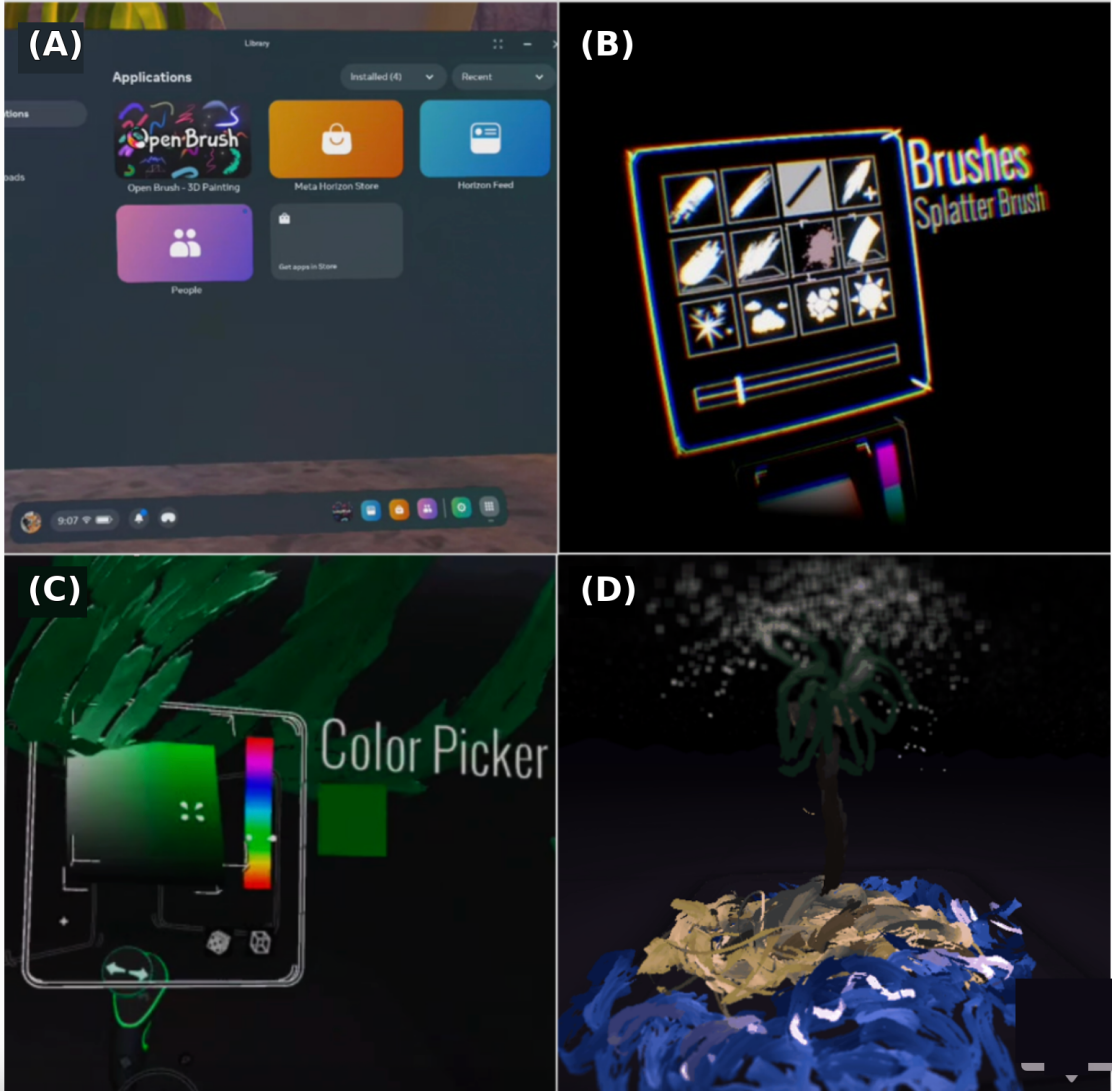
Representative images of the OpenBrush virtual reality application used in the study. (A) OpenBrush application shown in the headset application library. (B) In-app brush selection menu. (C) In-app color picker tool. (D) Example 3D drawing created in OpenBrush.

Condition-specific materials (eg, comfort accessories, tutorials, chatbot, or provider consultation) were provided to participants as outlined in Table 1. Detailed implementation package elements, equipment lists, and task protocols are provided in Multimedia Appendix 3. Differences between experimental conditions across stages of implementation are summarized in Figure 1. Representative screenshots of the VRx application interface are shown in Figure 2.

**Table 1.** Overview of experimental conditions and levels of implementation support in a 3-arm randomized feasibility pilot study of virtual reality therapeutics (VRx) conducted among healthy adults in nonclinical settings at the University of North Carolina at Chapel Hill (December 2024-February 2025).

Condition	Description	Intervention details
1. Unguided use	Self-directed use with no onboarding or support materials	Virtual reality headset preloaded with VRx application, minimal instructions
2. Self-directed support	Self-directed use with asynchronous support materials	Headset with VRx application and comfort accessories, quick start guides, tutorial, chatbot support
3. Provider-led support	Implementation package plus a simulated provider encounter	Same as condition 2, plus a 5-minute scripted provider consultation including mock diagnosis, virtual reality fitness check, and guided first use

The implementation package was developed based on prior qualitative research conducted by the study team exploring barriers to VRx adoption and was informed by the BEAR [60] and Virtual Reality Clinical Outcomes Research Experts framework [65] to address key implementation barriers across 6 stages of the implementation process. Package components included comfort accessories to improve device ergonomics, paper-based quick reference guides, a clinical evidence infographic, and an in-app step-by-step tutorial. The VR system menu was preconfigured for conditions 2 and 3 to display only the therapeutic application, reducing navigation complexity. A support chatbot was developed to provide real-time troubleshooting assistance. A complete description of all implementation package components, including photographs, equipment specifications, training documents, and the chatbot development process, is provided in Multimedia Appendix 3.

In the provider-supported condition, participants engaged in a standardized, scripted interaction delivered by a trained research team member with clinical training (eg, medical students). The interaction simulated a brief clinical encounter introducing VRx, including an explanation of the intervention, expected benefits, guidance on use, and a brief safety screening and device fitting. Interactions were time-limited (<5 minutes) and delivered using a predefined script to ensure consistency, as provided in Multimedia Appendix 3.

All individuals delivering the interaction were trained on the script and protocol prior to study initiation, and the script was followed closely to ensure standardized delivery. A checklist-based approach was used to ensure that all key components of the interaction were delivered consistently across participants. The provider consultation script and materials were developed based on prior qualitative research conducted by the study team exploring clinician perspectives on DTx and VRx and were designed to simulate a realistic clinical interaction.

### Data Collection

Participants completed questionnaires at session initiation and immediately following intervention use. All measurement instruments administered in this study, including screening tools and outcome measures, are provided in Multimedia Appendix 4. Observational data, including task performance, errors, help requests, and adherence, were recorded by trained observers using a structured checklist (Multimedia Appendix 5). For conditions 2 and 3, the use of implementation system components and the time spent were also recorded.

Exit interviews were conducted immediately following the session to capture user perceptions, barriers, and facilitators related to intervention use.

Primary outcomes included acceptability, usability, and fidelity; tolerability was assessed as a secondary outcome. Validated measurement instruments and techniques were used to collect both subjective and objective data, which are summarized in Table 2.

**Table 2.** Summary of measurement instruments used in a 3-arm pilot study of virtual reality therapeutics conducted from December 2024 to February 2025^a^.

Construct	Measurement	Framework	Classification
Acceptability	TAM3^b^-based pre- or postquestionnaires, time in virtual reality, behavioral observations	TAM [61-63]	Subjective+objective
Usability	Task completion, error rates, help requests, time to complete, SUS^c^ scores	SUS [64], task analysis	Objective+subjective
Fidelity	Adherence to protocol, task completion, duration, motivation (time engaged)	Adherence metrics	Objective
Tolerability	Pre- or postcybersickness questionnaire	CSQ-VR^d^ [73]	Subjective
Qualitative insights	Exit interviews	BEAR^e^ [60]	Subjective

a Measures collected subjective and objective data at 3 time points: during screening, at session initiation, and immediately following intervention use.

b TAM: technology acceptance model.

c SUS: System Usability Scale.

d CSQ-VR: Cybersickness in Virtual Reality Questionnaire.

e BEAR: Behavior and Acceptance Framework.

### Data Analysis

#### Acceptability

Acceptability was assessed using a TAM-based questionnaire administered before and after the intervention, capturing perceived usefulness (PU), perceived ease of use (PEU), attitude toward technology (ATT), and behavioral intention to use (BI). Questions used a 5-point Likert scale for response.

Objective indicators of acceptability included time spent in VR and behavioral engagement during the session.

#### Usability

Usability was evaluated using both objective and subjective measures, including task completion rates, error frequency, help requests, time to complete tasks, and SUS scores [64,74,75].

A modified SUS was used, with 2 items removed due to the limited complexity of the system. Scores were rescaled to a 0‐100 range. Given this modification, results are interpreted descriptively and are not directly comparable to standard SUS benchmarks.

#### Fidelity

Fidelity was assessed through adherence to the study protocol, including task completion, duration of engagement, and time-based adherence. Observational measures captured whether participants followed intended workflows versus engaging in exploratory behaviors.

#### Tolerability

Tolerability was assessed using the CSQ-VR [73], administered before and after the intervention. This measure assessed participant perceptions of discomfort and was used to monitor for potential adverse events and evaluate the role of preintervention screening in supporting safe VRx use.

#### Qualitative Insights

Exit interviews were conducted using a structured guide informed by the BEAR [60] to capture participant perceptions, barriers, and facilitators to VRx use. Following the approach of Braun and Clarke [76] to reflective thematic analysis, responses were evaluated using an iterative, inductive process including familiarization with the data through repeated reading of the interview notes, generation of initial codes, and grouping of codes into themes. Themes were then iteratively reviewed, refined, and defined to capture patterned meaning across participant experiences. Resulting themes were mapped to constructs from the BEAR to contextualize findings relative to implementation-relevant barriers and facilitators.

### Statistical Analysis

#### Overview

Data normality was assessed using Shapiro-Wilk tests. As several variables violated normality assumptions, nonparametric statistical tests were used.

Consistent with pilot study recommendations, analyses were structured to characterize the direction and magnitude of effects rather than to test formal hypotheses. Analyses were categorized as primary, secondary, or exploratory. All statistical analyses were conducted using R software (R Foundation for Statistical Computing).

#### Primary Analysis

Pre- and postchanges in overall TAM scores were assessed using Wilcoxon signed rank tests. Effect sizes were calculated as *r*=*Z*/√N, with 95% CIs estimated using bias-corrected and accelerated bootstrapping (2000 resamples).

Observational and behavioral data (eg, task performance, adherence, and engagement time) were summarized using descriptive statistics.

#### Secondary Analysis

Between-group differences in acceptability, usability, and fidelity outcomes were evaluated using Kruskal-Wallis tests. Where significant, post-hoc pairwise comparisons were conducted using Wilcoxon rank sum tests with Bonferroni-adjusted *P* values. TAM construct-level pre- and postchanges (PU, PEU, ATT, and BI) were assessed across the full sample using Wilcoxon signed rank tests. Overall TAM pre- and postchange was additionally examined within each experimental condition. Effect sizes were calculated as *r*=*Z*/√N, with 95% CIs estimated using bias-corrected and accelerated bootstrapping (2000 resamples).

#### Exploratory Analysis

Construct-level TAM analyses by experimental condition and SUS item-level analyses were conducted on an exploratory basis. SUS item-level analyses were conducted descriptively to characterize usability patterns across conditions. No corrections for multiple comparisons were applied to exploratory analyses, and findings should be interpreted based on overall patterns rather than individual construct-level comparisons. Additional exploratory subgroup analyses examined associations between participant characteristics and outcomes.

#### Modified SUS

A modified SUS [64] was used, with 2 items removed due to limited system complexity: “I found the various functions in this system were well integrated” and “I thought there was too much inconsistency in this system.” Scores were rescaled to a 0‐100 range. Analyses were conducted at the total score level; item-level responses were interpreted descriptively. This modification may limit direct comparability to standard SUS scores and benchmarks and is acknowledged as a limitation of the study.

#### Multiple Comparisons

Bonferroni corrections were applied to post-hoc pairwise comparisons. No corrections were applied to within-group or exploratory analyses, consistent with the hypothesis-generating nature of pilot studies [77].

Exploratory subgroup analyses examining associations between participant demographic characteristics (eg, age, education level, technology comfort, prior VR experience) and primary outcomes were conducted to assess for potential confounding; these analyses are reported in Multimedia Appendix 6. The associated risk of inflated type I error for uncorrected comparisons is acknowledged in the Limitations section.

### Ethical Considerations

This study was reviewed and approved by the UNC Institutional Review Board (IRB) at The University of North Carolina at Chapel Hill (IRB: 24‐2894). The study was determined to meet criteria for approval under federal regulations governing human subjects research, and all procedures were conducted in accordance with applicable ethical guidelines. All participants provided informed consent prior to enrollment. The consent process included a detailed explanation of the study purpose, procedures, risks, and the voluntary nature of participation. The informed consent form is provided in Multimedia Appendix 1. To protect participant privacy and confidentiality, all data collected were deidentified and stored securely using REDCap [70,71]. Personally identifiable information was removed from all research records, and data were stored without participant names. No identifying information is disclosed in any publications resulting from this research. Figures depicting the VR intervention (Figure 2) show application screenshots only, and no photographs of participants were included in the multimedia appendices. Participants did not receive financial compensation for their participation in this study. This study was not prospectively registered, as it was designed as a pilot feasibility study focused on evaluating implementation strategies and process outcomes (eg, acceptability, usability, and fidelity) rather than clinical efficacy or health outcomes. This approach is consistent with guidance indicating that formative and feasibility studies examining implementation processes and user perceptions do not require trial registration. All study procedures and outcome measures were predefined in the IRB-approved protocol prior to data collection, and no outcomes were modified after enrollment began.

## Results

### Participant Characteristics

Of the 39 participants enrolled, 31 (79.5%) completed the study and were included in the final analysis. In total, 8 participants did not complete the study due to logistical factors (n=6) or study-related factors (n=2), specifically the lack of monetary incentive.

**Figure 3.**
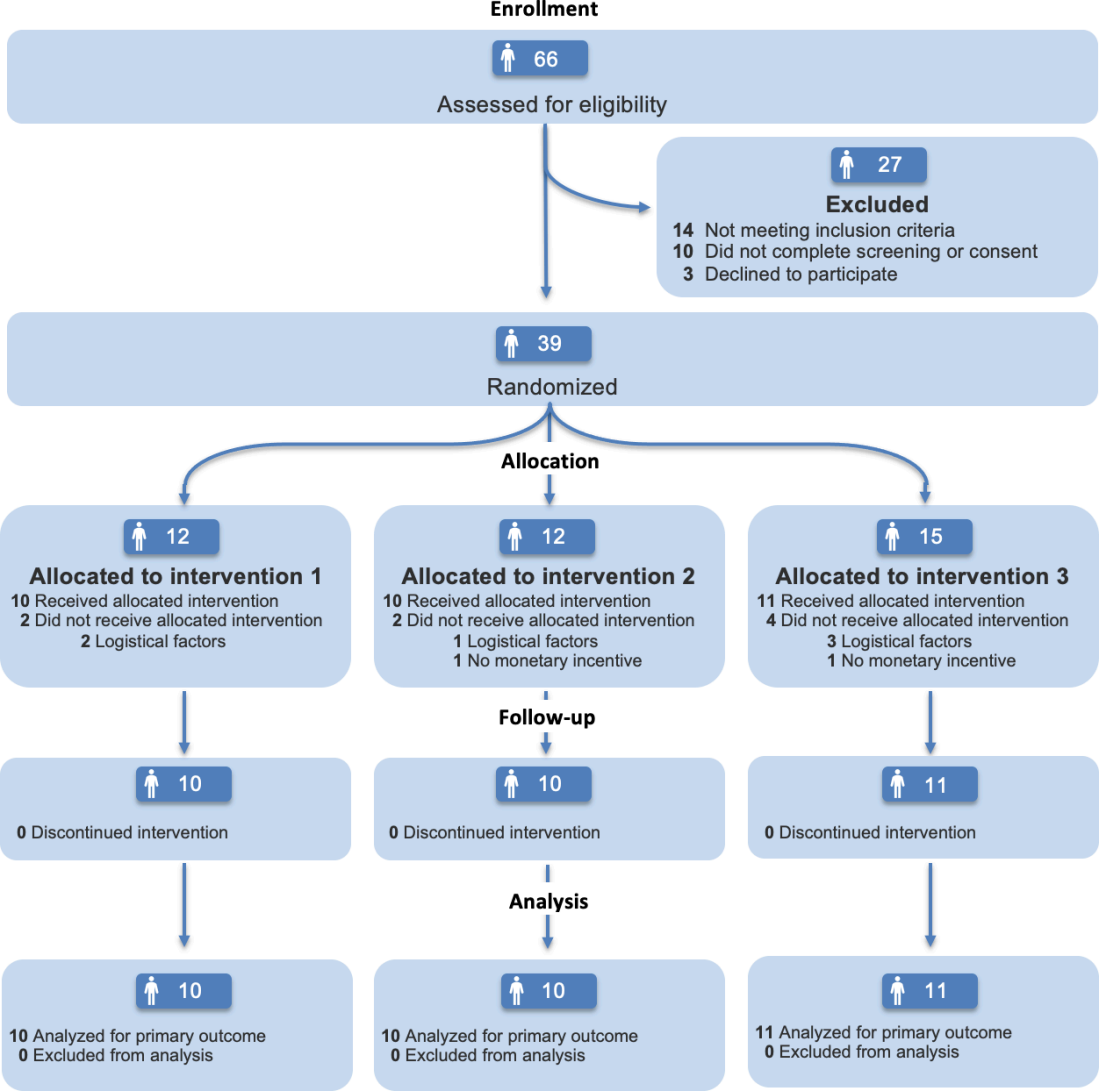
CONSORT flow diagram [78] illustrating participant progress through enrollment, intervention allocation, follow-up, and data analysis in a 3-arm pilot study of virtual reality therapeutics conducted from December 2024 to February 2025. CONSORT: Consolidated Standards of Reporting Trials.

Participants had a mean age of 38.6 (SD 12.3; range 18‐64) years, and the sample was predominantly female (20/31, 64.5%). Figure 3 presents a CONSORT-style participant flow diagram, and Table 3 summarizes the demographic characteristics by experimental condition.

**Table 3.** Demographic characteristics of study participants in a 3-arm pilot study of virtual reality (VR) therapeutics conducted from December 2024 to February 2025.

Characteristic	Overall (N=31)	Condition 1 (n=10)	Condition 2 (n=10)	Condition 3 (n=11)
Age (years), mean (SD)	38.6 (12.3)	37.3 (12.6)	40.9 (12.6)	37.6 (12.6)
Sex, n (%)				
Female	20 (64.5)	4 (40)	7 (70)	9 (81.8)
Male	10 (32.3)	6 (60)	3 (30)	1 (9.1)
Other	1 (3.2)	0 (0)	0 (0)	1 (9.1)
Education, n (%)				
High school diploma or GED	2 (6.5)	1 (10)	1 (10)	0 (0)
Some college, no degree	3 (9.68)	2 (20)	0 (0)	1 (9.1)
Associate degree	1 (3.3)	0 (0)	0 (0)	1 (9.1)
Bachelor degree	7 (22.6)	2 (20)	3 (30)	2 (18.1)
Master degree	13 (41.9)	3 (30)	4 (40)	6 (54.5)
Doctoral or professional degree	5 (16.1)	2 (20)	2 (20)	1 (9.1)
Employment, n (%)				
Employed full-time	17 (54.8)	6 (60)	5 (50)	6 (54.5)
Employed part-time	4 (12.9)	1 (10)	2 (20)	1 (9.1)
Self-employed	3 (9.7)	1 (10)	2 (20)	0 (0)
Student	5 (16.1)	2 (20)	1 (10)	2 (18.2)
Unemployed, looking for work	1 (3.2)	0 (0)	0 (0)	1 (9.1)
Unemployed, not looking for work	1 (3.2)	0 (0)	0 (0)	1 (9.1)
Overall technology comfort level, n (%)				
Low	16 (51.6)	4 (40)	6 (60)	6 (54.5)
Medium	15 (48.4)	6 (60)	4 (40)	5 (45.5)
Prior VR experience, n (%)				
Somewhat familiar	15 (48.4)	6 (60)	3 (30)	6 (54.5)
Never used	14 (45.2)	4 (40)	5 (50)	5 (45.5)
N/A—never heard of	2 (6.45)	0 (0)	2 (20)	0 (0)

Participant characteristics, including self-reported technology comfort and prior VR experience, were monitored during enrollment to assess balance across conditions. Participants were categorized as having low (16/31, 51.6%) or medium (15/31, 48.4%) technology comfort levels. Participants were assigned to experimental conditions according to a prespecified allocation sequence, with condition assignments made at the time of scheduling. Group comparisons indicated no significant differences in baseline demographic characteristics, including age, education level, or employment status.

Exploratory subgroup analyses examining associations between participant demographic characteristics and primary outcomes are reported in Multimedia Appendix 6. With the exception of acceptability scores, which differed significantly across age groups (*χ*^2^_4_=10.1; *P*=.04; *ηH*^2^=0.235), no significant associations were identified. Given the small subgroup sizes and exploratory nature of this analysis, the age-related acceptability finding should not be interpreted as evidence of confounding and warrants further investigation in future adequately powered studies.

### Acceptability Outcomes

#### Overall TAM Results

**Figure 4.**
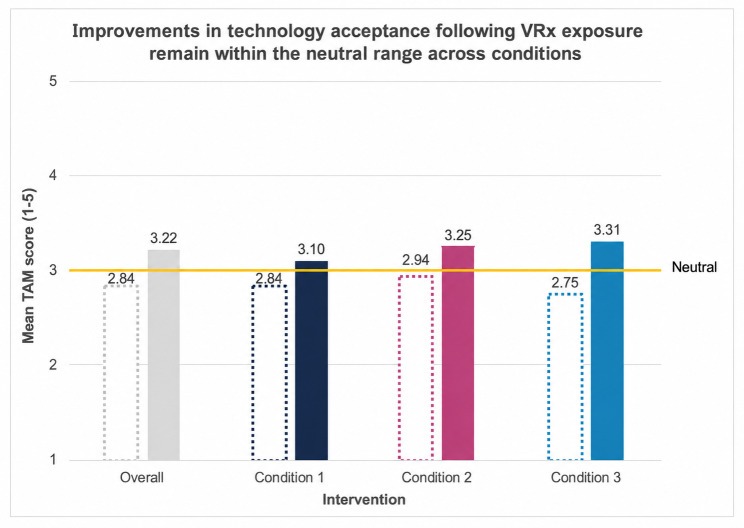
Pre- and postintervention TAM scores by experimental condition in a 3-arm randomized feasibility pilot study of VRx conducted from December 2024 to February 2025. Mean TAM scores increased across all conditions following intervention exposure; however, postintervention scores remained near the midpoint of the scale, indicating moderate rather than high levels of acceptance. TAM: technology acceptance model; VRx: virtual reality therapeutics.

Overall, technology acceptance demonstrated statistically significant increases following intervention exposure across the full sample (Figure 4). Mean TAM scores increased from 2.84 (SD 0.32) before the intervention to 3.22 (SD 0.45) after the intervention, representing a statistically significant improvement with a large effect size (*P*<.001; *r*=0.75, 95% CI 0.64-0.82; Table 4). However, absolute postintervention scores remained near the midpoint of the scale, indicating overall neutral to moderate levels of acceptance rather than high acceptance.

**Table 4.** Pre- and postchanges in overall mean technology acceptance model scores by experimental condition (N=31) in a 3-arm randomized feasibility pilot study of virtual reality therapeutics conducted from December 2024 to February 2025^a^.

Condition	Values, n	Preintervention, mean (SD)	Postintervention, mean (SD)	Δ Mean	*W*	*P* value^b^ (Wilcoxon)	Effect size, *r* (95% CI)
Condition 1 unguided use	10	2.84 (0.40)	3.10 (0.53)	+0.26	0	.03	0.70 (0.42-0.80)
Condition 2 self-directed support	10	2.94 (0.27)	3.25 (0.49)	+0.31	4	.03	0.70 (0.14-0.85)
Condition 3 provider-led support	11	2.75 (0.29)	3.31 (0.34)	+0.56	0	.005	0.85 (0.66-0.89)
Overall sample	31	2.84 (0.32)	3.22 (0.45)	+0.38	7	<.001	0.75 (0.64-0.82)

a Technology acceptance model scores are based on a Likert scale from 1 to 5. Pre- and postdifferences were assessed using Wilcoxon signed rank tests. Effect sizes are reported as *r*=*Z*/√N. 95% CIs were estimated using bias-corrected and accelerated bootstrapping (2000 resamples). Δ Mean represents the difference between preintervention and postintervention scores. This table reflects primary (overall sample) and secondary (condition-level) analyses.

b *P* values are reported for all comparisons; statistical significance was defined as *P*<.05.

At the intervention condition level, statistically significant improvements were observed across all 3 experimental conditions (Table 4). The largest improvement was observed in condition 3 (provider-led support), followed by condition 2 (self-directed support) and condition 1 (unguided use). These findings suggest that more structured implementation approaches were associated with greater improvements in technology acceptance. Despite these improvements, absolute TAM scores across all conditions remained near the midpoint of the 5-point scale, indicating that while acceptability shifted from slightly below neutral to slightly above neutral, none of the conditions achieved a high level of acceptance.

TAM construct-level changes are summarized in Table 5; constructs examined included PU, PEU, ATT, and BI [61-63]. Across the full sample, all constructs demonstrated statistically significant improvements following intervention exposure. Effect sizes were large for PU, ATT, and BI (*r*=0.64, 0.63, and 0.67, respectively) and medium for PEU (*r*=0.48). Mean PU increased by 0.40 from 2.87 (SD 0.41) to 3.27 (SD 0.53), mean PEU increased by 0.30 from 2.89 (SD 0.53) to 3.19 (SD 0.56), mean ATT increased by 0.37 from 3.02 (SD 0.46) to 3.39 (SD 0.50), and mean BI increased by 0.45 from 2.58 (SD 0.41) to 3.03 (SD 0.64). The largest absolute improvement was observed in BI, followed by PU and ATT, while PEU demonstrated a more modest increase in comparison.

**Table 5.** Pre- and postchanges in overall mean technology acceptance model (TAM) scores at the construct level in a 3-arm randomized feasibility pilot study of virtual reality therapeutics conducted from December 2024 to February 2025^a^.

TAM construct	Preintervention, mean (SD)	Postintervention, mean (SD)	Δ Mean	*W*	*P* value^b^ (Wilcoxon)	Effect size, *r* (95% CI)
Perceived usefulness	2.87 (0.41)	3.27 (0.53)	+0.40	0	<.001	0.64 (0.51-0.74)
Perceived ease of use	2.89 (0.53)	3.19 (0.56)	+0.30	35	.007	0.48 (0.14-0.68)
Attitude toward technology	3.02 (0.46)	3.39 (0.50)	+0.37	0	.001	0.63 (0.49-0.72)
Behavioral intention to use	2.58 (0.41)	3.03 (0.64)	+0.45	16	<.001	0.67 (0.43-0.78)
Overall TAM	2.84 (0.32)	3.22 (0.45)	+0.38	7	<.001	0.75 (0.64-0.82)

a Values represent the mean pre- and postintervention TAM scores and corresponding mean change (Δ mean) within each TAM construct. TAM scores are based on a Likert scale (range 1-5). Pre- and postdifferences were assessed using Wilcoxon signed rank tests. Effect sizes are reported as *r*=*Z*/√N. 95% CIs were estimated using bias-corrected and accelerated bootstrapping with 2000 resamples. Δ Mean represents the difference between preintervention and postintervention scores. This table reflects TAM construct-level analyses conducted across the overall sample (N=31).

b *P* values are reported for all comparisons; statistical significance was defined as *P*<.05.

Despite these statistically significant improvements across all constructs, postintervention scores remained near the midpoint of the scale (~3.0‐3.4), indicating moderate rather than high levels of acceptance. While improvements reflect a greater openness to VRx, postintervention results for intention to use remained comparatively lower than other constructs, suggesting that increased acceptance did not fully translate into strong intention to adopt.

#### Exploratory Between-Condition Construct-Level Results

Exploratory construct-level changes by experimental condition are presented descriptively in Table 6, with full inferential statistical results reported in Multimedia Appendix 6.

**Table 6.** Exploratory construct-level changes in mean technology acceptance model (TAM) scores by experimental condition in a 3-arm randomized feasibility pilot study of virtual reality therapeutics conducted from December 2024 to February 2025^a^.

TAM construct	Condition 1			Condition 2			Condition 3		
Preintervention, mean (SD)	Postintervention, mean (SD)	Δ Mean	Preintervention, mean (SD)	Postintervention, mean (SD)	Δ Mean	Preintervention, mean (SD)	Postintervention, mean (SD)	Δ Mean
Perceived usefulness	2.80 (0.42)	3.20 (0.63)	+0.40	3.10 (0.21)	3.30 (0.48)	+0.20	2.73 (0.47)	3.32 (0.51)	+0.59
Perceived ease of use	3.00 (0.33)	3.00 (0.47)	+0.00	2.70 (0.63)	3.05 (0.60)	+0.35	2.95 (0.57)	3.50 (0.50)	+0.55
Attitude toward technology	3.10 (0.61)	3.35 (0.53)	+0.25	3.15 (0.34)	3.45 (0.50)	+0.30	2.82 (0.34)	3.36 (0.50)	+0.54
Behavioral intention to use	2.45 (0.55)	2.85 (0.78)	+0.40	2.80 (0.35)	3.20 (0.63)	+0.40	2.50 (0.22)	3.05 (0.52)	+0.55
Overall TAM	2.84 (0.40)	3.10 (0.53)	+0.26	2.94 (0.27)	3.25 (0.49)	+0.31	2.75 (0.29)	3.31 (0.34)	+0.56

a Values represent mean pre- and postintervention TAM construct-level scores and corresponding mean change (Δ mean) within each condition. TAM scores are based on a Likert scale from 1 to 5. These analyses are exploratory and descriptive; inferential statistical results including *P* values and effect sizes are reported in Multimedia Appendix 6. No correction for multiple comparisons was applied to exploratory analyses; results should be interpreted in the context of the overall patterns.

In condition 1 (unguided use), improvements were observed primarily in BI and overall TAM scores, with smaller or negligible changes across PU, PEU, and ATT. Condition 2 (self-directed support) demonstrated modest and relatively consistent improvements across all constructs; however, the magnitude of change was generally limited and did not show clear differentiation across constructs.

In contrast, condition 3 (provider-led support) exhibited the largest and most consistent improvements across all TAM constructs. These findings suggest that more structured, human-directed implementation approaches may be associated with broader and more meaningful gains in technology acceptance, particularly in translating improvements across multiple acceptance domains.

As these analyses were exploratory and no correction for multiple comparisons was applied, findings should be interpreted in the context of overall patterns rather than individual construct-level differences.

#### Engagement and Satisfaction Results

Participants spent an average of 17.00 (SD 5.45) minutes engaging with the VRx intervention. Descriptive engagement and acceptability metrics by experimental condition are summarized in Table 7.

**Table 7.** Engagement and acceptability metrics in the overall sample and by experimental condition in a 3-arm feasibility pilot study of virtual reality therapeutics (VRx) conducted from December 2024 to February 2025.

Experimental condition	Values, n	Mean duration in VRx (SD) (minutes)	Mean task completion rate (SD) (%)	Mean virtual reality engagement (SD) (%)	Mean acceptability score (SD)	Mean acceptability (SD) (%)
Condition 1 unguided use	10	13.80 (6.66)	94.6 (4.5)	56.7 (41.7)	150.0 (28.3)	88.2 (16.6)
Condition 2 self-directed support	10	17.10 (4.82)	93.7 (4.7)	73.3 (34.4)	138.0 (32.4)	81.2 (19.1)
Condition 3 provider-led support	11	19.82 (3.03)	95.8 (3.7)	93.9 (13.5)	149.6 (16.7)	88 (9.8)
Overall sample	31	17.00 (5.45)	94.7 (4.3)	75.3 (34.4)	146.0 (26.1)	85.9 (15.3)

Engagement time varied by condition, with participants in condition 3 (provider-led support) demonstrating the longest duration in VRx (mean 19.82, SD 3.03), followed by condition 2 (self-directed support; mean 17.10, SD 4.82) and condition 1 (unguided use; mean 13.80, SD 6.66). A similar directional pattern was observed across VR engagement and acceptability measures, with condition 3 showing the highest levels overall.

A statistically significant difference across conditions was observed for time spent in VRx (Kruskal-Wallis *χ*^2^_2_=6.7; *P*=.04; *ηH*^2^=0.222). Exploratory post-hoc pairwise comparisons with Bonferroni correction suggested that participants in condition 3 spent significantly more time in VRx compared to those in condition 1 (adjusted *P*=.046) with no statistically significant differences observed between other condition pairs (Table 8). No statistically significant between-group differences were observed for task completion rate, VR engagement percentage, or acceptability scores.

**Table 8.** Post-hoc Wilcoxon pairwise analysis with Bonferroni correction findings suggest that participants in condition 3 spent significantly more time in virtual reality therapeutics (VRx) compared to those in condition 1^a^.

Experimental condition	N1	N2	Wilcoxon statistics	*P* value	*P*.adj
Condition 1 versus condition 2	10	10	34.5	.25	.76
Condition 1 versus condition 3^b^	10	11	20.5	.02	.046
Condition 2 versus condition 3	10	11	33.5	.13	.39

a Analysis is comparing time in VRx within experimental conditions in a 3-arm randomized feasibility pilot study of VRx conducted from December 2024 to February 2025.

b Significant difference (*P*<.05).

These findings suggest that more structured implementation approaches may support deeper engagement with VR interventions, although this effect was primarily reflected in time spent in VRx rather than broader engagement or acceptability measures. Given the exploratory nature of these analyses, the limited sample size, and the reliance on a single pairwise comparison, this finding should be interpreted with caution.

Self-efficacy and satisfaction followed a similar descriptive pattern, with participants in condition 3 reporting the highest scores (self-efficacy: mean 2.97, SD 0.46; satisfaction: mean 3.59, SD 0.44). Across all conditions, mean self-efficacy was 2.88 (SD 0.46), and mean satisfaction was 3.50 (SD 0.45). These measures were not formally compared across conditions and should be interpreted descriptively.

#### Observed Behavioral Results

**Figure 5.**
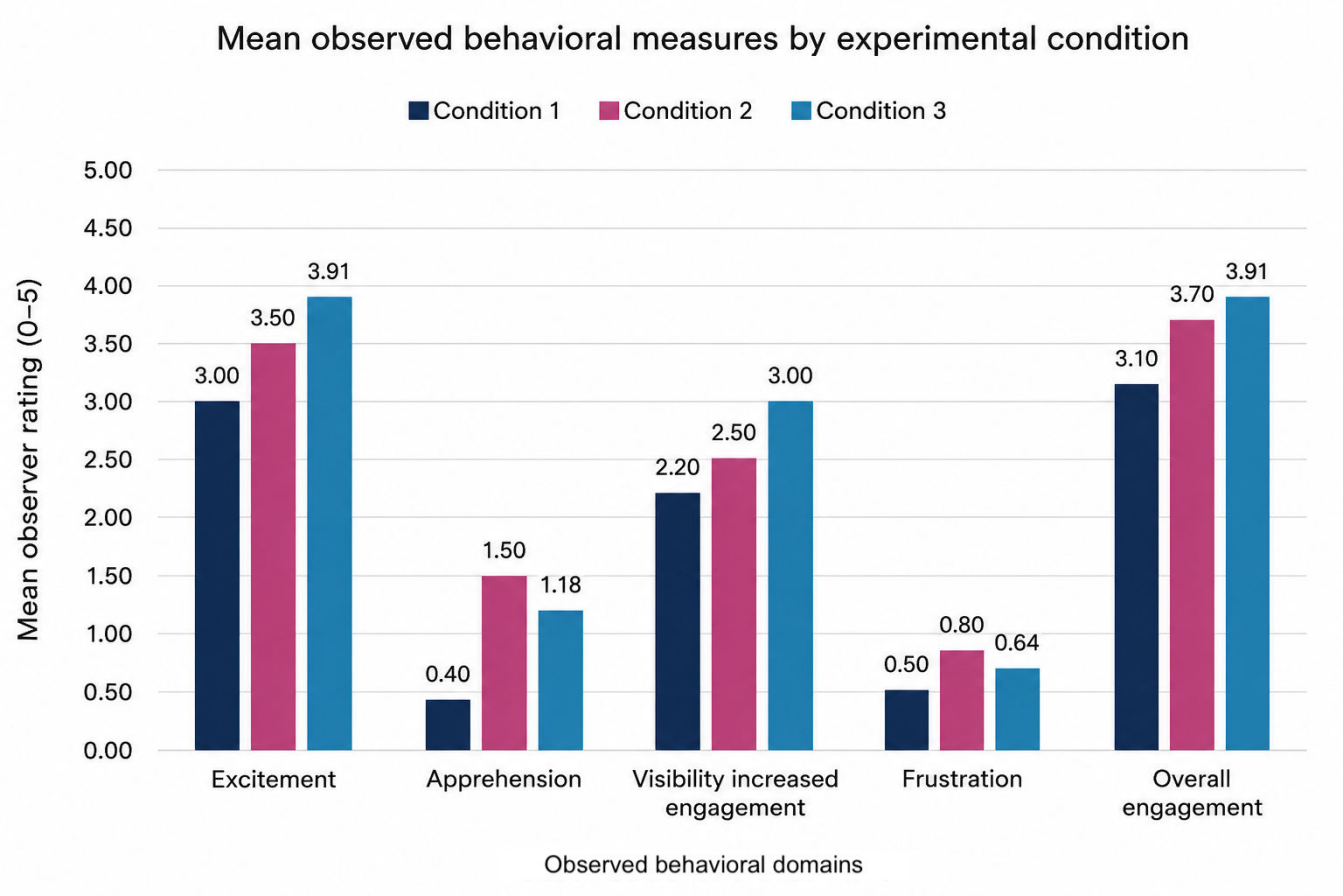
Observed behavioral measures across experimental conditions. Mean observer ratings for excitement, apprehension, visible increased engagement, frustration, and overall engagement are shown for each condition. Higher engagement and excitement, along with lower frustration, were observed among participants receiving provider-led support (condition 3).

Observed behavioral measures varied across experimental conditions (Figure 5). Participants in condition 3 (provider-led support) demonstrated higher levels of excitement and overall engagement, along with lower levels of frustration relative to other conditions. In contrast, participants in condition 2 (self-directed support) exhibited relatively higher levels of apprehension and frustration despite receiving supplemental implementation materials. Participants in condition 1 (unguided use) showed lower excitement and overall engagement in comparison to the other conditions.

These observational patterns suggest that provider-led implementation support may be associated with more positive behavioral responses during VR use, including increased engagement and reduced frustration. Notably, self-directed support materials (condition 2) did not appear to reduce apprehension or frustration, suggesting that the presence of materials alone may be insufficient without structured guidance. As these measures were observational and not statistically tested, findings should be interpreted as descriptive.

### Usability Outcomes

#### Objective Task Performance Metrics Results

Usability metrics by condition are summarized in Table 9. Overall task completion rates were high (mean 94.7%, SD 4.3%), with similar performance across conditions. The lowest mean number of errors was observed in condition 3 (provider-led support; mean 2.09, SD 1.76), while condition 2 (self-directed support) had the fewest help requests (mean 1.60, SD 2.12). Mean usability scores were highest in condition 3 (mean 61.64, SD 12.86; mean 77.4%, SD 16.1%), followed by condition 2 (mean 58.90, SD 8.26; mean 73.8%, SD 10.3%) and condition 1 (mean 56.50, SD 13.55; mean 70.8%, SD 16.9%).

**Table 9.** Usability measures (task completion rate, errors, help requests, and scores) by experimental condition in a 3-arm randomized feasibility pilot study of virtual reality therapeutics conducted from December 2024 to February 2025.

Experimental condition	Mean task completion rate (SD) (%)	Mean number of errors (SD)	Mean help requests (SD)	Mean observed usability score (SD)	Mean usability score (SD) (%)
Condition 1 (n=10)	94.6 (4.5)	3.30 (2.11)	2.22 (1.39)	56.50 (13.55)	70.8 (16.9)
Condition 2 (n=10)	93.7 (4.7)	3.50 (1.96)	1.60 (2.12)	58.90 (8.26)	73.8 (10.3)
Condition 3 (n=11)	95.8 (3.7)	2.09 (1.38)	2.09 (1.76)	61.64 (12.86)	77.4 (16.1)
Overall (N=31)	94.7 (4.3)	2.94 (1.88)	1.97 (1.75)	59.10 (11.63)	74.1 (14.5)

Help requests were examined to characterize usability challenges across conditions. The most common requests were related to navigating the VR system, selecting menu options, and exiting the application. Task 1 generated the highest number of help requests, particularly for powering on the device, setting up boundaries, and using the controllers. These findings suggest that initial onboarding and system navigation represent key usability challenges in VR-based interventions.

#### SUS Results

Overall SUS scores summarized in Table 10 indicated relatively high usability scores across the full sample (mean 83.74, SD 12.52), with condition 3 demonstrating the highest mean score (mean 88.26, SD 10.34), followed by condition 2 (mean 82.50, SD 14.00) and condition 1 (mean 80.00, SD 12.85). No statistically significant between-group differences were observed (Kruskal-Wallis *χ*^2^_2_=2.3; *P*=.31). Given the modified scale used in this study, scores are interpreted as a relative measure of usability across conditions rather than against standard SUS benchmarks.

**Table 10.** System Usability Scale (SUS) scores by experimental condition in a 3-arm randomized feasibility pilot study of virtual reality therapeutics conducted from December 2024 to February 2025^a^.

Experimental condition	Values, n	Mean SUS score (SD)
Condition 1	10	80.00 (12.85)
Condition 2	10	82.50 (14.00)
Condition 3	11	88.26 (10.34)
Overall	31	83.74 (12.52)

a Between-group differences were assessed using Kruskal-Wallis tests. No statistically significant between-group differences were observed for SUS scores (*χ*2_2_=2.3; *P*=.31).

Exploratory item-level findings are presented in Multimedia Appendix 6 and interpreted descriptively. Across conditions, higher ratings were observed for intention to use and PEU, while lower ratings were observed for learnability and need for assistance, suggesting that onboarding and independent use represent key areas for improvement.

### Fidelity Outcomes

Fidelity outcomes by experimental condition are summarized in Table 11, including overall protocol adherence, time-based adherence, and task-based adherence. Mean fidelity scores were highest in condition 3 (mean 70.9, SD 11.4) relative to conditions 1 (mean 58.0, SD 16.5) and 2 (mean 57.5, SD 14.0). Time-based adherence differed across conditions (Kruskal-Wallis *χ*^2^_2_=6.7; *P*=.04), with post-hoc comparisons indicating higher adherence in condition 3 relative to condition 1 (*P*.adj=.046). No other pairwise differences were observed.

**Table 11.** Fidelity outcomes by experimental condition, including overall fidelity score, time and task-based adherence, and task completion prior to engaging in exploratory behaviors in a 3-arm randomized feasibility pilot study of virtual reality therapeutics conducted from December 2024 to February 2025^a^.

Experimental condition	Values, n	Mean fidelity score (SD)	Time-based adherence, mean (SD) (%)	Task-based adherence, mean (SD) (%)	Completed tasks prior to exploratory behavior, n (%)
Condition 1	10	58.0 (16.5)	69.0 (33.3)	94.6 (4.5)	6 (60)
Condition 2	10	57.5 (14.0)	85.5 (24.1)	93.5 (4.8)	6 (60)
Condition 3	11	70.9 (11.4)	99.1 (15.1)	96.0 (3.7)	1 (9)
Overall	31	62.1 (14.0)	85.0 (27.2)	94.7 (4.3)	13 (41.9)

a Between-group differences were assessed using Kruskal-Wallis tests. Time-based adherence differed significantly across conditions (*χ*2_2_=6.7; *P*=.04; *ηH*2=0.222). No significant differences were observed for overall fidelity scores (*χ*2_2_=5.4; *P*=.07) or task-based adherence (*χ*2_2_=1.3; *P*=.52).

Task-based adherence remained high across all conditions (mean 94.7%, SD 4.3%). No statistically significant between-group differences were observed for overall fidelity scores (Kruskal-Wallis *χ*^2^_2_=5.4; *P*=.07) or task-based adherence (Kruskal-Wallis *χ*^2^_2_=1.31; *P*=.52). Although the difference in overall fidelity scores did not reach statistical significance, the *P* value approached the conventional threshold (*P*=.07), suggesting a potential trend toward higher protocol adherence in condition 3 that warrants examination in future adequately powered studies.

Participants in condition 3 were less likely to explore the environment before completing assigned tasks when compared to conditions 1 and 2, suggesting greater adherence to the prescribed task sequence. No significant differences were observed for overall fidelity scores between conditions. Given the exploratory nature of these analyses and small sample size, findings should be interpreted as descriptive.

### Tolerability Outcomes

Participants completed the CSQ-VR prior to and following intervention exposure. Conditions 1 and 2 completed the assessment independently, whereas participants in condition 3 were administered the assessment as part of the VR Fitness Assessment conducted by the mock provider. Tolerability outcomes, measured using the CSQ-VR before and after the intervention, are presented by experimental condition in Table 12.

**Table 12.** Tolerability outcomes by experimental condition measured using the Cybersickness in Virtual Reality Questionnaire (CSQ-VR) administered pre- and postintervention in a 3-arm randomized feasibility pilot study of virtual reality therapeutics conducted from December 2024 to February 2025^a^.

Experimental condition	Values, n	Mean preintervention score (SD)	Mean postintervention score (SD)
Condition 1	10	6.10 (0.32)	6.90 (1.20)
Condition 2	10	6.00 (0.00)	6.20 (0.63)
Condition 3	11	6.73 (1.01)	6.27 (0.90)
Overall CSQ-VR score	31	6.29 (0.69)	6.45 (0.96)

a Scores can range from 6 to 42.

No meaningful increases in cybersickness symptoms were observed following VRx exposure. Mean preintervention symptom scores were low across conditions (mean 6.29, SD 0.69), corresponding to absent to very mild symptoms. Postintervention scores remained similar (mean 6.45, SD 0.96), with no evidence of symptom worsening reported across conditions.

Overall, 74% (23/31) of participants reported no symptoms following the intervention. Among those who reported symptoms, the most common were visual discomfort, dizziness, eye strain, and imbalance or instability. These findings suggest that the VR-based intervention was well tolerated in this sample under the implemented safety and screening procedures.

### Qualitative Insights

**Figure 6.**
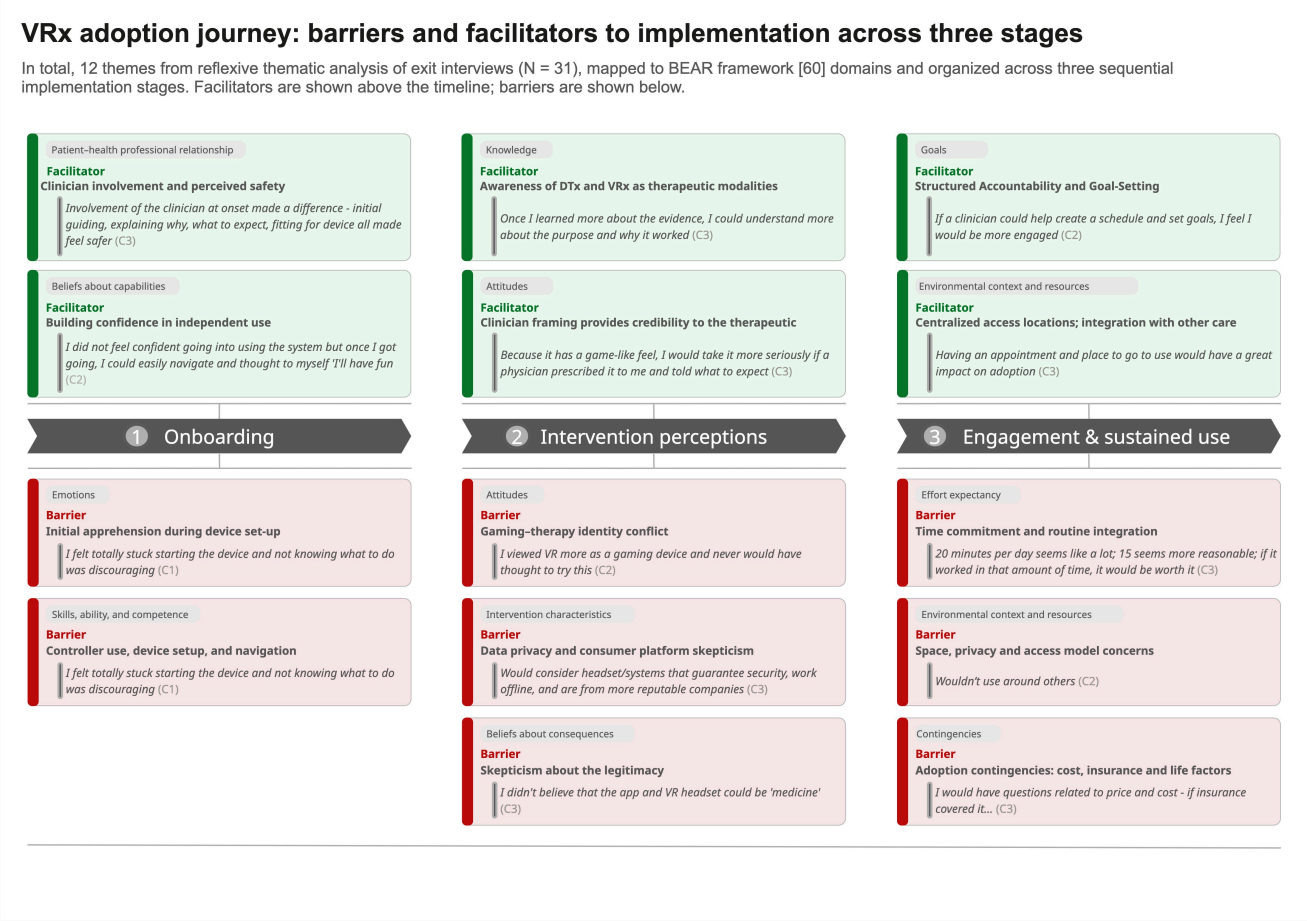
Barriers and facilitators to VRx adoption mapped to BEAR domains across 3 implementation stages. Themes derived from reflexive thematic analysis of exit interviews (N=31). Representative participant quotes are included within each theme card. BEAR: Behavior and Acceptance Framework; C: condition; C1: condition 1 (unguided); C2: condition 2 (self-directed support); C3: condition 3 (provider-supported); DTx: digital therapeutics; VRx: virtual reality therapeutics.

Qualitative insights are presented according to 14 themes derived from reflexive thematic analysis and mapped to BEAR [60] domains to contextualize implementation-relevant barriers and facilitators. Figure 6 presents key themes organized into 3 stages: onboarding, intervention perceptions, and engagement and sustained use, with representative participant quotes from exit interviews. The full BEAR thematic analysis mapping is provided in Multimedia Appendix 6.

Findings highlight several key factors influencing acceptability, usability, and fidelity. First, emotional responses during onboarding shaped early engagement trajectories. Participants across conditions described initial apprehension, particularly around device setup and controller use, that transitioned into enjoyment and relaxation once interaction with the application began. Participants who engaged in a provider-led consultation (condition 3) expressed higher levels of confidence and reduced uncertainty. Participants who received no support (condition 1) were more likely to describe frustration and discouragement during initial setup. Participants emphasized that the first interaction was a critical adoption threshold, noting that “first touch needs to be positive or people will walk away from using it, especially older people” (condition 1). Provider involvement was consistently identified as the most influential factor in shaping perceived safety, confidence, and motivation during onboarding, with participants describing cybersickness screening, device fitting, and expectation-setting as contributing to a greater sense of comfort and legitimacy.

Perceptions of VRx as a therapeutic modality were strongly influenced by how the intervention was framed and presented. Several participants initially viewed VR as a gaming platform, expressing skepticism about its legitimacy as a health intervention. Provider endorsement as well as evidence-based communication materials shifted these perceptions, with participants noting that professional framing made them take the intervention more seriously. A distinct barrier emerged around identity conflict, in which participants struggled to categorize VRx as medicine rather than entertainment, describing engagement with a game-like interface as therapeutic felt like self-deception without clinical framing.

Concerns related to data privacy and the use of consumer technology platforms for health interventions also emerged, with participants expressing preferences for offline functionality, local data storage, and health-dedicated devices. Limited prior awareness of DTx as a therapeutic modality was common across conditions.

Environmental constraints, such as limited home space, concerns about safety, and apprehension about the immersive properties of VR when others are in the home, shaped preferences for more structured settings and centralized locations where patients can go to use VRx outside of the home. For sustained use, participants emphasized the importance of flexible session length, integration into daily routines, and structured accountability and goal setting. Many expressed that without some form of structure, such as goal setting with providers or progress tracking, they would have difficulty maintaining regular engagement. Participants across conditions raised concerns about the affordability of VR hardware and suggested centralized access models such as clinic-based or community “studios” as alternatives for individual device ownership.

Collectively, these findings highlight the central role of provider involvement, structured onboarding, and contextual fit in supporting adoption and sustained use of VR-based DTx.

## Discussion

### Principal Findings

This pilot study evaluated 3 implementation strategies for VR-based DTx, comparing unguided use, self-directed use with support materials, and provider-supported use. A single brief exposure to VRx was associated with statistically significant improvements in acceptability across all conditions. However, postintervention acceptance scores remained within the neutral range, suggesting that while perceptions shifted favorably, a single exposure may be insufficient to produce strong endorsement or readiness for sustained adoption.

Differences across intervention conditions were observed in acceptability, usability, and fidelity-related outcomes. Participants receiving provider-led support demonstrated the most favorable and consistent pattern of favorable outcomes, including longer engagement times, higher protocol adherence, and more positive usability ratings. In contrast, self-directed materials produced more variable engagement and, in some cases, appeared to increase cognitive burden during onboarding. Tolerability was acceptable across all conditions, with no meaningful increases in cybersickness symptoms observed. Qualitative findings reinforced the importance of the initial interaction, clinician endorsement, and structured onboarding in shaping early adoption trajectories.

### Interpretation of Findings

The findings suggest that early user responses to VR-based DTx are shaped not only by exposure to technology itself but also by the implementation strategy through which that exposure is delivered. While all participants demonstrated improved perceptions of VRx following a single exposure, the differences across conditions indicate that acceptability, usability, and fidelity are strongly influenced by the surrounding implementation approach rather than inherent properties of the intervention alone.

Participants in the provider-supported condition demonstrated the most consistent pattern of favorable outcomes, suggesting that structured onboarding and perceived clinical endorsement reduce uncertainty and increase confidence in use. In contrast, reliance on self-directed materials alone appeared insufficient to support early engagement and may introduce cognitive burden during initial use.

Notably, the disconnect observed between improvements in attitudinal acceptance and more modest shifts in behavioral intention suggests that early exposure may not be sufficient to drive adoption. This reinforces established technology acceptance theories, which suggest that early perceptions may shift quickly, while sustained intention and behavior require continued experience and reinforcement [62]. However, in contrast to prior VR studies that emphasize PU as the primary driver of adoption, our findings suggest that ease of use and structured onboarding may play a more prominent role during initial exposure, particularly for first-time users [79].

Findings in this study align closely with insights from other DTx studies where making DTx feel “ordinary” and familiar accelerated patient adoption and acceptance [42]. In this context, provider involvement and structured implementation strategies may serve as mechanisms that help bridge the gap between initial exposure and sustained engagement.

### Role of Provider Support and Human-Centered Implementation

Findings consistently demonstrate that provider involvement plays a critical role in shaping early adoption of VRx. Participants receiving provider-supported onboarding showed greater improvements across acceptability, usability, and adherence outcomes, suggesting that structured human interaction enhances both perceived legitimacy and user confidence. This aligns with broader DTx literature identifying provider endorsement as a critical driver of patient uptake [30,42,80-82]. Our results extend this work by demonstrating that provider involvement influences not only adoption intent but also early usability, engagement, and adherence behaviors during initial exposure.

Provider-led activities, including safety screening, expectation setting, and device setup, appeared to reduce uncertainty and reinforce the therapeutic credibility of VRx. Participants reported greater willingness to engage when the intervention was introduced by a provider, consistent with prior research that provider endorsement enhances perceptions of safety, credibility, and ease of use [28,42,83].

The brief structured nature of the mock consultation suggests that effective implementation may not require a substantial time burden, but rather targeted and well-designed onboarding as part of a familiarization stage. This extends prior research in VR, which found that a familiarization phase before VR exposure reduced surprise effects [84,85]. Future research should evaluate scalable models, including hybrid approaches that combine brief human interaction with digital guidance.

In contrast, reliance on static materials alone was insufficient to support initial engagement. Participants in the self-directed condition relied solely on their own interpretation or ability to digest the information in a timely fashion. The lack of real-time support or personalized guidance may have led to uncertainty or cognitive overload, preventing participants from fully grasping the benefits and ease of using the VR independently. These findings are consistent with other implementation research that found the complexity of the implementation process and the amount of work involved to learn the technologies as barriers for successful adoption [25,86,87]. Future research should evaluate how different types of training delivery (eg, interactive onboarding, human-led instruction, or personalized digital guidance) influence the acceptability of VRx.

Taken together, these findings suggest that structured, human-supported onboarding may reduce early apprehension and improve engagement with VR therapeutics, particularly among first-time users. Provider endorsement remains a powerful lever for uptake, beyond reimbursement or access [88,89]. Notably, the provider interaction observed in this study was brief and structured, suggesting that effective implementation may not require a significant time burden but rather targeted, well-designed interactions. Future research should evaluate scalable models of clinician-supported onboarding, including hybrid approaches that combine brief human interaction with digital guidance.

### Usability Insights

Item-level usability analysis provided granular insights on important barriers to sustained adoption. Participants reported strong intention to use, PEU, and a low perception of system complexity. However, lower confidence, learnability, and a high perceived need for assistance indicate that early usability challenges persist even with implementation support [90].

Compared to other single-exposure VR studies reporting higher usability scores, the slightly lower usability observed in this study may reflect the broader scope of the user experience assessed, from initial device interaction through full session completion, rather than isolated in-app tasks [91]. This more naturalistic approach likely introduced variability reflective of real-world use conditions.

### Fidelity and Adherence

A notable qualitative finding was the distortion of time perception during VRx use. Several participants reported that the 20-minute session felt substantially shorter than its actual duration, suggesting a high degree of immersion and cognitive absorption. This aligns with prior research demonstrating the ability of VR to create immersive, attention-capturing experiences that support therapeutic mechanisms such as distraction, presence, and engagement [6,8,92,93]. The relationship between perceived time, immersion, prescription, and clinical outcomes in VRx should be further examined in future studies.

This finding also has important implications for fidelity, as users may be more likely to complete prescribed session durations when the experience feels shorter and more engaging. Related studies on VR use show that the level of immersion may support positive psychological responses, including increased intrinsic motivation, improved mood, and enhanced self-efficacy, which are known contributors to sustained engagement and adherence in rehabilitation and behavioral interventions over time [7,94,95]. Adherence improvements correlate with better clinical outcomes in many DTx studies [30,82]. Further research is needed to show the longitudinal impact of immersion on fidelity and adherence.

### Preventing Cybersickness

Cybersickness was minimal across participants and did not appear to meaningfully impact engagement or completion of the VRx session. While a small number of participants reported mild symptoms (eg, slight dizziness or discomfort), these were transient and did not lead to early discontinuation [96-98]. The low incidence of cybersickness in this study may also reflect awareness of the side effect from providing the cybersickness questionnaire prior to use. Cybersickness remains an important consideration for broader implementation, particularly in patient populations who may be more susceptible. Future research should evaluate tolerability over repeated exposures and across diverse clinical populations, as well as identify design and implementation strategies that further mitigate risk.

### Scalability and Resource Considerations

The 3 implementation strategies evaluated in this study represent different resource models with important implications for scalability. While provider-supported use produced the strongest outcomes, it introduces additional resource requirements that must be considered in real-world deployment.

The provider interaction in this study was brief (~5 minutes) and delivered by trained personnel, suggesting that effective support may be achievable without substantial burden. In many systems, physicians and psychotherapists act as the main prescribers and gatekeepers, deciding whether DTx are integrated into the treatment pathway [40,43,99]. There is an opportunity for future research to explore alternative delivery models, including nurse-led onboarding, digital-first guidance, or hybrid approaches combining asynchronous support with targeted human interaction.

Participants also identified cost and access as key barriers. Concerns about the affordability of VR hardware and uncertainty around insurance coverage were consistently raised. Suggested solutions included shared-device models, such as clinic-based or community access points, and integration into existing care pathways to reduce both cost and access barriers. Research supports shared-device models, shared digital access, and community or mobile access points as practical, relatively low-cost ways to reduce cost and access barriers, especially when tightly integrated into existing care pathways and supported by appropriate policy and management infrastructure [100-104].

Notably, previous research examining key barriers to DTx adoption showed that improving access to prescriptions and removing cost barriers did not accelerate adoption [34,40,43]. Further investigation found that provider endorsement of DTx significantly impacted patient adoption [43,47]. Adoption of DTx is shaped by intertwined patient, provider, organizational, and regulatory factors [28,42,80,105,106]. Lowering cost or easing prescription alone is insufficient; provider knowledge, trust, workflow fit, and institutional support are central levers for accelerating patient uptake.

While a formal cost analysis was beyond the scope of this study, the relative resource differences across conditions highlight the need for future work evaluating the cost-effectiveness of implementation strategies. Identifying the minimum effective level of provider involvement, as well as opportunities for digital or hybrid onboarding, will be critical for developing scalable models of VRx delivery.

### Provider Needs for Implementation

Although provider training was not a primary focus of this study, trained study personnel acting as “mock providers” were able to deliver a brief, structured onboarding interaction that improved perceived safety, usability, and engagement. These materials were designed to be efficient and minimize time burden, addressing a known barrier for provider adoption: limited time during patient encounters [89]. This suggests that a short “introduce and demonstrate” interaction may be sufficient to support initial adoption without adding substantial time burden to clinical workflows.

Research related to prescribing methods for DTx is limited; methods for prescribing and ensuring patient safety are substantial gaps in how DTx and VRx are delivered today [88,89,107]. There is a substantial opportunity for this research to be expanded on to design simplified tools for patient screening and streamlining the clinical workflow to mitigate adoption barriers experienced by providers. Finally, lightweight screening, dose guidance, and simplified demo devices may further reduce provider friction and support safe, repeatable prescribing.

### Theory Integration

This study integrates complementary frameworks to demonstrate that VRx adoption is shaped by the interaction of user perceptions (TAM), usability experience (SUS), and implementation context (BEAR). While brief exposure improved PU and PEU, these shifts did not translate into strong acceptability, indicating that perception alone is insufficient for adoption [61].

Usability findings further highlight a gap between PEU and user confidence, suggesting that initial support may be required even for well-designed systems [64]. The BEAR contextualizes these findings by demonstrating that implementation strategies, particularly through provider involvement, reduce uncertainty and reinforce legitimacy [60]. This reinforces the role of implementation strategies as active drivers of engagement, not simply delivery mechanisms. These findings suggest that successful VRx deployment requires alignment across perception, usability, and implementation strategy to support both initial engagement and sustained use.

### Implications

Taken together, these findings suggest that implementation strategy is a critical determinant of early adoption of VR-based DTx. By isolating onboarding and provider support components, this study extends prior VRx research that has largely focused on clinical efficacy to address how these interventions are introduced and supported. The results highlight the importance of integrating structured onboarding and provider touchpoints to enhance usability, engagement, and adherence. These findings have practical implications for the design and deployment of scalable VRx solutions in real-world settings.

### Limitations

This study has several limitations that affect the interpretation and generalizability of the findings. First, as a pilot study with approximately 10‐11 participants per condition, it was not powered to detect between-group differences. As a result, both null and statistically significant findings should be interpreted cautiously, and effect size estimates should be considered exploratory rather than definitive.

Second, multiple comparisons were conducted across TAM and SUS constructs, and exploratory subgroup analyses were performed without correction for multiplicity. While consistent with the exploratory nature of pilot studies, this increases the risk of type I error [69,77]. Accordingly, findings should be interpreted based on overall patterns rather than individual comparisons.

Third, the study was conducted in a controlled, nonclinical sample of healthy adults, which may limit generalizability to intended patient populations. Engagement, motivation, and usability may differ in clinical populations, where symptom burden and provider recommendation play a more central role. There was an imbalance in sex distribution across conditions, with a higher proportion of female participants in condition 3, which also demonstrated the most favorable outcomes. Prior research suggests differences in technology acceptance by sex, and this imbalance may represent a potential confounding variable. Simple randomization was used rather than stratified or block randomization. While baseline characteristics including technology comfort level were monitored across conditions, the absence of formal stratification may have contributed to residual imbalance, including a higher proportion of female participants in condition 3 and uneven distribution of technology comfort level across conditions. Allocation concealment from study personnel was also not implemented, and full blinding of participants and study personnel was not feasible, given the visible differences across study arms. Future adequately powered studies should incorporate formal stratification, allocation concealment, and, where feasible, blinded outcome assessment.

Fourth, provider involvement was simulated and may not reflect real-world prescribing, symptom-driven motivation, or clinical care pathways. In clinical populations, the presence of a diagnosed condition and provider recommendation may further influence engagement, adherence, and perceived value of VR therapeutics.

Fifth, differences between the laboratory and community-based study settings may have influenced usability and engagement. Future studies should evaluate these strategies in fully naturalistic, real-world settings to better understand their impact on adoption and sustained use.

Finally, the study evaluated a single, short-term exposure to VRx, precluding assessment of sustained engagement, long-term adherence, and clinical outcomes. Future research should examine longitudinal use in clinical populations and real-world settings to better understand adoption, adherence, and implementation at scale.

### Conclusions

This study provides early evidence that the implementation strategy plays a critical role in shaping the adoption of VR-based DTx. This work is innovative in experimentally isolating implementation components, such as onboarding support and provider involvement, rather than evaluating the therapeutic content alone, which distinguishes it from prior VRx studies that have primarily focused on clinical efficacy. By directly comparing unguided, self-directed, and clinician-supported approaches, this study offers new insight into how implementation design influences early user adoption.

Findings demonstrate that while brief exposure to VRx improved user perceptions across all conditions, structured onboarding and provider involvement produced more consistent improvements in usability, engagement, and adherence. Notably, even brief provider interaction appeared to increase perceived legitimacy, reduce uncertainty, and support early engagement, suggesting a potential pathway for future scalable implementation through low-burden, hybrid onboarding models, warranting validation in larger studies.

These results contribute to the growing field of DTx by emphasizing that successful deployment depends not only on the technology itself but also on how it is introduced and supported. In real-world settings, integrating lightweight provider touchpoints and structured onboarding may improve uptake and sustained use of VRx interventions. Future research should evaluate these strategies in longitudinal and clinical populations to determine their impact on sustained adherence and clinical outcomes.

## Supplementary material

10.2196/90626 Multimedia Appendix 1 Preintervention screening instruments and consent forms.

10.2196/90626 Multimedia Appendix 2 Description and rationale for selection of OpenBrush.

10.2196/90626 Multimedia Appendix 3 Complete implementation package documentation.

10.2196/90626 Multimedia Appendix 4 Pre- and postintervention questionnaires.

10.2196/90626 Multimedia Appendix 5 Structured participant observation guide and checklist.

10.2196/90626 Multimedia Appendix 6 Statistical methods and exploratory analyses.

10.2196/90626 Checklist 1 CONSORT 2010 extension for randomized pilot and feasibility trials.

10.2196/90626 Checklist 2 CONSORT-eHEALTH checklist (V1.6).

## Abbreviations

ATT: attitude toward technology
BEAR: Behavior and Acceptance Framework
BI: Behavioral Intention to Use
CONSORT: Consolidated Standards of Reporting Trials
CONSORT-eHEALTH: Consolidated Standards of Reporting Trials of Electronic and Mobile Health Applications and Online Telehealth
CSQ-VR: Cybersickness in Virtual Reality Questionnaire
DTx: digital therapeutics
IRB: institutional review board
PEU: perceived ease of use
PU: perceived usefulness
REDCap: Research Electronic Data Capture
SUS: System Usability Scale
TAM: technology acceptance model
VR: virtual reality
VRx: virtual reality therapeutics
