# Supplementary material for: Evaluating the Effects of Clinician Prescribing and Implementation Materials on Adoption of Virtual Reality Therapeutics: Randomized Feasibility Pilot Study
Source: JMIR XR Spat Comput. 2026 Jun 30;3:e90626. doi: 10.2196/90626 (PMC13317682; doi:10.2196/90626)
Supplement: Multimedia Appendix 1 [file xr-v3-e90626-s001.pdf]

Eligibility Screener

To determine your initial eligibility for the study, please answer the following key questions. These will help confirm if you meet the basic criteria to proceed before moving on to demographics collection and the health and safety screening.

---

1) Age

What is your current age? ______________________

---

2) Virtual Reality (VR) Experience

How would you describe your experience level with Virtual Reality Headsets?

- ◯ Very Familiar - I own a headset / use a headset frequently / have used a headset more than 10 times
- ◯ Familiar - I am comfortable using a headset / I have used a headset more than 5 times
- ◯ Somewhat familiar - I am not comfortable using a headset / I have used a headset once or twice
- ◯ Not familiar - I have never used a VR headset
- ◯ N/A - I do not know what Virtual Reality is
- ◯ Prefer not to say

---

3) Virtual Reality Comfort

Have you experienced any discomfort (e.g., dizziness, nausea, or headache) when using VR in the past?

- ◯ Yes
- ◯ No
- ◯ I have never used VR in the past
- ◯ Prefer not to say

---

4) General Health

Do you have any medical conditions that might interfere with the safe use of Virtual Reality (VR)?

(e.g,., history of seizure, facial injury precluding safe placement of headset, significant visual impairment that impacts ability to see the VR images, hearing impairment)

- ◯ Yes
- ◯ No
- ◯ Prefer not to answer

---

5) Health History

Do you have a history of psychiatric conditions that might be affected by VR use?

- ◯ Yes
- ◯ No
- ◯ Prefer not to say

---

6) Pregnancy

Are you currently pregnant?

- ◯ Yes
- ◯ No
- ◯ Not Applicable
- ◯ Prefer not to say

---

7) Cognitive or Physical Limitations

Do you have any cognitive or physical conditions that might make it difficult to use VR equipment?

- ◯ Yes
- ◯ No
- ◯ Prefer not to say

Cyber Sickness Susceptibility: The following questions are adapted from the Motion Sickness Susceptibility Questionnaire (MSSQ) and are used to determine any predisposition to cybersickness.

As a child before the age of 12, for each of the following types of transport or entertainment, please inficate how often you Felt Sick or Nauseated.

		Not Applicable - Never Travelled	0 = Never	1 = Rarely	2 = Sometimes	3 = Frequently
8)	Traveling by car	○	○	○	○	○
9)	Traveling by bus or coach	○	○	○	○	○
10)	Traveling by train	○	○	○	○	○
11)	Traveling by plane	○	○	○	○	○
12)	Traveling by small boats	○	○	○	○	○
13)	Traveling by large boats or ships	○	○	○	○	○
14)	Playing on playground equipment (e.g., swings, roundabouts)	○	○	○	○	○

15) Total Childhood MSSQ Score

____

As an adult and based on your eperience over the LAST 10 YEARS (approximately), for each of the following types of transport or entertainment, please inficate how often you Felt Sick or Nauseated.

		Not Applicable - Never Travelled	0 = Never	1 = Rarely	2 = Sometimes	3 = Frequently
16)	Traveling by car	○	○	○	○	○
17)	Traveling by bus or coach	○	○	○	○	○
18)	Traveling by train	○	○	○	○	○
19)	Traveling by plane	○	○	○	○	○
20)	Traveling by small boats	○	○	○	○	○
21)	Traveling by large boats or ships	○	○	○	○	○
22)	Playing on playground equipment (e.g., swings, roundabouts)	○	○	○	○	○

23) Total MSSQ Adult Score

____

24) Final MSSQ Score

____

Demographics

To better understand our participants' backgrounds, we request some basic demographic information. Your responses are confidential and will be used solely for research purposes. Participation is voluntary; you may opt out of any question you prefer not to answer by selecting "prefer not to say".

Demographic Collection

1) First Name

____

2) Last Name

____

3) Birthdate

Please provide your birthdate in the format MM/DD/YYYY.

____

4) Email Address

Please provide your email address. This will remain confidential and will only be used for the study team to contact you regarding scheduling your study participation slot.

____

5) Email Consent

The study team would like to message you by email, however you may say "no" to receiving these messages and still participate in this study. If you say "yes", messages may contain personal information about you such as your name and may be sent or received by the study team's personal electronic devices or in a method that is not able to be encrypted (protected) and there is the risk your information could be shared beyond you and the study team. This information may include information such as reminders and notifications to contact the study team.

- ◯ Yes, I consent to the study team utilizing the following email address to send communications
- ◯ No, I do not consent to receive un-protected communication from the study team.

6) Gender

What is your gender?

- ◯ Male
- ◯ Female
- ◯ Non-binary
- ◯ Other
- ◯ Prefer not to say

7) Race (Select all that apply)

How would you describe your race?

- ☐ American Indian or Alaska Native
- ☐ Asian
- ☐ Black or African American
- ☐ Native Hawaiian or other Pacific Islander
- ☐ White
- ☐ Other
- ☐ Prefer not to say

8) Ethnicity

How would you describe your ethnicity?

- ◯ Hispanic or Latino or Spanish Origin
- ◯ Not Hispanic or Latino or Spanish Origin
- ◯ Prefer not to say

9) Education Level

What is the highest level of education you have completed?

- ◯ Less than high school diploma
- ◯ High school diploma or GED
- ◯ Some college, no degree
- ◯ Associate's degree
- ◯ Bachelor's degree
- ◯ Master's degree
- ◯ Doctoral or professional degree
- ◯ Prefer not to say

10) Employment Status

What is your current employment status?

- ◯ Employed full-time
- ◯ Employed part-time
- ◯ Self-employed
- ◯ Student
- ◯ Unemployed, looking for work
- ◯ Unemployed, not looking for work
- ◯ Retired
- ◯ Prefer not to say

Technology Acceptance Survey

11) Technology Comfort Level

How would you rate your overall comfort level with using everyday technology (e.g. mobile phones, computers)?

- ◯ Very Comfortable
- ◯ Comfortable
- ◯ Uncomfortable
- ◯ Very Uncomfortable

12) Advanced Technology Comfort Level

How would you rate your overall comfort level with using more advanced technology (e.g., virtual or augmented reality, robotics, 3D printing)?

- ◯ Very Comfortable
- ◯ Comfortable
- ◯ Uncomfortable
- ◯ Very Uncomfortable

13) Technology Adoption Habits

How would you describe your technology adoption habits?

- ◯ I am an early adopter and enjoy trying new technologies as soon as they are available.
- ◯ I adopt new technologies once they are proven to be reliable and widely used.
- ◯ I am cautious and only adopt new technologies when necessary.
- ◯ I prefer to stick with familiar technologies and rarely adopt new ones.

For the following questions, please indicate how much you agree with the statement based on a scale of 1 through 4, where 1 indicates that you strongly disagree and 4 indicates that you strongly agree.

14) I believe that virtual reality is useful for managing health-related symptoms.

◯ 1. Strongly Disagree
◯ 2. Disagree ◯ 3. Agree
◯ 4. Strongly Agree

15) Using virtual reality can improve my ability to manage health-related symptoms.

◯ 1. Strongly Disagree
◯ 2. Disagree ◯ 3. Agree
◯ 4. Strongly Agree

16) I expect this VR system to be easy to use.

◯ 1. Strongly Disagree
◯ 2. Disagree ◯ 3. Agree
◯ 4. Strongly Agree

17) Learning to use virtual reality will be straightforward.

◯ 1. Strongly Disagree
◯ 2. Disagree ◯ 3. Agree
◯ 4. Strongly Agree

18) I feel positive about using virtual reality for health interventions.

◯ 1. Strongly Disagree
◯ 2. Disagree ◯ 3. Agree
◯ 4. Strongly Agree

19) I think using virtual reality technology for health purposes is a good idea.

◯ 1. Strongly Disagree
◯ 2. Disagree ◯ 3. Agree
◯ 4. Strongly Agree

20) Outside of participating in this study, I plan to use virtual reality rechnology for health-related purposes in the future.

◯ 1. Strongly Disagree
◯ 2. Disagree ◯ 3. Agree
◯ 4. Strongly Agree

21) I am willing to adopt virtual reality technology as part of my health management routine.

◯ 1. Strongly Disagree
◯ 2. Disagree ◯ 3. Agree
◯ 4. Strongly Agree

The following section includes your consent to participate in the study "Exploring the Effects of Prescribing and Implementation Techniques on the Adoption of Virtual Reality Therapeutics".

Study Participation Consent Form

University of North Carolina at Chapel Hill

IRB Study #: 24-2894

Study Title: Exploring the Effects of Prescribing and Implementation Techniques on the Adoption of Virtual Reality Therapeutics

Principal Investigator: Ashlyn Zebrowski, MS

The purpose of this research study is to test the impact of adoption techniques designed for use with virtual reality therapeutics (VRx) to manage and treat symptoms outside of the clinical setting. In the laboratory setting, this study will measure the impact on acceptance, usability, and fidelity from providing VRx assistive and training materials and from simulating a "mock physician consultation" to provide a "mock anxiety diagnosis and prescription" for VRx.

You are being asked to take part in a research study because you are a healthy individual between the ages of 18 and 45 with self-reported limited prior experience with VR, defined as either having "never used VR" or "used VR only once or twice." You are in good general health and do not have any conditions that could interfere with safe VR use (e.g., epilepsy, severe motion sickness, psychiatric conditions). You are not prone to motion sickness, do not have any cognitive impairments or physical limitations that would prevent effective use, and are not currently pregnant. You must be able to self-rate your level of comfort with technology and be able to give informed consent.

Being in a research study is completely voluntary. You can choose not to be in this research study. You can also say yes now and change your mind later. Deciding not to be in the research study, now or later, will not influence your grades, employment evaluations, promotions, or any other benefits related to your university status or position at UNC Health.

If you agree to take part in this research, you will be asked to complete the following next steps:

- Sign this consent form
- Schedule an appointment to participate in the study using the following link: VR Research Study Appointment
- Attend your appointment to participate in the research

When you arrive at your appointment, you will be screened for any symptoms that might put you at risk for cybersickness. You will be provided with instructions for tasks to complete using a VR headset and software application. Depending on your assigned condition, you may receive additional training or assistive materials.

You also may be assigned to participate in a "mock physician consultation" involving a simulated discussion about a "mock anxiety diagnosis and prescription" for VRx. Prior to the "mock physician consultation", you will be briefed to ensure you fully understand that the "mock anxiety diagnosis and prescription" that will be discussed do not have any impact on your health status. Please note that this "mock anxiety diagnosis and prescription" is not based on your medical history but is designed to simulate a provider-patient interaction for the purposes of this study.

After completing the VR tasks, you will be asked to complete a follow-up questionnaire and participate in a brief interview to discuss your experience. Finally, you will be debriefed to ensure full understanding of the study's purpose, the simulated nature of the interactions, and to address any questions or concerns you may have.

Your participation in this study will take about 1 hour. We expect that up to 45 people will take part in this research study.

For the questionnaires, you can choose not to answer any question you do not wish to answer. You can also choose to stop taking the survey at any time. You must be at least 18 years old to participate. If you are younger than 18 years old, please stop now.

The possible risks to you in taking part in this research are:

- Physical Discomfort During the Study: Some discomfort may occur while using the VR headset, such as eye strain, fatigue, mild headaches, or neck strain.

- Psychological Discomfort: You may feel discomfort during the questionnaire or interview when discussing your personal experiences or opinions related to VRx or technology acceptance.
- Cybersickness: Participants may experience symptoms of cybersickness, such as nausea, dizziness, disorientation, or headaches while using the VR headset. Participants may discontinue use at any time during the experiment.
- Potential Impact on Confidentiality: There is a potential risk of loss of confidentiality of your responses. Although every effort will be made to maintain the privacy of your data, there is a minimal risk that someone outside the study could identify that you participated in this research.
- Potential Impact on Professional Standing or Reputation: Although unlikely, there is a chance that someone else could learn about your involvement in the study or associate your responses with you. There may be concerns that sharing your experiences and opinions could impact your professional reputation if confidentiality is breached.

These risks are mitigated through careful monitoring by the study team, cybersickness pre-screening to rule out higher risks of occurrence, and by providing the option to withdraw from the study at any time without penalty. All data collected will be de-identified and securely stored to protect participant confidentiality. To protect your identity as a research subject, all research data will be stored without your name, and any personally identifiable information will be removed. The researcher(s) will not share your information with anyone. Additionally, in any publications resulting from this research, your name or other private information will not be disclosed.

The study team would like to message you by email, however you may say "no" to receiving these messages and still participate in this study. If you say "yes", messages may contain personal information about you and may be sent or received by the study team's personal electronic devices or in a method that is not able to be encrypted (protected) and there is the risk your information could be shared beyond you and the study team. This information may include information such as reminders and notifications to contact the study team.

If you wish to stop receiving unprotected communication from the study team, please notify the team using the study contact information below. After the study is complete and all research activities finished, or you withdraw from the study or request to stop receiving unprotected communication, you will no longer receive un-encrypted (un-protected) messages specific to this study.

If you have any questions about this research, please contact the Investigator Ashlyn Zebrowski by calling +1 984 328-2900 or emailing zebrows4@email.unc.edu. If you have questions or concerns about your rights as a research subject, you may contact the UNC Institutional Review Board at 919-966-3113 or by email to IRB_subjects@unc.edu.

---

22) Consent Signature

____

Post-Study Questionnaire

Please complete this survey after you have participated in the research study.

First Name ______________________

Last Name ______________________

Birthdate ______________________

Please provide your birthdate in the format MM/DD/YYYY

Did you experience any of the following symptoms of cybersickness while using the Virtual Reality system? (Check all that apply)

- ☐ I did not experience any symptoms
- ☐ Nausea
- ☐ Dizziness
- ☐ Headache
- ☐ Eye strain or discomfort
- ☐ Blurred vision
- ☐ Fatigue
- ☐ Other

What symptoms did you experience? ______________________

How would you rate the severity of your symptoms?

- ◯ Very Mild
- ◯ Mild
- ◯ Moderate
- ◯ Severe

I received training and assistive materials during my VR experience. ◯ Yes ◯ No

I participated in a mock physician consultation. ◯ Yes ◯ No

Technology Acceptance Questionnaire

For the following questions, please indicate how much you agree with the statement based on a scale of 1 through 4, where 1 indicates that you strongly disagree and 4 indicates that you strongly agree.

I believe that using virtual reality is useful for managing health-related symptoms.	◯ 1. Strongly disagree ◯ 2. Disagree ◯ 3. Agree ◯ 4. Strongly Agree
Using virtual reality can improve my ability to manage health-related symptoms.	◯ 1. Strongly disagree ◯ 2. Disagree ◯ 3. Agree ◯ 4. Strongly Agree
I found this VR system easy to use.	◯ 1. Strongly disagree ◯ 2. Disagree ◯ 3. Agree ◯ 4. Strongly Agree
It was easy to learn how to operate the VR system.	◯ 1. Strongly disagree ◯ 2. Disagree ◯ 3. Agree ◯ 4. Strongly Agree
I feel positive about using virtual reality for health interventions.	◯ 1. Strongly disagree ◯ 2. Disagree ◯ 3. Agree ◯ 4. Strongly Agree
I think using virtual reality technology for health purposes is a good idea.	◯ 1. Strongly disagree ◯ 2. Disagree ◯ 3. Agree ◯ 4. Strongly Agree
I am likely to use VR technology for health-related purposes in the future.	◯ 1. Strongly disagree ◯ 2. Disagree ◯ 3. Agree ◯ 4. Strongly Agree
I am willing to adopt virtual reality rechnology as part of my health management routine.	◯ 1. Strongly disagree ◯ 2. Disagree ◯ 3. Agree ◯ 4. Strongly Agree
I would recommend VR technology to others for health management.	◯ 1. Strongly disagree ◯ 2. Disagree ◯ 3. Agree ◯ 4. Strongly Agree
I am satisfied with my overall experience using the VR system.	◯ 1. Strongly disagree ◯ 2. Disagree ◯ 3. Agree ◯ 4. Strongly Agree

My expectations for using the VR system were met.	◯ 1. Strongly disagree<br>◯ 2. Disagree<br>◯ 3. Agree<br>◯ 4. Strongly Agree

**Experience and Usability Questionnaire**<br><br>**The following questions are designed to better understand the usability of the virtual reality solution, including how easy it was to use, how well it met your needs, and any challenges you may have encounteered during your experiene.**	
I think I would use virtual reality more frequently.	◯ 1. Strongly disagree<br>◯ 2. Disagree<br>◯ 3. Agree<br>◯ 4. Strongly Agree
I found the VR system unneccarily complex.	◯ 1. Strongly disagree<br>◯ 2. Disagree ◯ 3. Agree<br>◯ 4. Strongly Agree
I thought the VR system was easy to use.	◯ 1. Strongly disagree<br>◯ 2. Disagree ◯ 3. Agree<br>◯ 4. Strongly Agree
I would need technical support to use this VR system.	◯ 1. Strongly disagree<br>◯ 2. Disagree ◯ 3. Agree<br>◯ 4. Strongly Agree
I received enough technical support to use this system effectively.	◯ 1. Strongly disagree<br>◯ 2. Disagree ◯ 3. Agree<br>◯ 4. Strongly Agree
I imagine most people would learn to use the VR system quickly.	◯ 1. Strongly disagree<br>◯ 2. Disagree ◯ 3. Agree<br>◯ 4. Strongly Agree
I found the VR system cumbersome to use.	◯ 1. Strongly disagree<br>◯ 2. Disagree ◯ 3. Agree<br>◯ 4. Strongly Agree
I felt confident using the VR system.	◯ 1. Strongly disagree<br>◯ 2. Disagree ◯ 3. Agree<br>◯ 4. Strongly Agree
I needed to learn a lot of things before I could use the VR system.	◯ 1. Strongly disagree<br>◯ 2. Disagree ◯ 3. Agree<br>◯ 4. Strongly Agree

Usability of System Enhancements	
I received enough training and assistive materials to use the VR system effectively.	◯ 1. Strongly disagree<br>◯ 2. Disagree ◯ 3. Agree<br>◯ 4. Strongly Agree
Having training and assistive materials created a supportive environment that made it easier for me to learn how to use the VR system.	◯ 1. Strongly disagree<br>◯ 2. Disagree ◯ 3. Agree<br>◯ 4. Strongly Agree
I felt that the assistive materials were not effective in helping me use the VR system effectively.	◯ 1. Strongly disagree<br>◯ 2. Disagree ◯ 3. Agree<br>◯ 4. Strongly Agree

Physician Consultation Impact	
The mock physician consultation helped me better understand how the use of virtual reality would impact my health-related needs.	◯ 1. Strongly disagree<br>◯ 2. Disagree ◯ 3. Agree<br>◯ 4. Strongly Agree
The mock physician consultation and presence of a physician made me feel more comfortable and supported when using the VR system.	◯ 1. Strongly disagree<br>◯ 2. Disagree ◯ 3. Agree<br>◯ 4. Strongly Agree
The mock consultation addressed any concerns I had about using the VR system.	◯ 1. Strongly disagree<br>◯ 2. Disagree ◯ 3. Agree<br>◯ 4. Strongly Agree
Having a physician prescribe or recommend virtual reality would make me more likely to use it for managing health-related symptoms.	◯ 1. Strongly disagree<br>◯ 2. Disagree ◯ 3. Agree<br>◯ 4. Strongly Agree
The information provided during the mock physician consultation impacted my perspective of using the VR system.	◯ 1. Strongly disagree<br>◯ 2. Disagree ◯ 3. Agree<br>◯ 4. Strongly Agree
