# Supplementary material for: Evaluating the Effects of Clinician Prescribing and Implementation Materials on Adoption of Virtual Reality Therapeutics: Randomized Feasibility Pilot Study
Source: JMIR XR Spat Comput. 2026 Jun 30;3:e90626. doi: 10.2196/90626 (PMC13317682; doi:10.2196/90626)
Supplement: Multimedia Appendix 2 [file xr-v3-e90626-s002.pdf]

Description and rationale for selection of OpenBrush

1. Introduction

Art therapy promotes emotional release and psychological recovery through artistic creation and expression and has been clinically proven effective for various mental health problems, including anxiety [1]. VR-based art therapy has been proven to be effective in reducing anxiety, as it combines creative expression with immersive environments that help users detach from stressors in the real world [2,3]. A study on VR-based art therapy demonstrated that using VR head-mounted display (HMD) devices and interactive digital art applications significantly reduced anxiety and depression and improved emotional regulation [1]. The findings showed statistically significant improvements in subjective measures ($P<.05$, $P<.01$) as well as objective physiological responses, including a decrease in heart rate (M=10–15 beats per minute) and a reduction in skin conductance response, indicating lower stress levels [1].

VR art therapy fosters emotional exploration through artistic expression in an immersive, controlled setting, effectively reducing anxiety [4]. A prior study conducted a randomized controlled trial specifically using Tilt Brush, a VR painting app, to test the utility of VR art therapy and the therapeutic benefits as compared to freeform drawing in real life [5]. This study found that the therapeutic benefits of VR art therapy were comparable to real life drawing, and that participants (n=20) that were randomized to use VR art therapy experienced a significant reduction in anxiety ($P<.01$). Notably, while VR art therapy produced comparable anxiety reductions to real-life drawing, its immersive nature may offer distinct advantages, such as enhanced engagement and reduced external distractions [3,5,6]. The original Tilt Brush application is now available as a free, open-source platform called OpenBrush [7].

2. About OpenBrush

OpenBrush is a free, community-driven VR painting application that enables users to create three-dimensional artwork in an immersive environment. Originating from Google's open-sourced Tilt Brush, OpenBrush has expanded upon its predecessor by incorporating new features and enhancements contributed by the developer community. It is available on multiple platforms, including Steam, Meta Quest, and SideQuest, making it compatible with many VR headsets and accessible to a broad audience [7,8].

2.1 Therapeutic Application

The therapeutic potential of VR art applications like OpenBrush has been explored in various studies [5,6,9]. Research indicates that engaging in creative activities within VR can lead to significant reductions in anxiety levels [4]. For instance, a study comparing traditional drawing to VR-based drawing using OpenBrush found that participants experienced similar levels of flow, a state of deep immersion, and a notable decrease in state anxiety following VR sessions [5]. Additionally, art therapists have begun integrating VR tools into clinical practice, utilizing applications like OpenBrush to facilitate creative expression and psychomotor therapy [10].

2.2 Application Suitability

OpenBrush is a suitable application for this VR intervention for several reasons. First, prior research has explored how art therapists learned and applied the software and delivery modality (VR) in clinical settings, including specialist mental health care and private practice [6]. These findings support its real-world use, offering foundational insights into clinician adoption and learnability, thereby strengthening the practical relevance of this study's results. Second,

evidence in prior studies confirms that using OpenBrush as a therapy modality reduces anxiety, with previous studies finding statistically significant reductions in anxiety after a single session where participants (n=20) were instructed to simply draw something abstract or concrete [5]. Third, prior work has utilized adapted versions of OpenBrush to explore the use of VR art therapy in managing negative emotions [11], facilitating emotional expression [12], and enhancing parent-child communication [12]. The diverse applications of OpenBrush highlight its adaptability in research, allowing customization to suit various therapeutic and experimental objectives.

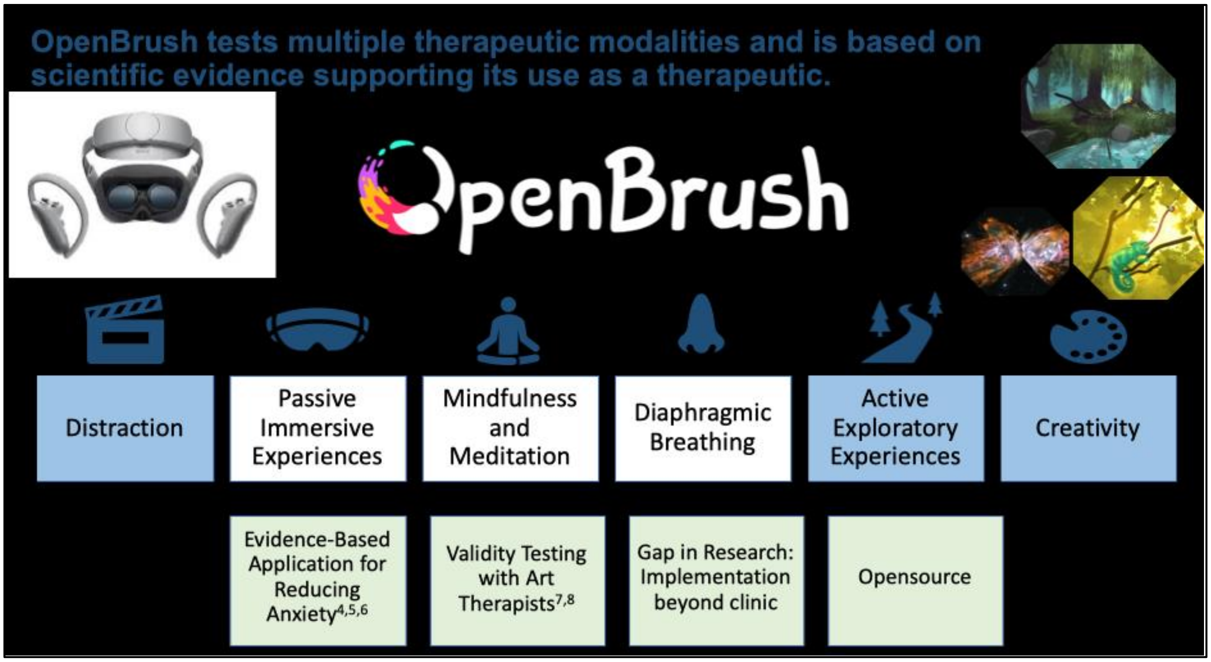

Figure S1. OpenBrush incorporates multiple treatment modalities in a single application while providing a versatile experience and controlling for confounding variables.

The versatility of OpenBrush's features makes it an ideal application for this type of study. The application allows for a self-guided interactive experience, allowing users a wide range of options and choices to create their own environment. Users can choose their own experience, providing an opportunity to gage engagement levels based on observing how users interact with the application. OpenBrush can be used seated or standing. OpenBrush can be put into a beginner mode, reducing the number of options seen on the screen to mitigate an overwhelming initial experience but still allow for enough choices to measure usability and fidelity of use. It has a step-by-step tutorial that can be turned on or off to test the impact of in-app tutorials as part of the implementation assistance.

Additionally, OpenBrush's intuitive design minimizes learning barriers, ensuring consistent participant experiences across experimental conditions. Even users with minimal VR experience can navigate the platform efficiently, addressing concerns about time constraints and the ability to measure outcomes in a single-session intervention. OpenBrush's customization options, such as color selection and brush styles, offer a balance of creativity and control, enabling participants to engage in therapeutic activities without feeling overwhelmed or limited by the amount of intervention time prescribed. Given its proven therapeutic benefits, ease of use, and flexibility of design, OpenBrush serves as a powerful tool for advancing VRx research.

2.3 Application Features and Functionality

OpenBrush offers a range of features that contribute to its utility in therapeutic settings:

a. **Diverse Brush Selection**: Users can choose from an extensive palette of brushes, including options that simulate stars, light, and fire, allowing for varied and personalized artistic expression.
b. **3D Painting Environment:** The application enables painting in three-dimensional space, providing a unique canvas that surrounds the user and encourages exploration and creativity. It creates an interactive, fully immersive environment.
c. **User-Friendly Interface:** Designed with accessibility in mind, OpenBrush features an intuitive interface that accommodates users with varying levels of technical proficiency, ensuring a seamless creative experience and allowing the testing of usability through sequencing tasks from simple to advanced.
d. **Advanced Tools:** The application includes advanced tools which allow users to explore and engage creatively, such as changing their environment background and teleporting within the environment.

These features collectively make OpenBrush a valuable asset for this study, not only due to its emerging scientific support as a therapeutic tool and validation by art therapists, but also for its suitability in testing implementation strategies, techniques, and delivery methods in real-world settings.

2.4 Study Relevance

OpenBrush serves as a pivotal tool for examining the impact of immersive art creation on patient well-being. Its capacity to engage users in creative processes within a virtual environment aligns with therapeutic goals aimed at reducing anxiety and enhancing mood. By providing an interactive and immersive platform for artistic expression, OpenBrush offers participants an opportunity to experience the therapeutic benefits associated with creative engagement in a novel and technologically advanced medium.

References

1. Jing Z, Bakhir NM. Digital Art and Mental Health: A Study on Art Therapy in Virtual Reality Environments. Zenodo; 2024. doi: 10.5281/zenodo.13730098

2. Jingili N, Oyelere SS, Nyström MBT, Anyshchenko L. A systematic review on the efficacy of virtual reality and gamification interventions for managing anxiety and depression. Front Digit Health Frontiers; 2023 Nov 7;5. doi: 10.3389/fdgth.2023.1239435

3. Hadjipanayi C, Banakou D, Michael-Grigoriou D. Art as therapy in virtual reality: A scoping review. Front Virtual Real Frontiers; 2023 Feb 9;4. doi: 10.3389/frvir.2023.1065863

4. Kim H, Kim DJ, Kim S, Chung WH, Park K-A, Kim JDK, Kim D, Kim MJ, Kim K, Jeon HJ. Effect of Virtual Reality on Stress Reduction and Change of Physiological Parameters Including Heart Rate Variability in People With High Stress: An Open Randomized Crossover Trial. Front Psychiatry Frontiers; 2021 Aug 10;12. doi: 10.3389/fpsyt.2021.614539

5. Tan J, Kannis-Dymand L, Jones C. Examining the potential of VR program Tilt Brush in reducing anxiety. Virtual Reality 2022 Nov 10;1. PMID:36405877

6. Haeyen S, Jans N, Heijman J. The use of VR tilt brush in art and psychomotor therapy: An innovative perspective. The Arts in Psychotherapy 2021 Nov 1;76:101855. doi: 10.1016/j.aip.2021.101855

7. Brush O. Open Brush. Available from: https://openbrush.app/ [accessed Nov 1, 2024]

8. Home | Open Brush Docs. 2024. Available from: https://docs.openbrush.app [accessed Mar 31, 2025]

9. Schaaf A. Tilt Brush: The Utilization of a Virtual Reality Intervention for Evaluating Self-Reported Anxiety, Depression, & Stress. University of Cinncinati ProQuest Dissertations & Theses 2019; Available from: https://www.proquest.com/openview/1cc22496a2abbd940d8caeda25b7e066/1?pq-origsite=gscholar&cbl=51922&diss=y [accessed Feb 26, 2025]

10. Ying-Chun L, Chwen-Liang C. The Application of Virtual Reality Technology in Art Therapy: A Case of Tilt Brush. 2018 1st IEEE International Conference on Knowledge Innovation and Invention (ICKII) Jeju, Korea (South): IEEE; 2018. doi: 10.1109/ICKII.2018.8569081

11. Wagner N, Kiesewetter A, Reicherts L, Wozniak P, Schoening J, Rogers Y, Niess J. MoodShaper: A Virtual Reality Experience to Support Managing Negative Emotions. Proceedings of the 2024 ACM Designing Interactive Systems Conference IT University of Copenhagen, Denmark: ACM; 2024. doi: https://doi.org/10.1145/3643834.3661570

12. Stefanidi E, Wagener N, Augsten D, Augsten A, Reicherts L, Woźniak PW, Schöning J, Rogers Y, Niess J. TeenWorlds: Supporting Emotional Expression for Teenagers with their Parents and Peers through a Collaborative VR Experience. 30th ACM Symposium on Virtual Reality Software and Technology Trier Germany: ACM; 2024. p. 1–14. doi: 10.1145/3641825.3687754
