# Supplementary material for: Evaluating the Effects of Clinician Prescribing and Implementation Materials on Adoption of Virtual Reality Therapeutics: Randomized Feasibility Pilot Study
Source: JMIR XR Spat Comput. 2026 Jun 30;3:e90626. doi: 10.2196/90626 (PMC13317682; doi:10.2196/90626)
Supplement: Multimedia Appendix 3 [file xr-v3-e90626-s003.pdf]

Implementation Package: Equipment, Materials, Protocol, Clinician Script

This appendix describes the implementation package used in this study, including the equipment provided to participants by experimental condition, the implementation tools and techniques mapped to BEAR framework [1] barrier categories, and the standardized task protocol completed by all participants.

Table S1. Equipment provided to participants by experimental condition, including VR hardware, software configuration, and accessories.

Category	Condition 1	Condition 2 and 3
VR System	Oculus Quest 2, 64gb	Oculus Quest 2, 256gb
	Controllers	Controllers
Software	OpenBrush Application Pre-Loaded	OpenBrush Application Pre-Loaded and Menu Pre-configured to only show OpenBrush Application
Accessories	1 Sweat-Proof Leather Face Pad with Waterproof Surface. 0.3” thick face pad.	KIWI Comfort Face Cushion: Upgraded Fitness Facial Interface with Glasses Spacer and Lens Protector, Air-Circulation Design. 0.63" thickened face pad
		KIWI Comfort Battery Head Strap
		Mytrix Carrying Case for Meta/Oculus Quest 2 VR Headset and Controllers

Note: Conditions 2 and 3 received enhanced equipment to support the implementation package. Condition 1 received standard equipment only.

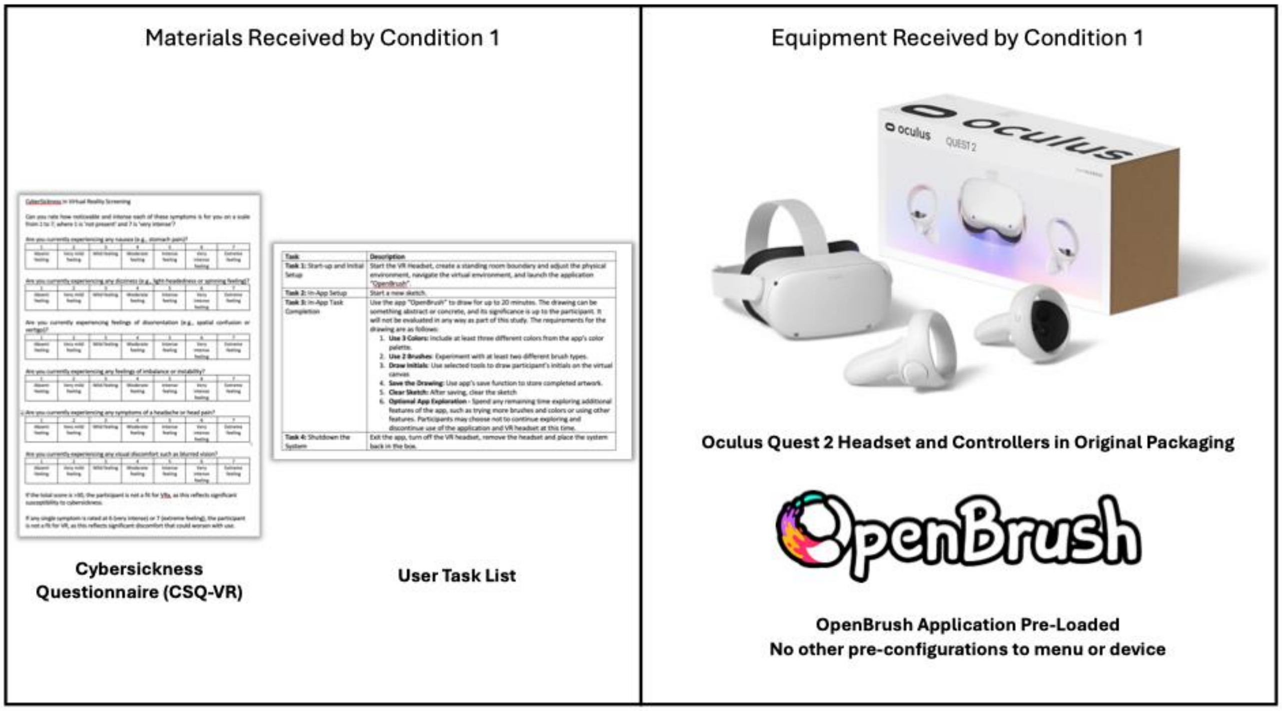

Figure S1. Materials and equipment received by participants in Condition 1 (No Support), including the Oculus Quest 2 headset, controllers, and standard face pad.

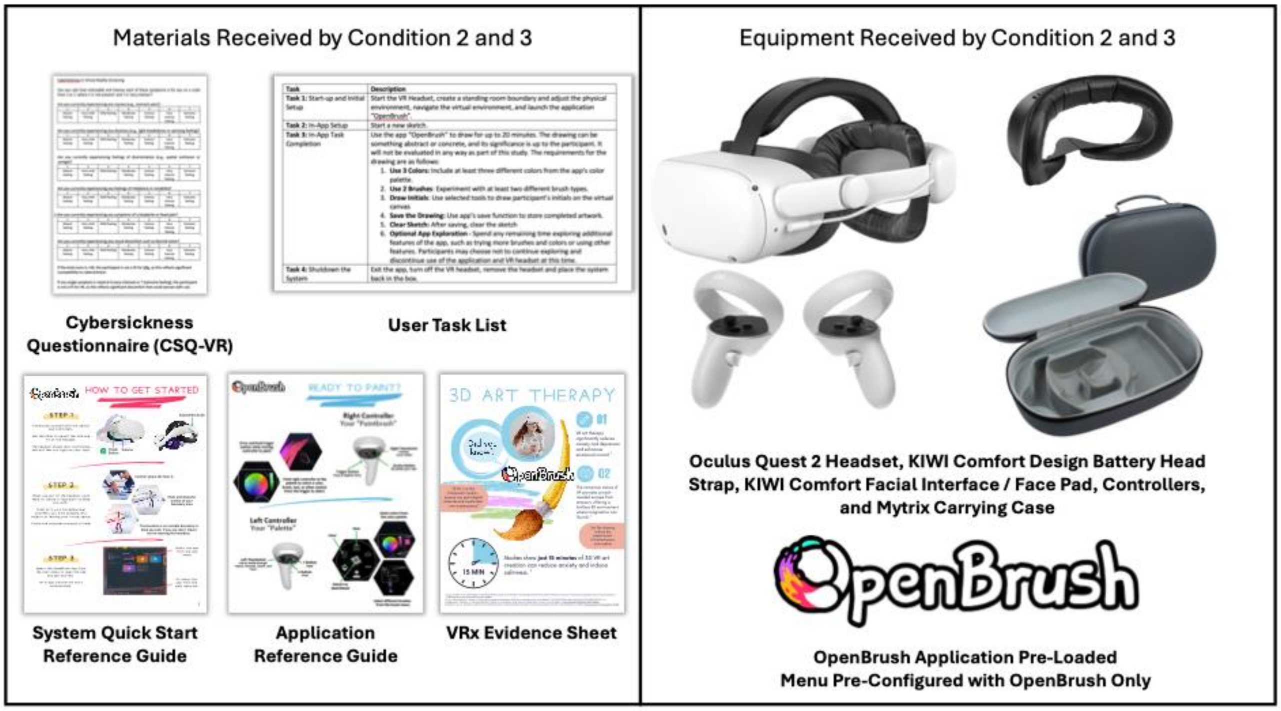

Figure S2. Materials and equipment received by participants in Conditions 2 (Self-Directed Support) and 3 (Provider-Led Support), including enhanced accessories and the implementation package materials.

Table S2. Implementation tools and techniques included in the VR therapeutics implementation package, mapped to corresponding BEAR framework barrier categories.

**Barrier Category**	**Implementation Tool or Technique**
Knowledge	Clinical Evidence Infographic
Skills, Ability, and Competence	System and Application Quick Reference Guides
Skills, Ability, and Competence, Attitudes, Patient-Provider Relationship	Physician Training and consultation
Skills, Ability, and Competence	Chatbot Support Application
Knowledge, Patient-Provider Relationship	Cybersickness questionnaire (CSQ-VR) as a pre-screening tool [2]
Environmental Context and Resources, System Quality	VR “Comfort” Package: face plate, extra battery pack, comfort head strap
Skills, Ability, and Competence, Effort Expectancy	In-App Tutorial
Environmental Context and Resources, Effort Expectancy, User Preferences	VR System / Menu Pre-Configuration and Optional Modalities (Sit v. Stand, Pass-thru)
Performance Expectancy	Outcome Evidence

Note: Implementation tools and techniques were mapped to barrier categories from the BEAR framework to ensure comprehensive coverage of known adoption barriers [1].

Table S3. Standardized four-task protocol completed by all participants during the experimental session, including task descriptions and requirements.

Task	Description
**Task 1:** Start-up and Initial Setup	Start the VR Headset, create a standing room boundary and adjust the physical environment, navigate the virtual environment, and launch the application "OpenBrush".
**Task 2:** In-App Setup	Start a new sketch.
**Task 3:** In-App Task Completion	Use the app "OpenBrush" to draw for up to 20 minutes. The drawing can be something abstract or concrete, and its significance is up to the participant. It will not be evaluated in any way as part of this study. The requirements for the drawing are as follows:<br>1. **Use 3 Colors:** Include at least three different colors from the app's color palette.<br>2. **Use 2 Brushes**: Experiment with at least two different brush types.<br>3. **Draw Initials**: Use selected tools to draw participant's initials on the virtual canvas<br>4. **Save the Drawing:** Use app's save function to store completed artwork.<br>5. **Clear Sketch:** After saving, clear the sketch<br>6. **Optional App Exploration -** Spend any remaining time exploring additional features of the app, such as trying more brushes and colors or using other features. Participants may choose not to continue exploring and discontinue use of the application and VR headset at this time.
**Task 4:** Shutdown the System	Exit the app, turn off the VR headset, remove the headset and place the system back in the box.

Note: All participants completed Tasks 1–4 in sequence. Task 3 had a 20-minute time limit for drawing; Tasks 1, 2, and 4 had time limits of 15, 5, and 5 minutes respectively.

Mock Physician Training Materials and VR Fitness Assessment

Overview of Clinician Training

Members of the research team participating in the study as the mock clinician received structured training designed to familiarize them with the VR therapeutic application, its intended use, and the experimental protocol. The training included a brief overview of virtual reality as a modality for delivering DTx, emphasizing its current evidence base, typical indications for use, and potential advantages and limitations in an at-home care setting. Team members were introduced to the specific VR headset and the application used in the study, including setup, navigation, and therapeutic features, to ensure they felt confident in providing instruction and support to participants.

Training also included a review of the standardized script used the clinician consultation. This script included language to describe the purpose of the VR therapy, expected benefits, how it complements standard care, and what participants should expect in terms of daily use. Emphasis was placed on consistency in language across all participants and on encouraging a supportive, non-technical framing to minimize intimidation or perceived burden. Team members were also instructed on how to address basic troubleshooting and where to direct participants for additional help. This training ensured fidelity across the physician-led intervention and helped reduce variability in how the technology was introduced.

Table S4. Structured prompts and clinician guidance used to standardize the mock physician consultation and VR fitness assessment delivered to participants in Condition 3 (Provider-Led Support) in a three-arm randomized pilot study of virtual reality therapeutics (VRx) conducted among healthy adults at the University of North Carolina at Chapel Hill (December 2024–February 2025).

**Mock Clinician Consultation Script**	
**Step 1: Introduction and General Greetings**	
**Objective:** Greet the participant and establish rapport; state the purpose of the consultation and set the stage for discussing anxiety management and introducing VRx as a potential therapy.	
**Rationale:** Establish acceptability by fostering trust and rapport, creating a positive environment to discuss the introduction of a new therapy.[40]	
**Action:**	**Physician:** Greet the participant warmly and establish rapport (How are you doing? How was …, etc.); introduce role as physician
	**Physician:** Show participant to seat and sit across from them, establish a collaborative tone for the session.
	**Physician:** State the purpose of the consultation - to discuss [participant's] anxiety symptoms and explore a new therapy option.
**Script:**	**Physician:** "Hello, [Participant's Name]. It's great to meet you. I understand you've been experiencing anxiety and are interested in treatment. My goal today is to understand how anxiety is affecting your day-to-day life and explore options that might help. I also want to share a new therapy option with you that could be a good fit. Does that sound okay?"
**Step 2: Discuss the Diagnosis**	
**Objective:**	Provide clarity about the participant's "mock anxiety diagnosis" in a way that is understandable, empathetic, and non-stigmatizing to normalize the experience of discussing symptoms and potential treatment options. Establish a connection between the diagnosis and need for targeted therapy like VRx.
**Rationale:**	Builds acceptance by clearly linking the participant's diagnosis with the benefits and outcomes VRx can provide, making the connection tangible and relevant.[45]
**Action:**	**Physician:** Introduce the treatment need, highlighting the importance of managing symptoms.
**Script:**	**Physician:** "Having anxiety can mean your body and mind are reacting as if you're in constant 'fight or flight' mode, even in situations where there's no immediate danger. It's important to know that anxiety is very common, and it's something we can absolutely work on together. You're not alone in this, and there are treatments that can really help manage these feelings and improve your quality of life. Does that make sense so far?"<br>***Pause for Participant Response***<br>**Physician:** "I'd like to recommend an innovative therapy called VRx, which uses virtual reality to help you learn relaxation techniques and build resilience in a safe, controlled environment. It's been shown to be highly

	effective for managing symptoms like yours. What do you think about trying something like this?" ***Pause for Participant Response*** **Physician:** "I'm going to evaluate you next to see if this therapy is a fit. The evaluation is simple, but we want to make sure that VRx won't cause any adverse effects. If you have any questions as we go along, let me know. I want to make sure you feel comfortable and confident moving forward with this plan."
**Step 3: Evaluating Fit for VRx**	
**Objective:**	Assess the participant's comfort with technology and willingness to try VRx; ensure they are not predisposed to cybersickness.
**Rationale:**	Ensure appropriateness, feasibility, behavioral alignment, and technology acceptance. Encourage technology acceptance through using participant centered approach to make treatment feel personalized.
**Action:**	Go through the cybersickness questionnaire to assess for cybersickness; demonstrate how the VR headset adjusts to fit different head sizes.
**Script:**	**Physician:** "Have you ever used Virtual Reality technology before, like a VR headset for gaming or other purposes?" ***Pause for Participant Response*** <br>**Physician:** Run through Cybersickness Questionnaire Handout <br>**Physician:** "Can you tell me about your typical day and where you might see yourself fitting in a 20-minute VR session?" ***Pause for Participant Response*** <br>**Physician:** "Do you have any concerns about using VR technology, like feeling dizzy or not having enough time for it?" ***Pause for Participant Response*** <br>**Physician:** "The VRx System is designed to be simple to use and we'll provide you with all the assistive and training materials you need to get started."
**Step 4: Prescribing VRx Therapy**	
**Objective:** Introduce VRx as an innovative therapy; explain how it works and its evidence-based effectiveness	
**Rationale:** Compassionate listening and education are an important foundation in the treatment of anxiety.[280] The physician should display optimal levels of warmth, concern, confidence genuineness, and professionalism.	
**Action:**	**Physician:** Pick up VR Headset and controllers
**Script:**	**Physician:** "Using VR therapy is simple. You'll put on the headset and the device will prompt you to set-up a boundary. This boundary helps to make sure that you don't run into anything while you're in the virtual environment. Make sure there's nothing in the way of your boundary. Then, once you start the application "Open Brush", the program will guide you through how to use the different functionality. I'd like you to use the Art Therapy program for 20 minutes today. Draw whatever you like. I'd like for you to use three colors and two brushes at least, and when you're done, add your initials to the drawing. I'd like for you to save the drawing, then clear the sketch. If there's still time left, you can keep exploring the app or you can

	stop the treatment for today. It's important to follow the instructions and let me know if you experience any symptoms such as nausea, dizziness, or disorientation. Do you have any questions or concerns I can help you with?"

References

1. Camacho J, Zanoletti-Mannello M, Landis-Lewis Z, Kane-Gill SL, Boyce RD. A Conceptual Framework to Study the Implementation of Clinical Decision Support Systems (BEAR): Literature Review and Concept Mapping. Journal of Medical Internet Research JMIR Publications Inc., Toronto, Canada; 2020 Aug 6;22(8):e18388. doi: 10.2196/18388

2. Kourtesis P, Linnell J, Amir R, Argelaguet F, MacPherson SE. Cybersickness in Virtual Reality Questionnaire (CSQ-VR): A Validation and Comparison against SSQ and VRSQ. Virtual Worlds Multidisciplinary Digital Publishing Institute; 2023 Mar;2(1):16–35. doi: 10.3390/virtualworlds2010002
