# Supplementary material for: Evaluating the Effects of Clinician Prescribing and Implementation Materials on Adoption of Virtual Reality Therapeutics: Randomized Feasibility Pilot Study
Source: JMIR XR Spat Comput. 2026 Jun 30;3:e90626. doi: 10.2196/90626 (PMC13317682; doi:10.2196/90626)
Supplement: Multimedia Appendix 4 [file xr-v3-e90626-s004.pdf]

Questionnaires

This appendix presents the pre- and post-intervention questionnaires administered in this study, including the Technology Acceptance Model (TAM) items [1–3], self-efficacy and satisfaction items, the modified System Usability Scale (SUS)[4], and the Cybersickness in Virtual Reality Questionnaire (CSQ-VR) [5].

Table S1. Acceptability Pre- and Post-Assessment Questions (Technology Acceptance Model)

**Measurement**	**Statement**	**Pre or Post Test**
Perceived Usefulness (PU)	I believe that virtual reality is useful for managing health-related symptoms.	Pre
	I believe that using virtual reality is useful for managing health-related symptoms.	Post
	Using virtual reality can improve my ability to manage health-related symptoms.	Pre
	Using virtual reality can improve my ability to manage health-related symptoms.	Post
Perceived Ease of Use (PEU)	I expect this VR system to be easy to use.	Pre
	I found this VR system easy to use.	Post
	Learning to use virtual reality will be straightforward.	Pre
	It was easy to learn how to operate the VR system.	Post
Attitude Towards Technology (ATT)	I feel positive about using virtual reality for health interventions.	Pre
	I feel positive about using virtual reality for health interventions.	Post
	I think using virtual reality technology for health-related purposes is a good idea.	Pre
	I think using virtual reality technology for health-related purposes is a good idea.	Post
Behavioral Intention to Use (BI)	Outside of participating in this study, I plan to use virtual reality technology for health purposes in the future.	Pre
	I am likely to use virtual reality technology for health purposes in the future.	Post
	I am willing to adopt virtual reality technology as part of my health management routine.	Pre
	I am willing to adopt virtual reality technology as part of my health management routine.	Post

Note: All items were rated on a 4-point Likert scale (1=Strongly Disagree, 2=Disagree, 3=Agree, 4=Strongly Agree). Pre-intervention items were administered prior to VRx exposure; post-intervention items were administered immediately following VRx exposure.

Table S2. Self-efficacy and satisfaction statements used to measure acceptance.

Measurement	Statement
Self-Efficacy (SE)	I felt confident using the VR system.
	I would need technical support to use this VR system. (reverse-scored)
	I needed to learn a lot of things before I could use the VR system. (reverse-scored)
Satisfaction	I am satisfied with my overall experience using the VR system.
	My expectations for using the VR system were met.
Note: All items were rated on a 4-point Likert scale (1=Strongly Disagree, 2=Disagree, 3=Agree, 4=Strongly Agree). Items marked as reverse-scored were recoded prior to analysis. All items were administered post-intervention only.	

Table S3. SUS Questions.

SUS Question	Post-Intervention Question	SUS Measurement
SUS01	I think I would use this system frequently.	Frequency of Use
SUS02	I found the system unnecessarily complex.	Complexity
SUS03	I thought the system was easy to use.	Ease of use
SUS04	I think that I would need the support of a technical person to be able to use this system.	Need for assistance
SUS07	I would imagine that most people would learn to use this system very quickly.	Learnability
SUS08	I found the system cumbersome to use.	Usability Hindrance
SUS09	I felt confident using the system.	Confidence in Use
SUS10	I needed to learn a lot of things before I could get going with this system.	Learnability
Note: All items were rated on a 4-point Likert scale (1=Strongly Disagree, 2=Disagree, 3=Agree, 4=Strongly Agree). SUS05 ("I found the various functions in this system were well integrated") and SUS06 ("I thought there was too much inconsistency in this system") were omitted due to limited system complexity. Scores were rescaled to maintain a 0–100 range consistent with standard SUS scoring conventions. All items were administered post-intervention only.		

Table S4. Cybersickness in Virtual Reality Screening test administered pre- and post-intervention.

Can you rate how noticeable and intense each of these symptoms is for you on a scale from 1 to 7, where 1 is 'not present' and 7 is 'very intense'?

Are you currently experiencing any nausea (e.g., stomach pain)?

1	2	3	4	5	6	7
Absent feeling	Very mild feeling	Mild feeling	Moderate feeling	Intense feeling	Very intense feeling	Extreme feeling

Are you currently experiencing any dizziness (e.g., light-headedness or spinning feeling)?

1	2	3	4	5	6	7
Absent feeling	Very mild feeling	Mild feeling	Moderate feeling	Intense feeling	Very intense feeling	Extreme feeling

Are you currently experiencing feelings of disorientation (e.g., spatial confusion or vertigo)?

1	2	3	4	5	6	7
Absent feeling	Very mild feeling	Mild feeling	Moderate feeling	Intense feeling	Very intense feeling	Extreme feeling

Are you currently experiencing any feelings of imbalance or instability?

1	2	3	4	5	6	7
Absent feeling	Very mild feeling	Mild feeling	Moderate feeling	Intense feeling	Very intense feeling	Extreme feeling

Are you currently experiencing any symptoms of a headache or head pain?

1	2	3	4	5	6	7
Absent feeling	Very mild feeling	Mild feeling	Moderate feeling	Intense feeling	Very intense feeling	Extreme feeling

Are you currently experiencing any visual discomfort such as blurred vision?

1	2	3	4	5	6	7
Absent feeling	Very mild feeling	Mild feeling	Moderate feeling	Intense feeling	Very intense feeling	Extreme feeling

If the total score is >30, the participant is not a fit for VRx, as this reflects significant susceptibility to cybersickness.

If any single symptom is rated at 6 (very intense) or 7 (extreme feeling), the participant is not a fit for VR, as this reflects significant discomfort that could worsen with use.

References

1. Davis FD. Perceived Usefulness, Perceived Ease of Use, and User Acceptance of Information Technology. MIS Quarterly 1989 Sept;13(3):319. doi: 10.2307/249008

2. Venkatesh V, Bala H. Technology Acceptance Model 3 and a Research Agenda on Interventions. Decision Sciences 2008;39(2):273–315. doi: 10.1111/j.1540-5915.2008.00192.x

3. Venkatesh V, Davis FD. A Theoretical Extension of the Technology Acceptance Model: Four Longitudinal Field Studies. Management Science INFORMS; 2000 Feb;46(2):186–204. doi: 10.1287/mnsc.46.2.186.11926

4. Affairs AS for P. System Usability Scale (SUS). Department of Health and Human Services; 2013. Available from: https://www.usability.gov/how-to-and-tools/methods/system-usability-scale.html [accessed Sept 28, 2023]

5. Kourtesis P, Linnell J, Amir R, Argelaguet F, MacPherson SE. Cybersickness in Virtual Reality Questionnaire (CSQ-VR): A Validation and Comparison against SSQ and VRSQ. Virtual Worlds Multidisciplinary Digital Publishing Institute; 2023 Mar;2(1):16–35. doi: 10.3390/virtualworlds2010002
