# Supplementary material for: Evaluating the Effects of Clinician Prescribing and Implementation Materials on Adoption of Virtual Reality Therapeutics: Randomized Feasibility Pilot Study
Source: JMIR XR Spat Comput. 2026 Jun 30;3:e90626. doi: 10.2196/90626 (PMC13317682; doi:10.2196/90626)
Supplement: Multimedia Appendix 5 [file xr-v3-e90626-s005.pdf]

University of North Carolina at Chapel Hill
Participant Observation Guide
IRB Study #: 24-2894
Study Title: Exploring the Effects of Prescribing and Implementation Techniques on the Adoption of Virtual Reality Therapeutics
Principal Investigator: Ashlyn Zebrowski, MS

Component	Participant (Pt) Information	Comments
Study ID		
Age		
Gender		
Assigned Condition (1, 2, 3)		
Technology Comfort Score		

Observational Checklist

Complete yellow sections for Conditions 2 and 3 **only**.

Complete green sections for Condition 3 **only.**

**Task 1: Start the VR Headset, create a standing room boundary and adjust the physical environment, navigate the virtual environment, and launch the application "OpenBrush". Remember to think aloud as you complete this task.**<br><br>**Time Limit: 15 minutes**	
Time Start:	
Time End:	
Completed before time limit? (Y/N)	

**Task Observations (Mark all that were completed)**		
**Condition 2, 3: Using the provided assistive and training materials**	☐ Participant reviewed the evidence about the treatment<br>☐ Participant reviewed the Quick Reference Guide	**Error? Y/N**
**Starting the VR Headset**	☐ Participant correctly identifies the power button.<br>☐ Participant successfully powers on the headset.<br>☐ Participant verbalizes any confusion or steps while turning the headset on.	**Error? Y/N**
**Setting up the Boundary**	☐ Participant appeared to understand the prompt to set up the virtual boundary.<br>☐ Participant correctly uses the controller to define boundary.<br>☐ Participant verbalizes their thought process about setting up the space.<br>☐ Participant adjusts physical environment (e.g., moves objects)	**Error? Y/N**
**Navigating the Virtual Environment**	☐ Participant successfully navigates to the main menu in the virtual environment.<br>☐ Participant identifies the appropriate controls<br>☐ Participant verbalizes challenges or successes.<br>☐ Participant adjusts their body posture or positioning to improve navigation.	**Error? Y/N**
**Launching "OpenBrush"**	☐ Participant correctly identifies and selects "OpenBrush".<br>☐ Participant verbalizes reasoning for incorrect menu selections or corrections if needed.<br>☐ "OpenBrush" launches.	**Error? Y/N**
**Think-Aloud Protocol**	☐ Participant verbalizes their thought process while completing each step.<br>☐ Participant articulates any confusion, questions, or issues.	**Error? Y/N**

	☐ Participant maintains focus without unnecessary distractions		
**Additional Notes from Task Observations:**			

**Usability Assessment:**			**Score:**
1. Participant was able to complete the task without any errors.	**No**	**Yes**	**Comments**
a. (0) Participant did not complete the task within the allotted period	0		
b. (5) Participant completed the task with simple errors (e.g., *Did not press power button long enough, had to readjust the headset strap*)		5	
c. (5) Participant attempted to use the troubleshooting flowchart to resolve error but required facilitator assistance.		5	
d. (8) Participant used the troubleshooting flowchart to resolve an error and was able to complete the task.		8	
e. (10) Participant completed the task without any errors		10	
Errors Observed:			
2. Participant was able to complete the task without requesting assistance from the facilitator.	**No**	**Yes**	**Comments**
a. (0) Participant requested assistance more than once	0		
b. (5) Participant requested assistance only one time		5	
c. (8) Participant engaged with the Support Chat Function or with the "physician" and did not request assistance from the study facilitator.		8	
d. (10) Participant completed task without requesting assistance		10	
Describe the Assistance Requested:			
**Acceptability and Behavioral Assessment:**			**Score:**
3. Participant did not experience any frustration or discomfort during the task.	**No**	**Yes**	**Comments**
a. (0) Participant expressed frustration frequently (*e.g., sighing, exclaiming, pausing*); frustration impacted task progress.	0		
b. (5) Participant expressed frustration (*e.g., signing, exclaiming, pausing*) but was able to complete the task.		5	
c. (10) Participant expressed no frustration and completed the task confidently.		10	
4. Participant demonstrated confidence while completing the task.	**No**	**Yes**	**Comments**
a. (0) Participant appeared hesitant and unsure throughout the task (*e.g., pausing, verbalizing doubts*)	0		
b. (5) Participant showed intermittent confidence or increased confidence as they became more comfortable with the system.		5	
c. (10) Participant demonstrated consistent confidence throughout the task (*e.g., completing steps quickly without hesitation*)		10	
5. Participant displayed comfort while interacting with the VR headset and VR environment.	**No**	**Yes**	**Comments**
a. (0) Participant appeared visibly uncomfortable (*e.g., hesitant movements, verbal discomfort*)	0		
b. (5) Participant appeared neutral or slightly uncomfortable but completed the task.		5	

	No	Yes	Comments
c. (10) Participant appeared comfortable and at ease.		10	
6. Participant was positive and excited about using VRx.	**No**	**Yes**	**Comments**
a. (0) Participant displayed anxiety or stress that might serve as a barrier when engaging with VR.	0		
b. (5) Participant initially was apprehensive but displayed positive emotions (*e.g., a smile, nod, or verbal acknowledgment*) once they settled into the task		5	
c. (10) Participant was visibly excited or interested.		10	
Describe any other acceptability or behavioral observations:			
**Fidelity Assessment:**			**Score:**
7. Participant followed the correct sequence of steps as outlined in the provided protocol.	**No**	**Yes**	**Comments**
a. (0) Participant was unable to complete steps, skipped multiple steps, made multiple errors, or completed steps incorrectly (*e.g., created a seated boundary rather than a standing boundary*)	0		
b. (5) Participant skipped or completed one step incorrectly but self-corrected without requesting assistance.		5	
c. (10) Participant followed all steps in the correct sequence without errors.		10	
8. Participant completed tasks accurately and consistently.			
a. (0) Participant demonstrated significant inconsistencies, requiring repeated corrections or assistance.	0		
b. (5) Participant demonstrated minor inconsistencies but completed tasks without needing assistance.		5	
c. (10) Participant executed all tasks consistently and accurately according to the protocol.		10	
Describe any other observations related to fidelity of use:			
**Technology Acceptance:**	**No**	**Yes**	**Comments**
Initial reactions: Are there visible signs of excitement or interest when first introduced to the VR headset?			
Initial reactions: Are there visible signs of apprehension or reluctance when first introduced to the VR headset?			
Is participant visibly more engaged when the task becomes enjoyable or rewarding?			
Are there visible signs of frustration or disengagement due to the complexity of the system and set-up?			
Are there visible signs of increased engagement or excitement due to the assistive or training materials?			
Are there visible signs of frustration or disengagement due to the assistive or training materials?			
Describe any other observations related to technology acceptance:			

Additional Questions for Conditions 2 and 3 Acceptability and Behavioral Assessment			Score:
9. Participant effectively used the provided materials (*e.g., quick reference guide, troubleshooting flowchart, support chat*) to complete the task.	**No**	**Yes**	**Comments**
a. (0) Participant did not use any materials.	0		
b. (5) Participant used one or more materials but still struggled at times during the task.		5	
c. (10) Participant used the materials effectively and found. Them helpful in completing the task without frustration		10	
10. Participant balanced using multiple training materials to complete the task.	**No**	**Yes**	**Comments**
a. (0) Participant appeared overwhelmed or confused by the variety of materials and struggled to use them effectively.	0		
b. (5) Participant used some materials but needed assistance or struggled with integrating them.		5	
c. (8) Condition 3 only - Participant did not use the materials but asked the "physician" questions during the "mock physician consultation"		8	
d. (10) Participant efficiently and independently used the provided materials to complete the task.		10	
11. Participant used provided materials (e.g., flowchart, support chat) to effectively troubleshot and resolve errors.	**No**	**Yes**	**Comments**
a. (0) Participant was unable to resolve errors even with the provided materials.	0		
b. (5) Participant resolved errors with some difficulty but ultimately succeeded.		5	
c. (10) Participant resolved errors efficiently and confidently using the provided materials		10	
**12. Check off which materials participant used:**	a. Step-by-Step Clickable Tutorial<br>b. Tutorial Videos<br>c. Quick Reference Guide for VR Set-up<br>d. Troubleshooting Flow Chart<br>e. Support Chat<br>*f. (Condition 3) – Engaged with Prescriber*		
13. **For Condition 3 Only (role of the Physician Consultation),** Participant felt confident about using the VR system after receiving instructions during the mock physician consultation.	**No**	**Yes**	**Comments**
a. (0) Participant expressed confusion or lack of confidence even after the consultation.	0		
b. (5) Participant initially expressed doubts but became more confident after referring to the physician.		5	
c. (10) Participant felt fully confident and prepared after the consultation.		10	
14. Participant displayed increased comfort with VR system once receiving the physician's instructions.	**No**	**Yes**	**Comments**
a. (0) Participant showed continued discomfort despite the additional instructions and materials.	0		
b. (5) Participant became more comfortable with some residual hesitation.		5	
c. (10) Participant demonstrated complete comfort with the system.		10	

Additional Observations from Condition 2 and 3 related to Acceptability and Behavioral Assessment:			
**Additional Questions for Conditions 2 and 3 Fidelity**			**Score:**
15. Participant utilized the provided assistive and training materials as intended.	**No**	**Yes**	**Comments**
a. (0) Participant frequently skipped portions of the training materials or did not use the materials at all.	0		
b. (5) Participant used some of the materials but did not use them effectively.		5	
c. (10) Participant used the materials effectively and found them helpful in completing the task.		10	
16. Participant completed the tasks within the expected timeframe.	**No**	**Yes**	**Comments**
a. (0) Participant took significantly longer than expected or failed to complete tasks.	0		
b. (5) Participant completed tasks with some delays due to errors, hesitation, or confusion related to the materials.		5	
c. (10) Participant completed tasks efficiently within the expected time.		10	

**Task 2: In the "OpenBrush" app, start a new sketch.**<br><br>**Time Limit: 5 minutes**	
Time Start:	
Time End:	
Completed before time limit? (Y/N)	

**Task Observations (Mark all that were completed)**		
**Watch the Tutorial Video**	☐ Participant starts the in-app tutorial video / step-by-step guide<br>☐ Participant completes the in-app tutorial video / step-by-step guide	**Error? Y/N**
**Navigate to start New Sketch**	☐ Participant successfully navigates to the main menu in the app.<br>☐ Participant locates the start a new sketch in the app menu	**Error? Y/N**
**Select Sketch Properties**	☐ Participant explores if any additional options are visible when starting a new sketch (e.g., layers, background settings)	**Error? Y/N**
**Think-Aloud Protocol**	☐ Participant verbalizes their thought process while completing each step.<br>☐ Participant articulates any confusion, questions, or issues encountered.<br>☐ Participant maintains focus without unnecessary distractions	**Error? Y/N**
**Additional Notes from Task Observations:**		

**Usability Assessment:**			**Score:**
1. Participant was able to complete the task without any errors.	**No**	**Yes**	**Comments**
a. (0) Participant did not complete the task within the allotted period	0		
b. (5) Participant completed the task with simple errors (e.g., *clicked into the wrong menu button*)		5	
c. (10) Participant completed the task without any errors		10	
Errors Observed:			
2. Participant was able to complete the task without requesting assistance from the facilitator.	**No**	**Yes**	**Comments**
a. (0) Participant requested assistance more than once	0		
b. (5) Participant requested assistance only one time		5	
c. (5) Participant attempted to use the troubleshooting flowchart to resolve error but required facilitator assistance.		5	
d. (8) Participant used the troubleshooting flowchart to resolve an error and was able to complete the task.		8	
e. (10) Participant completed task without requesting assistance		10	
Describe the Assistance Requested:			

**Acceptability and Behavioral Assessment:**			**Score:**
3. Participant did not experience any frustration or discomfort during the task.	**No**	**Yes**	**Comments**
a. (0) Participant expressed frustration frequently (*e.g., sighing, exclaiming, pausing*); frustration impacted task progress.	0		
b. (5) Participant expressed frustration (*e.g., sighing, exclaiming, pausing*) but was able to complete the task.		5	
c. (10) Participant expressed no frustration and completed the task confidently.		10	

		No	Yes	Comments
4.	Participant demonstrated confidence while completing the task.	**No**	**Yes**	**Comments**
a.	(0) Participant appeared hesitant and unsure throughout the task (*e.g., pausing, verbalizing doubts*)	0		
b.	(5) Participant showed intermittent confidence or increased confidence as they became more comfortable with the system.		5	
c.	(10) Participant demonstrated consistent confidence throughout the task (*e.g., completing steps quickly without hesitation*)		10	
5.	Participant displayed comfort while interacting with the VR headset and VR environment.	**No**	**Yes**	**Comments**
a.	(0) Participant appeared visibly uncomfortable (*e.g., hesitant movements, verbal discomfort*)	0		
b.	(5) Participant appeared neutral or slightly uncomfortable but completed the task.		5	
c.	(10) Participant appeared comfortable and at ease.		10	
6.	Participant was positive and excited about using VRx.	**No**	**Yes**	**Comments**
a.	(0) Participant displayed anxiety or stress that might serve as a barrier when engaging with VR.	0		
b.	(5) Participant initially was apprehensive but displayed positive emotions (*e.g., a smile, nod, or verbal acknowledgment*) once they settled into the task		5	
c.	(10) Participant was visibly excited or interested.		10	
	Describe any other acceptability or behavioral observations:			
	**Fidelity Assessment:**			**Score:**
7.	Participant followed the correct sequence of steps as outlined in the provided protocol.	**No**	**Yes**	**Comments**
a.	(0) Participant was unable to complete steps, skipped multiple steps, made multiple errors, or completed steps incorrectly (*e.g., opened a saved sketch, opened a sketch from the library*)	0		
b.	(5) Participant skipped or completed one step incorrectly but self-corrected without requesting assistance.		5	
c.	(10) Participant followed all steps in the correct sequence without errors.		10	
8.	Participant completed tasks accurately and consistently.			
a.	(0) Participant demonstrated significant inconsistencies, requiring repeated corrections or assistance.	0		
b.	(5) Participant demonstrated minor inconsistencies but completed tasks without needing assistance.		5	
c.	(10) Participant executed all tasks consistently and accurately according to the protocol.		10	
	Describe any other observations related to fidelity of use:			
	**Technology Acceptance:**	**No**	**Yes**	**Comments**
	Are there visible signs of excitement or interest when conducting the tasks in the VR headset?			
	Are there visible signs of apprehension or reluctance when conducting the tasks in the VR headset?			

Is participant visibly more engaged when the task becomes enjoyable or rewarding?			
Are there visible signs of frustration or disengagement due to the complexity of the application?			
Are there visible signs of frustration or disengagement due to the in-app tutorial video?			
Does the participant fully watch the in-app tutorial video?			
Describe any other observations related to technology acceptance:			

Task 3: Use the app "OpenBrush" to draw for up to 20 minutes. The drawing can be something abstract or concrete, and its significance is up to the participant. It will not be evaluated in any way as part of this study. The requirements for the drawing are as follows:

1. **Use 3 Colors:** Include at least three different colors from the app's color palette.
2. **Use 2 Brushes:** Experiment with at least two different brush types.
3. **Draw Initials:** Use selected tools to draw your initials on the virtual canvas.
4. **Save the Drawing:** Use app's save function to store completed artwork.
5. **Clear Sketch:** After saving, clear the sketch.
6. **Optional App Exploration -** Spend any remaining time exploring additional features of the app, such as trying more brushes and colors or using other features. Participants may choose not to continue exploring and discontinue use of the application and VR headset at this time.

Time Limit: 20 minutes

Time Start:	
Time End:	
Completed before time limit? (Y/N)	

Task Observations (Mark all that were completed)

**Use 3 Colors**	☐ Participant successfully selected the first color from the palette.<br>☐ Participant successfully selected the second color from the palette.<br>☐ Participant successfully selected the third color from the palette.<br>☐ Participant successfully incorporated all three colors into drawing	**Error? Y/N**
**Use 2 Brushes**	☐ Participant successfully selected the first brush type.<br>☐ Participant successfully selected the second brush type.<br>☐ Participant incorporated both brush types into their drawing.	**Error? Y/N**
**Draw Initials**	☐ Participant uses the tools to draw their initials.<br>☐ The initials are visible and distinguishable	**Error? Y/N**
**Save Drawing**	☐ Participant accessed the save function within the app.<br>☐ Participant successfully saved their completed drawing.	**Error? Y/N**
**Clear Sketch**	☐ Participant located the clear sketch/reset function in the app.<br>☐ Participant cleared the canvas after saving the drawing.	**Error? Y/N**
**Think-Aloud Protocol**	☐ Participant verbalizes thought process while completing each step.<br>☐ Participant articulates any confusion, questions, or issues.<br>☐ Participant maintains focus without unnecessary distractions	**Error? Y/N**
**Optional Exploration, Time, and Engagement Tracking**	☐ Participant spent up to 20 minutes working on their drawing<br>☐ If less than 20 minutes was spent on drawing, Participant chose to continue to explore the app after completing their drawing.<br>☐ Participant remained engaged throughout the task (e.g., no prolonged idle periods, actively explored features)	**Notes:**
**Additional Notes from Task Observations:**		

**Usability Assessment:**			**Score:**
1. Participant was able to complete the task without any errors.	**No**	**Yes**	**Comments**
a. (0) Participant did not complete the task within the allotted period	0		
b. (5) Participant completed the task with simple errors (e.g., *did not use three colors or two brushes, initials not legible*)		5	
c. (10) Participant completed the task without any errors		10	

Errors Observed:

2. Participant was able to complete the task without requesting assistance from the facilitator.	**No**	**Yes**	**Comments**
a. (0) Participant requested assistance more than once	0		
b. (5) Participant requested assistance only one time		5	
c. (10) Participant completed task without requesting assistance		10	
Describe the Assistance Requested:			
**Acceptability and Behavioral Assessment:**			**Score:**
3. Participant did not experience any frustration or discomfort during the task.	**No**	**Yes**	**Comments**
a. (0) Participant expressed frustration frequently (*e.g., sighing, exclaiming, pausing*); frustration impacted task progress.	0		
b. (5) Participant expressed frustration (*e.g., sighing, exclaiming, pausing*) but was able to complete the task.		5	
c. (10) Participant expressed no frustration and completed the task confidently.		10	
4. Participant demonstrated confidence while completing the task.	**No**	**Yes**	**Comments**
a. (0) Participant appeared hesitant and unsure throughout the task (*e.g., pausing, verbalizing doubts*)	0		
b. (5) Participant showed intermittent confidence or increased confidence as they became more comfortable with the system.		5	
c. (10) Participant demonstrated consistent confidence throughout the task (*e.g., completing steps quickly without hesitation*)		10	
5. Participant displayed comfort while interacting with the VR headset and VR environment.	**No**	**Yes**	**Comments**
a. (0) Participant appeared visibly uncomfortable (*e.g., hesitant movements, verbal discomfort*)	0		
b. (5) Participant appeared neutral or slightly uncomfortable but completed the task.		5	
c. (10) Participant appeared comfortable and at ease.		10	
6. Participant was positive and excited about using VRx.	**No**	**Yes**	**Comments**
a. (0) Participant displayed anxiety or stress that might serve as a barrier when engaging with VR.	0		
b. (5) Participant initially was apprehensive but displayed positive emotions (*e.g., a smile, nod, or verbal acknowledgment*) once settled into the task		5	
c. (10) Participant was visibly excited or interested.		10	
7. Participant explored additional app features beyond the task requirements.	**No**	**Yes**	
a. (0) Participant disengaged with the system as soon as their tasks were completed.	0		
b. (5) Participant explored for a short time but ultimately decided to disengage with the system early.		5	

c. (10) Participant explored until the time limit.		10	
Describe any other acceptability or behavioral observations:			
**Fidelity Assessment:**			**Score:**
8. Participant followed the correct sequence of steps as outlined in the provided protocol.	**No**	**Yes**	**Comments**
a. (0) Participant was unable to complete steps, skipped multiple steps, made multiple errors, or completed steps incorrectly (*e.g., could not change the colors or change brushes, unable to save drawing*)	0		
b. (5) Participant skipped or completed one step incorrectly but self-corrected without requesting assistance.		5	
c. (10) Participant followed all steps in the correct sequence without errors.		10	
9. Participant completed tasks accurately and consistently.			
a. (0) Participant demonstrated significant inconsistencies, requiring repeated corrections or assistance.	0		
b. (5) Participant demonstrated minor inconsistencies but completed tasks without needing assistance.		5	
c. (10) Participant executed all tasks consistently and accurately according to the protocol.		10	
Describe any other observations related to fidelity of use:			
**Technology Acceptance:**	**No**	**Yes**	**Comments**
Are there visible signs of excitement or interest when using the "OpenBrush" app to draw?			
Are there visible signs of apprehension or reluctance when using the "OpenBrush" app?			
Is participant visibly more engaged when the task becomes enjoyable or rewarding?			
Are there visible signs of frustration or disengagement due to the complexity of the application?			
Did the participant verbalize thoughts or express emotions (e.g., *satisfaction, frustration*) that give indication to their acceptance of the technology?			
Did the participant experiment creatively with the app (e.g., combining colors, testing advanced brush effects)?			
Describe any other observations related to technology acceptance:			

**Task 4: Exit the app, turn off the VR headset, remove the headset and place the system back in the storage container.**<br><br>**Time Limit: 5 minutes**	
Time Start:	
Time End:	
Completed before time limit? (Y/N)	

**Task Observations (Mark all that were completed)**		
**Exit App**	☐ Participant accessed the app menu to locate the exit/close function<br>☐ Participant successfully exited the "OpenBrush" app.	**Error? Y/N**
**Turn Off VR Headset**	☐ Participant located the power button on the VR headset.<br>☐ Participant used the button correctly to power off the device.<br>☐ Participant confirmed device was powered off (e.g*., no lights, screen is blank*)	**Error? Y/N**
**Remove Headset**	☐ Participant removes headset.	**Error? Y/N**
**Return System to Box**	☐ Participant collected all components of the VR system (e.g., *headset, controllers*)<br>☐ Participant returns the device to the storage container.	**Error? Y/N**
**Think-Aloud Protocol**	☐ Participant verbalizes their thought process while completing each step.<br>☐ Participant articulates any confusion, questions, or issues encountered.<br>☐ Participant maintains focus without unnecessary distractions	**Error? Y/N**
**Additional Notes from Task Observations:**		

**Usability Assessment:**			**Score:**
1. Participant was able to complete the task without any errors.	**No**	**Yes**	**Comments**
a. (0) Participant did not complete the task within the allotted period	0		
b. (5) Participant completed the task with simple errors (e.g., *did not exit app before powering off headset*)		5	
c. (10) Participant completed the task without any errors		10	
Errors Observed:			
2. Participant was able to complete the task without requesting assistance from the facilitator.	**No**	**Yes**	**Comments**
a. (0) Participant requested assistance more than once	0		
b. (5) Participant requested assistance only one time		5	
c. (10) Participant completed task without requesting assistance		10	
Describe the Assistance Requested:			

**Acceptability and Behavioral Assessment:**			**Score:**
3. Participant did not experience any frustration or discomfort during the task.	**No**	**Yes**	**Comments**
a. (0) Participant expressed frustration frequently (*e.g., sighing, exclaiming, pausing*); frustration impacted task progress.	0		
b. (5) Participant expressed frustration (*e.g., sighing, exclaiming, pausing*) but was able to complete the task.		5	

c. (10) Participant expressed no frustration and completed the task confidently.		10	
4. Participant demonstrated confidence while completing the task.	**No**	**Yes**	**Comments**
a. (0) Participant appeared hesitant and unsure throughout the task (*e.g., pausing, verbalizing doubts*)	0		
b. (5) Participant showed intermittent confidence or increased confidence as they became more comfortable with the system.		5	
c. (10) Participant demonstrated consistent confidence throughout the task (*e.g., completing steps quickly without hesitation*)		10	
5. Participant displayed comfort while interacting with the VR headset and VR environment.	**No**	**Yes**	**Comments**
a. (0) Participant appeared visibly uncomfortable (*e.g., hesitant movements, verbal discomfort*)	0		
b. (5) Participant appeared neutral or slightly uncomfortable but completed the task.		5	
c. (10) Participant appeared comfortable and at ease.		10	
6. Participant was positive and excited about using VRx.	**No**	**Yes**	**Comments**
a. (0) Participant displayed anxiety or stress that might serve as a barrier when engaging with VR.	0		
b. (5) Participant initially was apprehensive but displayed positive emotions (*e.g., a smile, nod, or verbal acknowledgment*) once complete.		5	
c. (10) Participant was visibly excited or interested.		10	
Describe any other acceptability or behavioral observations:			
**Fidelity Assessment:**			**Score:**
7. Participant followed the correct sequence of steps as outlined in the provided protocol.	**No**	**Yes**	**Comments**
a. (0) Participant was unable to complete steps, skipped multiple steps, made multiple errors, or completed steps incorrectly (*e.g., could not change the colors or change brushes, unable to save drawing*)	0		
b. (5) Participant skipped or completed one step incorrectly but self-corrected without requesting assistance.		5	
c. (10) Participant followed all steps in the correct sequence without errors.		10	
8. Participant completed tasks accurately and consistently.	**No**	**Yes**	**Comments**
a. (0) Participant demonstrated significant inconsistencies, requiring repeated corrections or assistance.	0		
b. (5) Participant demonstrated minor inconsistencies but completed tasks without needing assistance.		5	
c. (10) Participant executed all tasks consistently and accurately according to the protocol.		10	

Describe any other observations related to fidelity of use:			
**Technology Acceptance:**	**No**	**Yes**	**Comments**
Are there visible signs of excitement or interest after using the VR system?			
Are there visible signs of apprehension or disappointment after using the VR system?			
Are there visible signs of frustration or disengagement due to the complexity of the application?			
Did the participant verbalize thoughts or express emotions (e.g., *satisfaction, frustration*) that give indication to their acceptance of the technology?			
Did the participant experiment creatively with the app (e.g., combining colors, testing advanced brush effects)?			
Describe any other observations related to technology acceptance:			
