# Supplementary material for: Evaluating the Effects of Clinician Prescribing and Implementation Materials on Adoption of Virtual Reality Therapeutics: Randomized Feasibility Pilot Study
Source: JMIR XR Spat Comput. 2026 Jun 30;3:e90626. doi: 10.2196/90626 (PMC13317682; doi:10.2196/90626)
Supplement: Multimedia Appendix 6 [file xr-v3-e90626-s006.pdf]

Statistical Methods and Exploratory Analyses

1. Overview of Analytic Approach

This appendix provides comprehensive documentation of the statistical methods, normality assessments, effect size calculations, and exploratory analyses conducted in this study. Analyses were structured into three tiers: primary, secondary, and exploratory, consistent with the hypothesis-generating objectives of a feasibility pilot study. The rationale for the analytic approach and the handling of multiple comparisons are described below, followed by detailed results of exploratory analyses referenced in the main manuscript.

2. Normality Assessment

Data normality was assessed using Shapiro-Wilk tests prior to selection of statistical methods. Variables demonstrating significant departures from normality ($P<.05$) were analyzed using nonparametric methods. Results are summarized in Table S1.

Table S1. Shapiro-Wilk normality test results for primary study variables (N = 31).

Variable	W Statistic	P Value	Distribution Assessment
**TAM Overall (change score)**	0.919	.022	Non-normal
**PU (change score)**	0.718	<.001	Non-normal
**PEU (change score)**	0.871	.001	Non-normal
**ATT (change score)**	0.738	<.001	Non-normal
**BI (change score)**	0.899	.007	Non-normal
**SUS Total**	0.973	.615	Normal
**Task Completion Rate**	0.916	.019	Non-normal
**VR Engagement**	0.672	<.001	Non-normal
**Acceptability Percent**	0.825	<.001	Non-normal
**Fidelity Percent**	0.902	.008	Non-normal
**Usability Percent**	0.949	.150	Normal
**Time in VRx**	0.829	<.001	Non-normal

Note: For TAM constructs, normality was assessed on pre-post difference scores to evaluate the assumption for paired comparisons. SUS was administered post-intervention only and assessed as a single distribution. P<.05 indicates significant departure from normality. Given that the majority of variables violated normality assumptions, nonparametric tests (Wilcoxon signed-rank, Kruskal-Wallis) were used as the primary analytic approach.

3. Complete Specification of Statistical Tests

Table S2 provides a complete specification of all statistical tests used in the study, organized by analysis tier. This table is intended to facilitate transparency and replicability.

Table S2. Complete specification of statistical tests, effect size measures, confidence interval methods, and multiple comparison corrections by analysis tier.

Analysis Level	Comparison	Statistical Test	Effect Size	95% CI Method	Multiple Comparison Correction
Primary	Overall TAM score pre-post change (full sample)	Wilcoxon signed-rank (paired, two-tailed)	$r = Z/\sqrt{N}$	BCa bootstrap (2,000 resamples)	None
Secondary	TAM construct-level scores pre-post change (full sample)	Wilcoxon signed-rank (paired, two-tailed)	$r = Z/\sqrt{N}$	BCa bootstrap (2,000 resamples)	None
Secondary	Overall TAM score pre-post change by condition	Wilcoxon signed-rank (paired, two-tailed)	$r = Z/\sqrt{N}$	BCa bootstrap (2,000 resamples)	None
Secondary	Between-group differences: Time in VRx, Task Completion Rate, Engagement, Acceptability	Kruskal-Wallis H test	ηH^2	Not applicable	Bonferroni (post-hoc pairwise where significant)
Secondary	Post-hoc pairwise - Time in VRx	Wilcoxon rank-sum (unpaired)	Not reported	N/A	Bonferroni
Secondary	Between-group differences: Usability (SUS Total)	Kruskal-Wallis H test	ηH^2	N/A	None (omnibus test not significant)
Secondary	Between-group differences: Fidelity (Time-based Adherence, Task-based Adherence, Fidelity Score)	Kruskal-Wallis H test	ηH^2	Not applicable	Bonferroni (post-hoc pairwise where significant)
Secondary	Self-efficacy and satisfaction	Descriptive only	N/A	N/A	N/A
Exploratory	TAM construct-level pre-post change by condition	Wilcoxon signed-rank (paired, two-tailed)	$r = Z/\sqrt{N}$	BCa bootstrap (2,000 resamples)	None
Exploratory	SUS item-level by condition	Descriptive only	N/A	N/A	N/A
Exploratory	Subgroup analyses (demographics)	Kruskal-Wallis H test	Not reported	N/A	None

Note: Self-efficacy and satisfaction were summarized descriptively and not formally compared across conditions. Observed behavioral measures were also interpreted descriptively only.

4. Effect Size Formulas

Wilcoxon signed-rank effect size: $r = Z / \sqrt{N}$, where Z is the standardized test statistic from the Wilcoxon signed-rank test and N is the number of paired observations. Values of r are interpreted as: small (0.10–0.29), medium (0.30–0.49), and large (≥0.50), following Cohen (1988).

Kruskal-Wallis effect size: $\eta_H^2 = (H - k + 1) / (N - k)$, where H is the Kruskal-Wallis test statistic, k is the number of groups, and N is the total sample size.

95% confidence intervals for Wilcoxon r effect sizes were estimated using bias-corrected and accelerated (BCa) bootstrapping with 2,000 resamples, implemented in R.

5. Rationale for Family-Wise Error Approach

The analytic strategy for handling multiple comparisons in this study was informed by the exploratory objectives of the pilot design and established guidance for feasibility research [1,2].

Primary analyses (overall TAM pre-post change) and secondary analyses (condition-level TAM pre-post changes and between-group engagement comparisons) were treated as the primary inferential comparisons. Bonferroni corrections were applied to post-hoc pairwise comparisons following significant Kruskal-Wallis tests for between-group engagement, usability, and fidelity outcomes (Tables 8, 10, and 11 in manuscript).

Exploratory construct-level TAM analyses and item-level SUS analyses were not corrected for multiplicity, as the primary objective of these comparisons was hypothesis generation rather than confirmatory testing. This approach is consistent with recommendations from existing research that pilot studies are not designed for definitive hypothesis testing and that overly conservative corrections in small-sample exploratory research may obscure potentially meaningful signals warranting investigation in future adequately powered studies [1,2].

The associated risk of inflated Type I error is acknowledged in the Limitations section of the main manuscript.

6. TAM Construct-Level by Condition Inferential Results

Table S3 presents exploratory pre-post inferential results for each TAM construct (PU, PEU, ATT, BI) and overall TAM score within each experimental condition. Analyses were conducted using Wilcoxon signed-rank tests (paired, two-tailed), with effect sizes calculated as $r = Z/\sqrt{N}$ and 95% confidence intervals estimated using BCa bootstrapping (2,000 resamples). No corrections for multiple comparisons were applied; results are intended to characterize directional patterns across conditions rather than to support confirmatory inference.

Condition 3 (Provider-Led Support) demonstrated statistically significant improvements across all constructs and overall TAM. Condition 1 (No Support) showed significant improvement in BI and overall TAM only, with no change observed in PEU. Condition 2 (Self-Directed Support) reached significance for overall TAM only, with no individual construct achieving statistical significance. These patterns are consistent with the descriptive findings presented in Table 6 of the main manuscript and suggest that more structured implementation support may be associated with broader construct-level gains in technology acceptance.

Table S3. Exploratory pre-post changes in Technology Acceptance Model (TAM) construct scores by experimental condition.

**Condition**	**TAM Construct**	**n**	**Pre Mean (SD)**	**Post Mean (SD)**	**Δ Mean**	**W**	**P (Wilcoxon)**	**Effect Size (r [95% CI])**
**Condition 1** No Support	*PU*	10	2.80 (0.42)	3.20 (0.63)	+0.40	0	.072	0.57 [0.00, 0.81]
	PEU	10	3.00 (0.33)	3.00 (0.47)	+0.00	7.5	.100	0.00 [0.00, 0.66]
	ATT	10	3.10 (0.61)	3.35 (0.53)	+0.25	0	.089	0.54 [0.00, 0.77]
	BI	10	2.45 (0.55)	2.85 (0.78)	+0.40	0	.031*	0.68 [0.43, 0.87]
	TAM Overall	10	2.84 (0.40)	3.10 (0.53)	+0.26	0	.027*	0.70 [0.42, 0.80]
**Condition 2** Self-Directed Support	*PU*	10	3.10 (0.21)	3.30 (0.48)	+0.20	0	.174	0.43 [0.00, 0.68]
	PEU	10	2.70 (0.63)	3.05 (0.60)	+0.35	2	.086	0.54 [0.07, 0.80]
	ATT	10	3.15 (0.34)	3.45 (0.50)	+0.30	0	.095	0.53 [0.00, 0.74]
	BI	10	2.80 (0.35)	3.20 (0.63)	+0.40	2	.089	0.54 [0.09, 0.80]
	Overall	10	2.94 (0.27)	3.25 (0.49)	+0.31	4	.028*	0.70 [0.14, 0.85]
**Condition 3** Provider-led Support	*PU*	11	2.73 (0.47)	3.32 (0.51)	+0.59	0	.012*	0.76 [0.58, 0.90]
	PEU	11	2.95 (0.57)	3.50 (0.50)	+0.55	6	.046*	0.60 [0.11, 0.91]
	ATT	11	2.82 (0.34)	3.36 (0.50)	+0.54	0	.018*	0.72 [0.50, 0.88]
	BI	11	2.50 (0.22)	3.05 (0.52)	+0.55	4.5	.015*	0.73 [0.38, 0.95]
	Overall	11	2.75 (0.29)	3.31 (0.34)	+0.56	0	.005*	0.85 [0.66, 0.89]

Notes: Pre-post differences were assessed using Wilcoxon signed-rank tests (paired, two-tailed). Effect sizes are reported as $r = Z/\sqrt{N}$. 95% confidence intervals were estimated using bias-corrected and accelerated (BCa) bootstrapping with 2,000 resamples. Asterisk (*) designates statistical significance ($P<.05$). These analyses are exploratory; no correction for multiple comparisons was applied. Results should be interpreted in the context of overall patterns rather than individual construct-level comparisons. TAM scores are based on a Likert scale (range: 1–5).

7. Item-Level System Usability Scale (SUS) Results by Condition

Table S4 presents item-level SUS scores by experimental condition, Two items were omitted from the original 10-item SUS due to limited system complexity: "I found the various functions in this system were well integrated" and "I thought there was too much inconsistency in this system." Item-level scores reflect responses on the original 1–4 Likert scale and are distinct from the composite 0–100 SUS score reported in the main manuscript. Given the modified scale, comparisons to published benchmarks are not made and item-level scores are presented as descriptively only.

Across conditions, Condition 3 demonstrated the highest scores on positively worded items (Ease of Use, General Learnability, Confidence) and the lowest scores on negatively worded items (Complexity, Usability Hindrance), suggesting a pattern of higher usability in the provider-led condition. These patterns should be interpreted cautiously given the exploratory nature of the analysis and the small sample size.

Table S4. Item-level System Usability Scale (SUS) scores by experimental condition.

SUS Item	Overall (SD)	Condition 1 No Support (SD)	Condition 2 Self-Directed Support (SD)	Condition 3 Provider-led Support (SD)
**SUS01 Intention to Use**	3.06 (0.68)	3.00 (0.82)	3.10 (0.74)	3.09 (0.54)
**SUS02 Complexity**	1.77 (0.56)	1.80 (0.63)	1.90 (0.32)	1.64 (0.67)
**SUS03 Ease of Use**	3.23 (0.62)	3.10 (0.57)	3.00 (0.67)	3.55 (0.52)
**SUS04 Need for Assistance**	2.19 (0.65)	2.20 (0.63)	2.20 (0.79)	2.18 (0.60)
**SUS07 General Learnability**	2.81 (0.54)	2.40 (0.52)	2.90 (0.32)	3.09 (0.54)
**SUS08 Usability Hindrance**	1.87 (0.56)	1.90 (0.57)	1.90 (0.74)	1.82 (0.40)
**SUS09 Confidence**	2.71 (0.59)	2.60 (0.52)	2.60 (0.70)	2.91 (0.54)
**SUS10 Individual Learnability**	1.87 (0.56)	2.00 (0.47)	1.80 (0.63)	1.82 (0.60)

Note: Values represent mean (SD) raw item scores on the original Likert 1-4 scale. For positively worded items (SUS01 Intention to Use, SUS03 Ease of Use, SUS07 General Learnability, SUS09 Confidence), higher scores indicate better usability. For negatively worded items (SUS02 Complexity, SUS04 Need for Assistance, SUS08 Usability Hindrance, SUS10 Individual Learnability), lower scores indicate better usability. Two standard SUS items were omitted due to limited system complexity. Item-level results are presented descriptively; given the modified scale, benchmark comparisons are not made. These results are exploratory and descriptive.

8. Exploratory Subgroup Analyses by Participant Demographics

Exploratory subgroup analyses were conducted to examine potential associations between participant demographic characteristics and primary outcomes. These analyses were not corrected for multiple comparisons and are intended to assess potential confounding and to inform future research. Results are reported regardless of statistical significance. With the exception of acceptability scores by age group, no statistically significant associations were identified across subgroup comparisons.

8.1. Technology Level and VR Experience

Technology comfort level was categorized as Low (n=16) or Medium (n=15) based on self-rated comfort with everyday and advanced technologies. Descriptive statistics for engagement and acceptability outcomes by technology level and VR experience level are presented in Table S5a. Participants with low technology comfort spent more time in VRx on average (M=18.06, SD=4.75) compared to those with medium comfort (M=15.87, SD=6.06), and reported higher VR engagement (83.38% vs 66.67%). Acceptability scores were comparable between groups. A Kruskal-Wallis test did not reveal statistically significant differences across technology comfort groups for any engagement or acceptability measure (all P>.05). Self-efficacy and satisfaction by technology level and VR experience are presented in Table S5b.

Table S5a. Descriptive statistics for engagement and acceptability outcomes by technology comfort level and VR experience level (N=31).

Subgroup	n	Mean Time in VRx (min) (SD)	Task Completion Rate (%)	Mean VR Engagement %	Mean Acceptability Score	Mean Acceptability %
**Technology Comfort Level**						
Low	16	18.06 (4.75)	94.11%	83.38%	143.44	84.38%
Medium	15	15.87 (6.06)	95.40%	66.67%	148.67	87.45%
**VR Experience Level**						
No knowledge of VR	2	18.50 (2.12)	95.37%	83.50%	150.00	88.24%
Never used a VR headset	14	16.57 (5.40)	93.94%	69.07%	145.36	85.50%
Somewhat familiar	15	17.20 (5.97)	95.40%	80.00%	146.00	85.88%

Note: Kruskal-Wallis tests did not reveal statistically significant differences across technology comfort groups for any outcome. VR experience group with n=2 should be interpreted with caution.

Table S5b. Self-efficacy and satisfaction scores by technology comfort level and VR experience level (N=31).

Subgroup	n	Mean Self-Efficacy Score (SD)	Mean Satisfaction Score (SD)
**Technology Comfort Level**			
Low	16	2.85 (0.40)	3.56 (0.48)
Medium	15	2.91 (0.52)	3.43 (0.42)
**VR Experience Level**			
No knowledge of VR	2	2.67 (0.00)	3.75 (0.35)
Never used a VR headset	14	2.92 (0.44)	3.53 (0.50)
Somewhat familiar	15	2.87 (0.52)	3.43 (0.42)

Note: Self-efficacy and satisfaction were not formally compared across groups and are reported descriptively.

No statistically significant differences were observed; however, given the small sample size, these findings should not be interpreted as evidence of no effect. Future research with larger and more diverse samples should examine whether technology proficiency, assessed using validated instruments, influences VRx adoption.

8.2. Age Range

Participants were grouped into five age ranges. Descriptive statistics for engagement and acceptability outcomes by age group are presented in Table S5c, and Kruskal-Wallis test results are presented in Table S5d. No statistically significant between-group differences were observed for time in VRx, task completion rate, or VR engagement. Acceptability scores and acceptability percentage differed significantly across age groups (χ^2=10.10, df=4, P=.039, ηH^2=0.235), with younger participants (18–24) reporting the highest acceptability (M=160.00, 94.12%) and the oldest group (55–65) reporting the lowest (M=112.00, 65.88%). Task completion rates remained high across all age groups (range: 92.44%–96.46%). Self-efficacy and satisfaction by age group are presented in Table S5e.

Despite limited statistical significance across most outcomes, observed trends and the significant age-related acceptability finding warrant further investigation in future adequately powered studies.

Table S5c. Descriptive statistics for engagement and acceptability outcomes by age range (N=31).

Age Range	n	Mean Time in VRx (min) (SD)	Task Completion Rate (%)	Mean VR Engagement %	Mean Acceptability Score	Mean Acceptability %
18–24	5	18.00 (4.85)	96.46%	80.00%	160.00	94.12%
25–34	8	16.25 (6.36)	95.90%	66.62%	156.25	91.91%
35–44	9	18.11 (3.37)	94.60%	85.33%	146.67	86.27%
45–54	4	17.25 (3.77)	93.41%	75.00%	148.75	87.50%
55–65	5	15.00 (9.19)	92.44%	66.60%	112.00	65.88%

Note: Descriptive statistics only. Small subgroup sizes (minimum n=4) limit interpretability of condition-level differences.

Table S5d. Kruskal-Wallis test results for engagement and acceptability outcomes by age group (N=31).

Variable	χ^2	df	P Value	ηH^2
Time in VRx	0.63	4	.959	-
Task Completion Rate	3.43	4	.488	-
VR Engagement %	1.12	4	.891	-
Acceptability Score	10.10	4	.039*	0.235
Acceptability %	10.10	4	.039*	0.235

Note: Asterisk (*) designates P<.05. Effect size (ηH^2) reported for significant comparisons only; 0.235 indicates a large effect per conventional benchmarks. No corrections for multiple comparisons were applied. Results are exploratory and should be interpreted cautiously given small subgroup sizes (minimum n=4).

Table S5e. Self-efficacy and satisfaction scores by age range (N=31).

Age Range	n	Mean Self-Efficacy Score (SD)	Mean Satisfaction Score (SD)
18–24	5	2.87 (0.38)	3.50 (0.50)
25–34	8	2.92 (0.30)	3.44 (0.42)
35–44	9	3.04 (0.60)	3.81 (0.37)
45–54	4	2.67 (0.27)	3.38 (0.48)
55–65	5	2.78 (0.62)	3.25 (0.42)

Note: Self-efficacy and satisfaction were not formally compared across groups and are reported descriptively.

8.3. Education Level

Education level was grouped into three categories: High School or Less (n=5), Undergraduate Degree (n=8), and Graduate Degree (n=18). Descriptive statistics are presented in Table S5f. A Kruskal-Wallis test did not reveal statistically significant differences in engagement or acceptability outcomes across education groups. Task completion rates were similar across groups (range: 94.11%–96.43%). Participants with graduate degrees spent the most time in VRx (M=17.89, SD=4.59) and reported the highest self-efficacy (M=3.00, SD=0.44) and satisfaction (M=3.64, SD=0.45). Self-efficacy and satisfaction by education level are presented in Table S5g.

Table S5f. Descriptive statistics for engagement and acceptability outcomes by education level (N=31).

Education Level	n	Mean Time in VRx (min) (SD)	Task Completion Rate (%)	Mean VR Engagement %	Mean Acceptability Score	Mean Acceptability %
High School or Less	5	14.80 (7.12)	96.43%	66.67%	155.00	91.00%
Undergraduate Degree	8	16.38 (6.41)	95.12%	66.67%	130.62	76.75%
Graduate Degree	18	17.89 (4.59)	94.11%	81.56%	150.28	88.22%

Table S5g. Self-efficacy and satisfaction scores by education level (N=31).

Education Level	n	Mean Self-Efficacy Score (SD)	Mean Satisfaction Score (SD)
High School or Less	5	2.87 (0.38)	3.30 (0.45)
Undergraduate Degree	8	2.63 (0.49)	3.31 (0.37)
Graduate Degree	18	3.00 (0.44)	3.64 (0.45)

Note: Self-efficacy and satisfaction were not formally compared across groups and are reported descriptively.

Education level did not emerge as a significant predictor of VR engagement or acceptability. Future research should consider whether education-related differences in expectations or technology familiarity influence adoption in larger, more diverse samples.

8.4. VR Experience Choice: Seated Versus Standing

Participants selected their preferred physical positioning for VR use (seated, n=7; standing, n=24). Descriptive statistics are presented in Table S5h. No statistically significant differences were observed between groups for any engagement, acceptability, or self-efficacy measure. Standing participants spent slightly more time in VRx on average (M=17.46, SD=5.20) compared to seated participants (M=15.43, SD=6.40), while seated participants had a slightly higher task completion rate (96.72% vs 94.15%). Self-efficacy and satisfaction by positioning preference are presented in Table S5i.

Table S5h. Descriptive statistics for engagement and acceptability outcomes by VR experience choice (N=31).

Positioning	n	Mean Time in VRx (min) (SD)	Task Completion Rate (%)	Mean VR Engagement %	Mean Acceptability Score	Mean Acceptability %
Seated	7	15.43 (6.40)	96.72%	61.86%	153.57	90.34%
Standing	24	17.46 (5.20)	94.15%	79.21%	143.75	84.56%

Table S5i. Self-efficacy and satisfaction scores by VR experience choice (N=31).

Positioning	n	Mean Self-Efficacy Score (SD)	Mean Satisfaction Score (SD)
Seated	7	2.95 (0.23)	3.43 (0.35)
Standing	24	2.86 (0.51)	3.52 (0.48)

Note: Self-efficacy and satisfaction were not formally compared across groups and are reported descriptively.

The absence of significant differences between seated and standing participants supports the selection of an application capable of accommodating both positioning preferences, which was identified as a critical usability requirement in earlier phases of this research. These findings suggest that physical positioning did not substantially influence engagement or acceptability outcomes in this sample.

9. Extended BEAR Framework Domain Mapping

Table S6 presents additional BEAR framework constructs that were supported by qualitative evidence from exit interviews but were represented by fewer participant responses or overlapped substantially with constructs presented in the main manuscript (Table 13). These constructs are included for completeness and to inform future research.

Table S6. Extended BEAR framework domain mapping from exit interview qualitative analysis.

BEAR Domain	Summary of Findings	Supporting Evidence
**Social Influences**	Social and peer influences shape willingness to adopt VR-based interventions; skepticism about VR from family and friends noted.	*"My family would think I'm playing video games, not doing therapy" (C1)*
**Intentions**	Stated intentions to use VRx were moderated by practical constraints including cost, time, and device availability. Intention to use may be conditional on perceived clinical need and the specific condition being treated.	*"I'd be open to it if my doctor said to, but I probably wouldn't seek it out on my own" (C2)*<br><br>*"I wouldn't personally use it every day but with a diagnosis, that may make things different" (C1)*
**Role and Identity**	Some participants viewed VR as incongruent with their self-image or daily routines; clinician	*"I don't see myself as a gamer, so putting on a headset felt strange at first" (C1)*

	framing helped reposition VRx as a legitimate health tool.	
**Memory, Attention, Decision Processes**	Cognitive load during initial setup competed with engagement; participants in C3 reported lower cognitive burden during onboarding.	*"There was so much to take in at first that I couldn't focus on the actual experience" (C2)*
**Performance Expectancy**	Expectations about therapeutic outcomes influenced willingness to engage; evidence-based framing increased confidence in efficacy.	*"Once I learned more about the evidence, I can understand more about the purpose and why it worked" (C3)*
**Demographic Characteristics**	Age and technology familiarity shaped initial comfort and learning speed; older participants and those with less technology experience required more support.	*"First touch needs to be positive or people will walk away from using it, especially older people" (C1)*

Note: These constructs had supporting evidence in the qualitative data but were represented by fewer participant responses or overlapped substantially with constructs presented in Table 13 of the main manuscript.

References

1. Leon AC, Davis LL, Kraemer HC. The Role and Interpretation of Pilot Studies in Clinical Research. J Psychiatr Res 2011 May;45(5):626–629. PMID:21035130

2. Sullivan GM, Feinn RS. Facts and Fictions About Handling Multiple Comparisons. J Grad Med Educ 2021 Aug;13(4):457–460. PMID:34434505
