# Supplementary material for: Evaluating the Effects of Clinician Prescribing and Implementation Materials on Adoption of Virtual Reality Therapeutics: Randomized Feasibility Pilot Study
Source: JMIR XR Spat Comput. 2026 Jun 30;3:e90626. doi: 10.2196/90626 (PMC13317682; doi:10.2196/90626)
Supplement: Checklist 1 [file xr-v3-e90626-s007.docx]

# CONSORT 2010 Extension for Randomised Pilot and Feasibility Trials — Completed Checklist [1,2]

| **Section / Topic** | **Item No.** | **Checklist Item** | **Location in Manuscript** |
| --- | --- | --- | --- |
| **Title and abstract** | | | |
|  | 1a | Identification as a randomised trial in the title | *The title identifies the study as a three-arm randomized feasibility pilot study.* |
|  | 1b | Structured summary of trial design, methods, results, and conclusions | *Abstract (Background, Objective, Methods, Results, Conclusions)* |
| **Introduction** | | | |
|  | 2a | Scientific background and explanation of rationale | *Introduction, Background and Rationale section* |
|  | 2b | Specific objectives or hypotheses | *Introduction, Objectives section* |
| **Methods: Trial design** | | | |
|  | 3a | Description of trial design (such as parallel, factorial) including allocation ratio | *Methods, Study Design: Three-arm randomized feasibility pilot study comparing three implementation conditions. Participants were randomly allocated across conditions with a target allocation of approximately 1:1:1 using a Microsoft Excel RAND()-generated allocation sequence.* |
|  | 3b | Important changes to methods after trial commencement (such as eligibility criteria), with reasons | *No changes were made after trial commencement* |
| **Methods: Participants** | | | |
|  | 4a | Eligibility criteria for participants | *Methods, Participants: Eligibility Criteria subsection* |
|  | 4b | Settings and locations where the data were collected | *Methods, Study Setting subsection* |
| **Methods: Interventions** | | | |
|  | 5 | The interventions for each group with sufficient details to allow replication, including how and when they were actually administered | *Methods, Intervention subsection; Multimedia Appendix 5* |
| **Methods: Outcomes** | | | |
|  | 6a | Completely defined pre-specified primary and secondary outcome measures, including how and when they were assessed | *Methods, Data Collection subsection; Table 2* |
|  | 6b | Any changes to trial outcomes after the trial commenced, with reasons | *No changes to outcomes after commencement* |
| **Methods: Sample size** | | | |
|  | 7a | How sample size was determined | *Methods, Study Design: pilot guidance (10–15 per arm, N=30–45 total)* |
|  | 7b | When applicable, explanation of any interim analyses and stopping guidelines | *Not applicable, no interim analyses conducted* |
| **Methods: Randomisation — sequence generation** | | | |
|  | 8a | Method used to generate the random allocation sequence | *Methods, Randomization: An allocation sequence was generated prior to study initiation in Microsoft Excel using the RAND() function, with a target 1:1:1 allocation ratio across the three experimental conditions.* |
|  | 8b | Type of randomisation; details of any restriction (such as blocking and block sizes) | *Simple randomization was used; no formal blocking or stratification was applied. Self-reported technology comfort level and prior VR experience were monitored across conditions during enrollment as baseline characteristics but were not used as allocation variables. Target group sizes were monitored during recruitment; when scheduled participants did not complete their session, a subsequent enrolled participant was assigned to the affected condition to maintain approximate group sizes across arms.* |
| **Methods: Randomisation — allocation concealment mechanism** | | | |
|  | 9 | Mechanism used to implement the random allocation sequence, describing any steps taken to conceal the sequence until interventions were assigned | *Methods, Randomization: Allocation concealment from study personnel was not implemented, as schedulers required visibility of assignments to prepare condition-specific materials in advance of each session. Given the visible differences across study arms, full blinding of participants and study personnel was not feasible. Participants were not informed of their assigned condition until arrival for their study appointment. The absence of allocation concealment and blinding is acknowledged as a limitation.* |
| **Methods: Randomisation — implementation** | | | |
|  | 10 | Who generated the random allocation sequence, who enrolled participants, and who assigned participants to interventions | *Methods, Randomization: The allocation sequence was generated by the study team prior to study initiation. Study team members enrolled participants and assigned them to interventions at the time of scheduling, based on the next available condition in the allocation sequence.* |
| **Methods: Blinding** | | | |
|  | 11a | If done, who was blinded after assignment to interventions (for example, participants, care providers, those assessing outcomes) and how | *Full blinding was not feasible given visible differences across study arms. Participants were not informed of their assigned condition until arrival for their appointment. Study personnel were aware of condition assignments. The absence of blinding is acknowledged as a limitation.* |
|  | 11b | If relevant, description of the similarity of interventions | *Not applicable* |
| **Methods: Statistical methods** | | | |
|  | 12a | Statistical methods used to compare groups for primary and secondary outcomes | *Methods, Statistical Analysis: Wilcoxon signed-rank, Kruskal-Wallis; Multimedia Appendix 8* |
|  | 12b | Methods for additional analyses, such as subgroup analyses and adjusted analyses | *Methods, Statistical Analysis: exploratory subgroup analyses; Multimedia Appendix 8* |
| **Results: Participant flow** | | | |
|  | 13a | For each group, the numbers of participants who were randomly assigned, received intended treatment, and were analysed for the primary outcome | *Figure 3 (CONSORT flow diagram); Results, Participant Characteristics* |
|  | 13b | For each group, losses and exclusions after randomisation, together with reasons | *Figure 3; Results: 8 did not complete (6 logistical, 2 study-related)* |
| **Results: Recruitment** | | | |
|  | 14a | Dates defining the periods of recruitment and follow-up | *Methods, Participants: December 2024 to February 2025* |
|  | 14b | Why the trial ended or was stopped | *Not stopped early; study completed as planned* |
| **Results: Baseline data** | | | |
|  | 15 | A table showing baseline demographic and clinical characteristics for each group | *Table 3* |
| **Results: Numbers analysed** | | | |
|  | 16 | For each group, number of participants (denominator) included in each analysis and whether the analysis was by original assigned groups | *Results, Participant Characteristics — N=31 completed the intervention and were included in the final analysis (Condition 1 n=10, Condition 2 n=10, Condition 3 n=11). Eight enrolled participants did not complete the intervention and were excluded from outcome analyses, with reasons reported in Figure 3 and Results.* |
| **Results: Outcomes and estimation** | | | |
|  | 17a | For each primary and secondary outcome, results for each group, and the estimated effect size and its precision (such as 95% confidence interval) | *Tables 4–12; Multimedia Appendix 8* |
|  | 17b | For binary outcomes, presentation of both absolute and relative effect sizes is recommended | *Not applicable; primary outcomes were continuous/ordinal* |
| **Results: Ancillary analyses** | | | |
|  | 18 | Results of any other analyses performed, including subgroup analyses and adjusted analyses, distinguishing pre-specified from exploratory | *Multimedia Appendix 8: exploratory subgroup analyses by demographic characteristics* |
| **Results: Harms** | | | |
|  | 19 | All important harms or unintended effects in each group | *Results, Tolerability Outcomes: no adverse events; cybersickness minimal and transient* |
| **Discussion: Limitations** | | | |
|  | 20 | Trial limitations, addressing sources of imprecision, and, if relevant, multiplicity of analyses | *Discussion, Limitations section* |
| **Discussion: Generalisability** | | | |
|  | 21 | Generalisability (external validity, applicability) of the trial findings | *Discussion, Limitations: healthy adults in non-clinical settings; clinical populations may differ* |
| **Discussion: Interpretation** | | | |
|  | 22 | Interpretation consistent with results, balancing benefits and harms, and considering other relevant evidence | *Discussion, Principal Results and Interpretation sections* |
| **Other information: Registration** | | | |
|  | 23 | Registration number and name of trial registry | *Not registered: pilot feasibility study; rationale provided in Ethical Considerations* |
| **Other information: Protocol** | | | |
|  | 24 | Where the full trial protocol can be accessed, if available | *IRB-approved protocol available from corresponding author on request* |
| **Other information: Funding** | | | |
|  | 25 | Sources of funding and other support (such as supply of drugs), role of funders | *Funding section: no financial support received* |
| **Pilot extension: Rationale** | | | |
|  | P1 | Justification for a pilot study and description of what it is intended to achieve | *Methods, Study Design; Introduction, Objectives* |
| **Pilot extension: Objectives** | | | |
|  | P2 | Specific objectives and/or research questions for the pilot trial | *Introduction, Objectives: evaluate implementation strategy influence on early adoption* |
| **Pilot extension: Feasibility outcomes** | | | |
|  | P3 | Prespecified criteria for judging feasibility of the future definitive trial and how they will be assessed | *No formal progression criteria were prespecified. Feasibility was assessed descriptively through recruitment, completion, tolerability, fidelity, usability, and engagement outcomes.* |
| **Pilot extension: Progression criteria** | | | |
|  | P4 | Description of progress criteria (for example, stopping rules and decision rules for proceeding to main trial) | *Not applicable; no formal progression criteria or stopping rules were prespecified. Findings are hypothesis-generating and intended to inform future study design.* |

## References

1. Eldridge SM, Chan CL, Campbell MJ, Bond CM, Hopewell S, Thabane L, Lancaster GA. CONSORT 2010 statement: extension to randomised pilot and feasibility trials. BMJ British Medical Journal Publishing Group; 2016 Oct 24;355:i5239. PMID:27777223
2. Hopewell S, Chan A-W, Collins GS, Hróbjartsson A, Moher D, Schulz KF, Tunn R, Aggarwal R, Berkwits M, Berlin JA, Bhandari N, Butcher NJ, Campbell MK, Chidebe RCW, Elbourne D, Farmer A, Fergusson DA, Golub RM, Goodman SN, Hoffmann TC, Ioannidis JPA, Kahan BC, Knowles RL, Lamb SE, Lewis S, Loder E, Offringa M, Ravaud P, Richards DP, Rockhold FW, Schriger DL, Siegfried NL, Staniszewska S, Taylor RS, Thabane L, Torgerson D, Vohra S, White IR, Boutron I. CONSORT 2025 statement: updated guideline for reporting randomised trials. BMJ British Medical Journal Publishing Group; 2025 Apr 14;389:e081123. PMID:40228833
