# Supplementary material for: Evaluating the Effects of Clinician Prescribing and Implementation Materials on Adoption of Virtual Reality Therapeutics: Randomized Feasibility Pilot Study
Source: JMIR XR Spat Comput. 2026 Jun 30;3:e90626. doi: 10.2196/90626 (PMC13317682; doi:10.2196/90626)
Supplement: Checklist 2 [file xr-v3-e90626-s008.docx]

# CONSORT-eHEALTH Checklist V1.6 : Completed Checklist [1]

| **Section / Topic** | **Item No.** | **Checklist Item** | **Location in Manuscript** |
| --- | --- | --- | --- |
| **1. Title and abstract** | | | |
|  | 1a | Title: Identification as web-based or mobile health study | *Title includes 'Virtual Reality Therapeutics'; technology-based intervention clearly identified* |
|  | 1b | Abstract: Structured summary including intervention description, outcomes | *Abstract provides structured summary with Background, Objective, Methods, Results, Conclusions* |
| **2. Introduction** | | | |
|  | 2a | Background: Scientific background and rationale including description and theory of the intervention | *Introduction, Background and Rationale; BEAR framework described; VRx evidence base reviewed* |
|  | 2b | Objectives: Specific objectives or hypotheses | *Introduction, Objectives section* |
| **3. Methods: Trial design** | | | |
|  | 3a | Trial design including allocation ratio | *Methods, Study Design: Three-arm randomized feasibility pilot study comparing three implementation conditions. Participants were randomly allocated across conditions with a target allocation of approximately 1:1:1 using a Microsoft Excel RAND()-generated allocation sequence.* |
|  | 3b | Changes to methods after trial commencement | *No changes after commencement* |
| **3. Methods: Participants** | | | |
|  | 4a | Eligibility criteria | *Methods, Participants: Eligibility Criteria* |
|  | 4b | Settings and locations | *Methods, Study Setting: human factors laboratory and community-based settings* |
| **3. Methods: Interventions** | | | |
|  | 5a | Intervention description: For each group, a full description of the intervention (content, delivery methods, level of human involvement, automated delivery) with sufficient detail to allow replication | *Methods, Intervention; Table 1; Multimedia Appendices 4 and 5* |
|  | 5b | Intervention delivery: How the intervention was actually delivered, and by whom | *Methods, Intervention: VRx delivered via Oculus Quest 2/OpenBrush; Condition 3 included mock clinician consultation* |
|  | 5c | Technical requirements and platform: Software, hardware, operating system requirements | *Multimedia Appendix 5: Oculus Quest 2 (64GB/256GB), OpenBrush application pre-loaded* |
|  | 5d | Intervention fidelity: Procedures to ensure integrity of delivery | *Methods, Intervention: standardized script, trained personnel, checklist-based delivery; Multimedia Appendix 7* |
|  | 5e | Co-interventions: Other interventions or treatments | *No co-interventions; Condition 3 included scripted clinician interaction as part of study design* |
| **3. Methods: Outcomes** | | | |
|  | 6a | Primary and secondary outcome measures, how and when assessed | *Methods, Data Collection; Table 2* |
|  | 6b | Changes to outcomes after commencement | *No changes* |
| **3. Methods: Sample size** | | | |
|  | 7 | How sample size was determined | *Methods, Study Design: pilot guidance; 10–15 per arm; total N=30–45* |
| **3. Methods: Randomization** | | | |
|  | 8–10 | Sequence generation, allocation concealment, implementation | *Methods, Randomization: Simple randomization was used to allocate participants to one of three conditions, with a target 1:1:1 allocation ratio. The allocation sequence was generated in Microsoft Excel using the RAND() function prior to study initiation. No formal blocking or stratification was applied; self-reported technology comfort level and prior VR experience were monitored across conditions during enrollment as baseline characteristics but were not used as allocation variables. Assignments were made by study team members at the time of scheduling, based on the next available condition in the allocation sequence. Target group sizes were monitored during recruitment; when scheduled participants did not complete their session, a subsequent enrolled participant was assigned to the affected condition. Allocation concealment from study personnel was not implemented because condition-specific materials had to be prepared in advance.* |
| **3. Methods: Blinding** | | | |
|  | 11 | Blinding procedures | *Methods, Randomization: Full blinding was not feasible given visible differences across study arms, including the presence of clinician consultation in Condition 3 and physical implementation materials in Conditions 2 and 3. Participants were not informed of their assigned condition until arrival for their appointment. Study personnel were aware of assignments. The absence of blinding is acknowledged as a limitation.* |
| **3. Methods: Statistical methods** | | | |
|  | 12 | Statistical methods for primary, secondary, and additional analyses | *Methods, Statistical Analysis; Multimedia Appendix 8* |
| **4. Results: Participant flow** | | | |
|  | 13 | Flow of participants through each stage including uptake and engagement | *Figure 3 (CONSORT flow diagram); Results, Participant Characteristics* |
| **4. Results: Recruitment** | | | |
|  | 14 | Dates of recruitment and follow-up periods | *Methods, Participants : December 2024 to February 2025* |
| **4. Results: Baseline data** | | | |
|  | 15 | Baseline demographic and clinical characteristics | *Table 3* |
| **4. Results: Numbers analysed** | | | |
|  | 16 | Numbers analysed in each group | *N=31 completed the intervention and were included in the final analysis (Condition 1 n=10, Condition 2 n=10, Condition 3 n=11). Eight enrolled participants did not complete the intervention and were excluded from outcome analyses, with reasons reported in Figure 3 and Results.* |
| **4. Results: Outcomes** | | | |
|  | 17 | Results for each group, effect sizes, precision | *Tables 4–12; Multimedia Appendix 8* |
| **4. Results: Usage and engagement** | | | |
|  | 17b (eHEALTH) | Usage data: Frequency, duration, and intensity of engagement with the eHealth intervention | *Results, Engagement and Satisfaction Results: mean time in VRx by condition (Table 7); Fidelity Outcomes: time-based and task-based adherence (Table 11).* |
| **4. Results: Harms** | | | |
|  | 19 | Harms or unintended effects | *Results, Tolerability : Table 12; no adverse events; low cybersickness* |
| **5. Discussion: Limitations** | | | |
|  | 20 | Limitations including sources of potential bias | *Discussion, Limitations section* |
| **5. Discussion: Generalisability** | | | |
|  | 21 | Generalisability of findings | *Discussion, Limitations : healthy adults; non-clinical setting* |
| **5. Discussion: Interpretation** | | | |
|  | 22 | Interpretation consistent with results | *Discussion, Principal Results and Interpretation sections* |
| **6. Other: Registration** | | | |
|  | 23 | Trial registration number and registry | *Not registered; pilot feasibility study rationale in Ethical Considerations* |
| **6. Other: Protocol** | | | |
|  | 24 | Full trial protocol access | *IRB-approved protocol available on request* |
| **6. Other: Funding** | | | |
|  | 25 | Funding sources | *Funding section : no financial support received* |
| **eHEALTH specific: Intervention description** | | | |
|  | E1 | Brief name and version of the eHealth application | *OpenBrush (open-source VR painting application); Oculus Quest 2 VR headset; described in Methods, Intervention and Multimedia Appendix 4* |
|  | E2 | How the eHealth application was used in the trial (e.g., alone or in combination with other interventions) | *Primary therapeutic medium; supplemented by implementation materials (Conditions 2–3) and clinician consultation (Condition 3)* |
|  | E3 | Overview of how the eHealth application was developed and by whom, including involvement of the target population | *Implementation package development informed by prior qualitative research with clinicians and end users; described in Methods, Intervention* |
|  | E4 | How the eHealth application was made available to participants | *VR headsets provided to participants by study team; pre-loaded with OpenBrush application* |
|  | E5 | To what extent the eHealth application was used as intended (adherence, fidelity) | *Results, Fidelity Outcomes: time-based and task-based adherence (Table 11); observational checklist data (Multimedia Appendix 7).* |

## References

1. Eysenbach G, Group C-E. CONSORT-EHEALTH: Improving and Standardizing Evaluation Reports of Web-based and Mobile Health Interventions. Journal of Medical Internet Research JMIR Publications Inc., Toronto, Canada; 2011 Dec 31;13(4):e1923. doi: 10.2196/jmir.1923
